# Model Linking Plasma and Intracellular Tenofovir/Emtricitabine with Deoxynucleoside Triphosphates

**DOI:** 10.1371/journal.pone.0165505

**Published:** 2016-11-10

**Authors:** Xinhui Chen, Sharon M. Seifert, Jose R. Castillo-Mancilla, Lane R. Bushman, Jia-Hua Zheng, Jennifer J. Kiser, Samantha MaWhinney, Peter L. Anderson

**Affiliations:** 1 University of Colorado, Skaggs School of Pharmacy and Pharmaceutical Sciences, Aurora, CO, United States of America; 2 University of Colorado, School of Medicine, Division of Infectious Diseases, Aurora, CO, United States of America; 3 University of Colorado, Colorado School of Public Health, Department of Biostatistics and Informatics, Aurora, CO, United States of America; Imperial College London, UNITED KINGDOM

## Abstract

The coformulation of the nucleos(t)ide analogs (NA) tenofovir (TFV) disoproxil fumarate (TDF) and emtricitabine (FTC) is approved for HIV-infection treatment and prevention. Plasma TFV and FTC undergo complicated hybrid processes to form, accumulate, and retain as their active intracellular anabolites: TFV-diphosphate (TFV-DP) and FTC-triphosphate (FTC-TP). Such complexities manifest in nonlinear intracellular pharmacokinetics (PK). In target cells, TFV-DP/FTC-TP compete with endogenous deoxynucleoside triphosphates (dNTP) at the active site of HIV reverse transcriptase, underscoring the importance of analog:dNTP ratios for antiviral efficacy. However, NA such as TFV and FTC have the potential to disturb the dNTP pool, which could augment or reduce their efficacies. We conducted a pharmacokinetics-pharmacodynamics (PKPD) study among forty subjects receiving daily TDF/FTC (300 mg/200 mg) from the first-dose to pharmacological intracellular steady-state (30 days). TFV/FTC in plasma, TFV-DP/FTC-TP and dNTPs in peripheral blood mononuclear cells (PBMC) were quantified using validated LC/MS/MS methodologies. Concentration-time data were analyzed using nonlinear mixed effects modeling (NONMEM). Formations and the accumulation of intracellular TFV-DP/FTC-TP was driven by plasma TFV/FTC, which was described by a hybrid of first-order formation and saturation. An indirect response link model described the interplay between TFV-DP/FTC-TP and the dNTP pool change. The *EC*_*50*_ (interindividual variability, (%CV)) of TFV-DP and FTC-TP on the inhibition of deoxyadenosine triphosphate (dATP) and deoxycytidine triphosphate (dCTP) production were 1020 fmol/10^6^ cells (130%) and 44.4 pmol/10^6^ cells (82.5%), resulting in (90% prediction interval) 11% (0.45%, 53%) and 14% (2.6%, 35%) reductions. Model simulations of analog:dNTP molar ratios using IPERGAY dosing suggested that FTC significantly contributes to the protective effect of preexposure prophylaxis (PrEP). Simulation-based intracellular operational multiple dosing half-lives of TFV-DP and FTC-TP were 6.7 days and 33 hours. This model described the formation of intracellular TFV-DP/FTC-TP and the interaction with dNTPs, and can be used to simulate analog:dNTP time course for various dosing strategies.

## Introduction

Tenofovir (TFV) disoproxil fumarate (TDF) and emtricitabine (FTC) are co-formulated as Truvada®, which is approved for human immunodeficiency virus 1 (HIV-1) infection treatment as part of combination antiretroviral therapy, as well as pre-exposure prophylaxis [1]. TDF is a prodrug, which undergoes ester hydrolysis on first pass by the gut and the liver and circulates in plasma as TFV predominantly [2, 3]. TFV and FTC are nucleos(t)ide analogs (NA) of deoxyadenosine monophosphate (dAMP) and deoxycytidine (dC). Each undergoes cellular uptake and anabolism to their active intracellular forms: TFV-diphosphate (TFV-DP) and FTC-triphosphate (FTC-TP) [4]. TFV-DP and FTC-TP compete with corresponding endogenous deoxynucleoside triphosphates (dNTP) at the active site of HIV reverse transcriptase (RT), thus inhibiting genetic material biosynthesis. If incorporated into the proviral DNA, TFV-DP and FTC-TP terminate chain elongation [3].

The accumulation of intracellular (IC) TFV-DP/FTC-TP is presumably driven by plasma TFV/FTC concentrations. However, the formation of intracellular TFV-DP/FTC-TP is complicated, as it requires a hybrid of endocytosis, active transport, diffusion, and enzymatic reactions. [2, 5]. These complexities contribute to nonlinearities in the pharmacokinetics (PK) relationship between plasma and intracellular TFV-DP [5–7]. Currently, many studies have characterized the PK of plasma TFV/FTC [8–15] and intracellular TFV-DP/FTC-TP [16–22], individually. However, only a few PK link models have investigated the relationship between the plasma TFV and the intracellular TFV-DP, all of which used steady-state observations only. As a result, these models were restricted by the lack of accumulation phase data [22–25].

In target cells such as CD4 T-cells, TFV-DP and FTC-TP compete with corresponding natural substrates of HIV RT, which are deoxyadenosine triphosphate (dATP) and deoxycytidine triphosphate (dCTP), respectively. The analog:dNTP molar ratios are associated with antiviral efficacies [19, 23]. However, as NAs, TFV and FTC also have the potential to disturb the dNTP pool, which consists of dATP, dCTP, deoxyguanosine triphosphate (dGTP), and thymidine triphosphate (TTP), given the interactions with the same enzymes in deoxypurine/deoxypyrimidine anabolic and metabolic pathways [26–29].

We previously characterized the dNTP pool reductions during TDF/FTC therapy, in the same participants included in this communication [22, 30], and used non-compartmental analysis (NCA) to describe TFV-DP/FTC-TP concentration-time profiles [22]. However, there is a need to understand the pharmacokinetic-pharmacodynamic (PKPD) relationships and to model non-linearities between plasma and intracellular TFV/FTC along with dNTP changes [19, 20, 31].

Creating such models will enable PK-PD simulations of alternative dosing strategies and interpretations for various clinical scenarios. For example, nonlinear PK introduces difficulties in calculating clinical relevant half-lives via traditional methods. Sahin et al proposed the operational multiple dosing half-life, which is based on simulations of different dosing intervals (tau) [32]. In addition, for PK-PD simulations can be applied to alternative TDF/FTC dosing strategies such as on-demand preexposure prophylaxis (PrEP) studied by Molina et al, who reported an 86% protective rate against HIV-infection using IPERGAY dosing strategy (oral administration of two pre-coitus doses followed by two post-coitus doses) [33]. With dNTP data, PK-PD simulation can use target *in vitro* EC_50_ and EC_90_ for analog:dNTP ratio such as those proposed by Cottrell [31].

The main objective of this study was to develop a PK-PD model that characterized the nonlinearities of TFV-DP and FTC-TP using integrated compartmental PK models linking plasma TFV/FTC with intracellular TFV-DP/FTC-TP concentrations,including changes in dNTPs. The model was then used to estimate operational multiple dosing half-lives (*t*_*1/2*.*op*_) [32] and to simulate the analog:dNTP time profiles for the preexposure prophylaxis (PrEP) IPERGAY dosing [33].

## Materials and Methods

### Study design

This study was a prospective, observational, intensive, PKPD study in HIV-positive and negative adults, conducted at the University of Colorado Anschutz Medical Campus. The study was approved by the Colorado Multiple Institutional Review Board (COMIRB), and participants provided written informed consent before participation (Cell-PrEP; protocol in S2 File; ClinicalTrials.gov Identifier: NCT01040091; study duration: 03/2010–08/2013). HIV-negative subjects received daily oral co-formulated TDF 300 mg (TFV 136 mg) and FTC 200 mg for 30 days and HIV-positive subjects received daily co-formulated TDF 300 mg/FTC 200 mg plus efavirenz (EFV) 600 mg for 60 days. HIV-negative subjects had washout visits on days 35, 45, and 60 and HIV-positive subjects had one follow-up visit on day 60 (therapy was continued in this group). All participants had their baseline sample taken before the initiation of treatment for dNTPs. Subjects were asked to fast overnight, beginning at 10 pm before their dosing visits. On days 1 (first-dose) and 30, blood was collected at 1, 2, 4, 8, and 24 hours post-dose, and on days 3, 7, and 20, blood was collected pre-dose, 2 and 8 hours post-dose. Single blood samples were collected on days 35, 45, and 60 [22, 30]. The sample size was chosen to detect a ~60% increase (63%) in TFV-DP in HIV-negative versus HIV-infected persons using a significance level of 0.05, a power of 80%, a coefficient of variation for TFV-DP of 50%, and a two-sample t-test. This outcome was assessed in another communication [22].

### Bioanalysis

Samples included plasma and peripheral blood mononuclear cells (PBMC). Blood was collected in EDTA tubes for plasma separation. CPT vacutainers were used to isolate PBMC, which were counted with an automated countess® cytometer (Invitrogen^TM^, Thermo Fisher Scientific Corporation, Carlsbad, CA) and lysed [22, 34]. TFV and FTC in blood plasma were assayed using a validated LC/MS/MS methodology with a lower limit of quantification (LLOQ) of 10 ng/mL [35]. TFV-DP and FTC-TP concentrations in cellular lysates were assayed with a validated LC/MS/MS method with an LLOQ of 2.5 fmol/sample for TFV-DP and 0.1 pmol/sample for FTC-TP [34]. The components of the dNTP pool (dATP, dCTP, dGTP, and TTP) were analyzed using a validated LC/MS/MS methodology, with an LLOQ of 50 fmol/sample [36]. For PBMC samples, values were converted to femtomole per million cells (fmol/10^6^ cells, based on the number of cells per sample). The dNTP pool components were quantified in PBMC samples from baseline, 1, 2, 4, 8, 24 hours post-dose on day 1, and 8 hours post-dose in all other visits. TFV/FTC in plasma and TFV-DP/FTC-TP in PBMC were assayed at all time points [22, 30].

### Model development

Concentration-time data were analyzed using the first-order conditional estimation with interaction (FOCEI) method of the nonlinear mixed effects modeling, NONMEM® (version 7.3, ICON plc., Ellicott City, MD, USA). Below limit of quantitation (BLQ) samples were treated as missing [37, 38]. Most BLQ samples for TFV-DP/FTC-TP were in the washout phase; for TFV-DP, 11 at day 60 and one at day 45; for FTC-TP, 19 at day 60, four at day 45; and one at day 35. On occasion, TFV-DP was BLQ within the first dose (five occasions), but other samples in the first dose were quantifiable for the subject (five samples were collected per subject). Plasma was always BLQ in the washout phase (all time points except five FTC at day 35). To develop link models, simultaneous (parameters from two models estimated at the same time) and sequential link (parameters from one model were estimated in advance and fixed) methods were both investigated [39, 40]. The plasma TFV model was linked to the intracellular TFV-DP model, then TFV-DP model was linked to endogenous dATP and dGTP (deoxypurines) models, given TFV is a deoxypurine analog and could affect deoxypurine turnover. The FTC models were linked in a similar fashion except with endogenous dCTP and TTP (deoxypyrimidines), as FTC is a deoxypyrimidine analog and could affect deoxypyrimidine turnover.

Classical one-, two-, and three-compartmental models were investigated to develop plasma TFV/FTC models. The plasma models were then linked to the intracellular TFV-DP/FTC-TP model. Because units from the two models carried different meanings (ng/mL vs. fmol/10^6^ cells), mass movement was avoided. Instead, plasma TFV/FTC concentrations (regarded as “exposure”) were used to stimulate TFV-DP/FTC-TP formation (regarded as “response”). Models tested included the first-order formation model described by Burns et al [24], the saturable formation model described by Duwal et al [6], the indirect response model described by Baheti et al [7], and the saturation (tolerance) model described by Porchet et al [41]. One-, two-, and three-compartmental models were investigated to describe the elimination phase of TFV-DP/FTC-TP.

TFV-DP/FTC-TP concentrations were then linked to the endogenous dNTP pool change (response). The concentrations of TFV-DP/FTC-TP were used to inhibit the zero-order rate constant of the corresponding dNTP production (*k*^*0*^_*in*_). Direct link and indirect response link, as well as *E*_*max*_ models and sigmoidal *E*_*max*_ models (hill coefficient), were investigated [42, 43]. The baseline response (dNTP at time zero, *R*_*0*_) is the determinant of indirect responses and a source of variation in pharmacodynamics analysis [44]. Thus, we assessed the three different estimation methods as described by Dansirikul et al [45] for the endogenous dNTP pool *R*_*0*_ that arose from a single measurement. These were method 1: estimation using deviation (ω^2^) from the population mean baseline values, method 2: estimation using deviation (σ^2^) from the individual observed baseline values, and method 3: estimation using both weighted population mean and individual observed baseline values.

Several random effect models were also evaluated to describe interindividual variability (IIV) and residual variability (RV), such as additive, proportional, and exponential models. To assess the model goodness of fit, the objective function value (OFV) was used, with a lower value indicating a better fit.

Acceptable models were selected based on the following criteria: successful minimization; successful covariate step; absolute value of gradients less than 1 in the last iteration; the %RSE (relative standard error) less than 30% for fixed effects in base model, while %RSE less than 50% for random effects; 95% confidence intervals for fixed and random effects not including 0; model conditional number (generated in covariate step) not exceeding 10^p^ (p: number of parameters); no significant correlation among parameters (r<0.8); and eta bars not statistically significant from zero (p>0.05).

### Covariate selection

The effects of covariates on the model parameters were evaluated. A linear model was used for categorical variables such as HIV-infection status, sex, and race. For continuous variables such as estimated glomerular filtration rate (eGFR), age, weight, and body mass index (BMI), subject variables were centered at medians. Then, linear, natural log, and power models were evaluated [46]. The covariates were assessed by likelihood ratio test (LRT), with a forward selection at α = 0.01 (OFV decreased by 6.64), then a backward elimination at α = 0.001 (OFV increased by 10.8).

### Model evaluation

The goodness of fit diagnostics was generated and visually assessed using Xpose [47] (version 4.5.3, http://xpose.sourceforge.net/) and R (http://www.r-project.org/), which includes plots of observations vs. population predictions, observations vs. individual predictions, absolute values of individual weighted residuals vs. individual predictions, and weighted residuals vs. time (basic diagnostic plots). No substantial pattern was allowed in these diagnostic plots. In addition, an overlay plot of observed values and predicted values vs. time was also used to assess the model performance.

Visual predictive checks (VPC) were performed using NONMEM–PDxPOP (version 5.1, ICON plc., Ellicott City, MD, USA) and R. VPCs were conducted using 100× study population (n = 4000) as the sample size. VPCs of the first-dose (day 1), as well as day 1–60 in the HIV-negative group (to capture washout) for intracellular models (TFV-DP, FTC-TP, dATP, dCTP, dGTP, and TTP), were generated. An overlay plot of observed values as well as 90% percentiles, 80% percentiles, and medians were generated and evaluated. Observations outside 90% confidence intervals were also calculated, and a proportion of less than 15% was considered acceptable.

Model evaluation by bootstrapping (n = 1000) was carried out with PDxPOP and R. The 95% percentiles (2.5% and 97.5%) and the bootstrapping success rates were generated and assessed. A success rate that exceeded 90% was required for an acceptable model.

### Simulations and model applications

To demonstrate the application of the model, simulations were carried out for the calculation of operational multiple dosing half-lives (*t*_*1/2*,*op*_) and the characterization of the change of analog:dNTP molar ratios over time for on-demand PrEP purpose using the IPERGAY doing strategy [32, 33].

Given the nonlinearities of TFV-DP/FTC-TP disposition (see results), simulations were performed to calculate the intracellular PK (TFV-DP/FTC-TP) operational multiple dosing half-lives (*t*_*1/2*,*op*_), proposed by Sahin et al [32]. Simulations of population mean concentrations were performed. For TFV-DP, simulations of different dosing intervals (*tau*) from 1 day to 9 days (graduated by 1 day) were performed. Dosing intervals of 0.2, 0.5, 1, 1.5, 2, and 2.5 days were performed for FTC-TP. A total of 50 doses were simulated to ensure the acquisition of steady-state. Maximum concentrations from the first-dose (*Cmax*,*fd*), maximum concentrations at steady-state (*Cmax*,*ss*), and minimum concentrations at steady-state (*Cmin*,*ss*) were then determined for the different tau values. The operational half-lives were defined as the *tau* where the Cmax,ss:Cmax,fd = 2 (suggesting the dosing interval achieved doubled concentrations at steady-state compared with first-dose), as well as the Cmax,ss:Cmin,ss = 2 (suggesting the dosing interval led to 50% elimination at trough compared with peak concentrations at steady-state).

For the IPERGAY trial design for on demand PrEP dosing strategy, Monte-Carlo simulations (n = 1000) were performed. The IPERGAY dosing consisted of two doses (600/400 mg TDF/FTC), 24 hours to 2 hours before coitus, followed by one dose (300/200 mg TDF/FTC) at 24 hours after coitus and another dose at 48 hours post-coitus. Statistical analysis of simulation results was carried out using SAS® (version 9.4, SAS Institute Inc., Cary, NC, USA). Simulations of dosing relative to a coitus event in a virtual HIV-negative population were set for a time course of one-week (7 days), and data generated in every 0.1 days post-dose, as well as at 10^−4^, 10^−3^ and 10^−2^ days after the first-dose (to capture the initial accumulation). Medians and percentiles (5^th^ percentile (P_5) to 95^th^ percentile (P_95), graduated by 5) of molar ratios of TFV-DP:dATP and FTC-TP:dCTP were calculated, then compared with the CD4 T-cell viral suppression *EC*_*50*_ and *EC*_*90*_ (molar ratio) *in vitro*, reported by Cottrell et al [31].

## Results

### Study demographics

**Fig 1:**
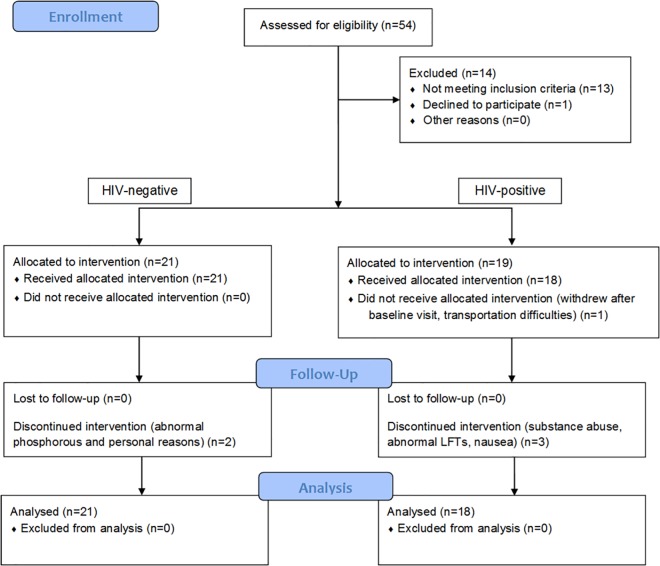
Clinical trial flow chart.

21 HIV-negative adults and nineteen HIV-positive patients (n = 40) were enrolled in the study. Thirteen participants were female, and 27 were male. Nineteen were Caucasians, 16 were African Americans, and five were Hispanic. The median (range) age was 31 (20–52) years. The median eGFR (range) was 93.3 (66.0–131) mL/min/1.73m^2^. The medians (ranges) weight and BMI were 81.1 (56.5–127) kg and 26.6 (19.9–37.7) kg/m^2^, respectively. The clinical trial flow chart is shown in Fig 1. TDF and FTC were well tolerated throughout the study (see Fig 1). 34 (among 40) subjects completed all study visits, two HIV negative and four HIV-positive subjects stopped the study early [22, 30], but all available longitudinal data prior to dropout were included in the model development.

### Population pharmacokinetics modeling of plasma TFV/FTC

**Fig 2:**
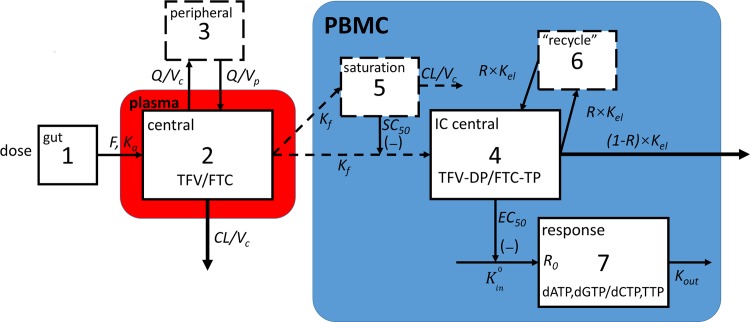
Model schematic. Red box represents plasma. Blue box represents cells. Solid outlines suggest observations available in these compartments. Dashed outlines indicate virtual compartments. Dashed arrows indicate where mass movements were avoided during model development. PBMC: peripheral blood mononuclear cells. IC: intracellular TFV: tenofovir. FTC: emtricitabine. TFV-DP: tenofovir diphosphate. FTC-TP: emtricitabine triphosphate. dATP: deoxyadenosine triphosphate. dGTP: deoxyguanosine triphosphate. dCTP: deoxycytidine triphosphate. TTP: thymidine triphosphate. *F*: bioavailability. *K*_*a*_: absorption rate constant. *Q*: intercompartmental clearance. *V*_*c*_: central compartment volume of distribution. *V*_*p*_: peripheral compartment volume of distribution. *K*_*f*_: first-order formation rate constant of intracellular TFV-DP/FTC-TP stimulated by plasma TFV/FTC. *SC*_*50*_: concentration in a virtual saturation compartment at which formation rate was inhibited by 50%. *R*: recycling portion constant in %. *K*_*el*_: elimination rate constant of intracellular TFV-DP/FTC-TP. *K*^*0*^_*in*_: zero-order production rate constant of intracellular dNTPs. *K*_*out*_: elimination rate constant for intracellular dNTPs. *EC*_*50*_: concentration of intracellular TFV-DP/FTC-TP at which *K*^*0*^_*in*_ is inhibited by 50%.

The best fit was obtained using a classical first-order absorption two-compartmental model (subroutines ADVAN4 TRANS4) for both plasma TFV and FTC. Model structures are illustrated in Fig 2 left portion (compartment 1–3). Differential equations of the plasma model are listed in Equations A-C in S1 File. In these models, bioavailability (*F*) adjusted central compartment volume of distribution (*V*_*c*_*/F*), clearance (*CL/F*), intercompartmental clearance (*Q/F*), and peripheral compartment volume of distribution (*V*_*p*_*/F*) were estimated. The absorption rate constant (*K*_*a*_) was estimated, but a lag time (*t*_*lag*_) was not.

**Fig 3:**
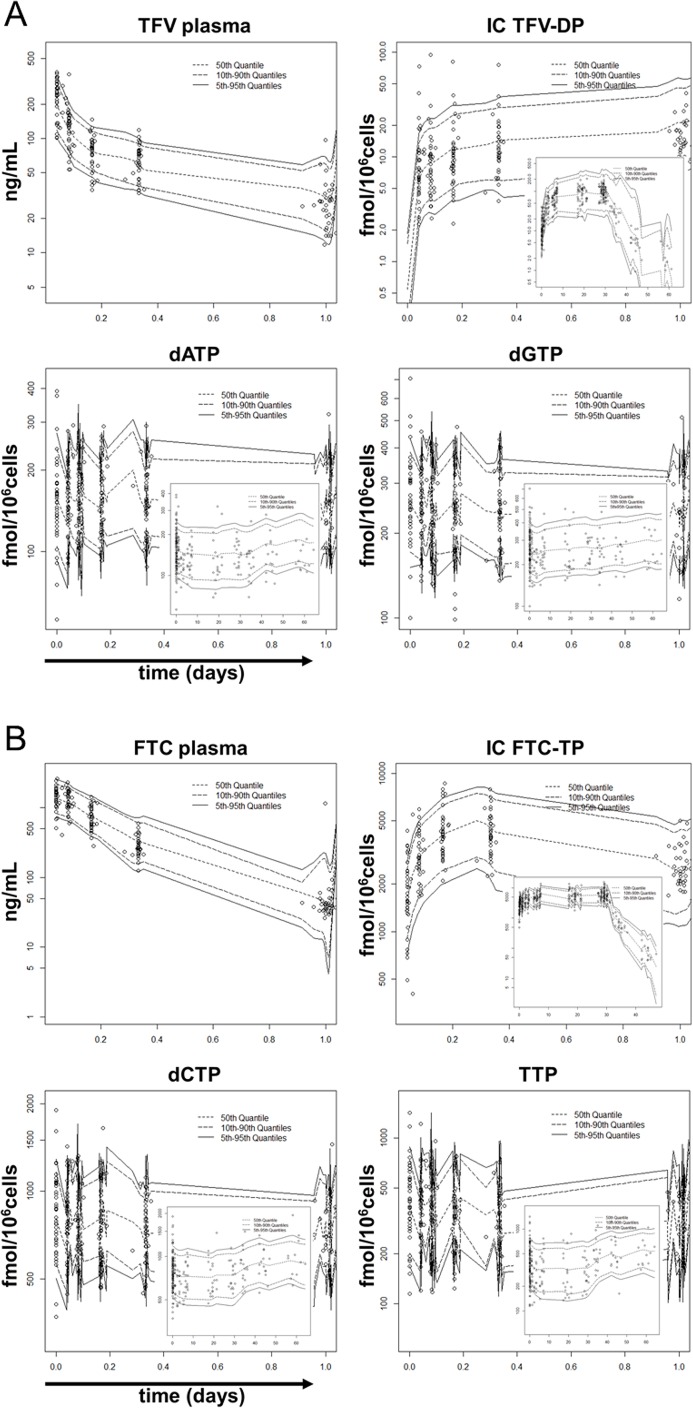
Models visual predictive check. Circles indicate observations. Solid lines indicate model 90% prediction intervals. Dashed lines indicate model estimated medians and 80% prediction intervals. **(A) TFV-deoxypurine models visual predictive check.** The results of the first-dose inserted with the results of the 60 days in the HIV-negative group (had a washout period from day 30–60) for intracellular TFV-DP, dATP and dGTP are shown. TFV: tenofovir. TFV-DP: tenofovir diphosphate. dATP: deoxyadenosine triphosphate. dGTP: deoxyguanosine triphosphate. **(B) FTC-deoxypyrimidine models visual predictive check.** The results of the first-dose inserted with the results of the 60 days in the HIV-negative group (had a washout period from day 30–60) for intracellular FTC-TP, dCTP and TTP are shown. FTC: emtricitabine. FTC-TP: emtricitabine triphosphate. dCTP: deoxycytidine triphosphate. TTP: thymidine triphosphate.

Exponential random effect model on interindividual variability (IIV) and residual variability (RV) resulted in lowest OFV. The IIV for the *V*_*c*_*/F*, *CL/F*, and Q/*F* were estimated in the TFV plasma model. In the FTC plasma model, the IIV in *V*_*c*_*/F*, *CL/F*, and the *V*_*p*_*/F* were estimated. The model estimated terminal half-lives (beta half-life, *t*_*1/2*,*z*_) of TFV and FTC were 17.3 hours (95%CI: 15.7, 19.1) and 26.8 hours (95%CI: 25.6, 28.1). In the FTC plasma model, sex was statistically significant (p = 0.009; ΔOFV = -6.76; ΔIIV_Vc/F_ = -2.8%), as males had 24.4% (95% CI: 7.25%, 41.6%) higher average (typical value, tv) central compartment volume of distribution (*tvV*_*c*_*/F = 99*.*4+24*.*3×sex*; male = 1, female = 0). No subject variable was statistically significant in the TFV plasma model. The goodness of fit plots demonstrated that the model provided a good description of the data (Figures A-C in S1 File). The VPCs are shown in Fig 3A and 3B upper left panels. Model parameters, as well as bootstrapping outcomes, are listed in Table 1. Other model evaluation results are shown in Table A in S1 File.

**Table 1:** Model parameters. IC: intracellular. **(A) TFV-deoxypurine model parameters. (B) FTC-deoxypyrimidine model parameters.**

Parameter	Population Mean		95% Confidence Interval		Interindividual Variability			95% Confidence Interval	
	Estimate	%RSE	NONMEM	Bootstrap	Estimate	%RSE	%CV	NONMEM	Bootstrap
**A**									
**TFV plasma**									
K_a_ (day^-1^)	80.1	13.5	58.9, 101	56.9, 124	-	-	-	-	-
V_c_/F (L)	390	10.1	313, 467	295, 465	0.288	20.8	53.7	0.171, 0.405	1.17E-9, 0.435
CL/F (L/day)	1410	4.94	1270, 1550	1290, 1540	0.117	29.3	34.2	0.0498, 0.184	0.0588, 0.184
V_p_/F (L)	877	6.29	769, 985	785, 1060	-	-	-	-	-
Q/F (L/day)	5390	5.96	4760, 6020	4580, 6310	0.0693	49.2	26.3	0.0246, 0.136	0.00375, 0.221
σ (exponential)	-	-	-	-	0.0745	20.1	27.3	0.0451, 0.104	0.0498, 0.107
**TFV-DP IC**									
K_f_ (day^-1^)	1.4	19.1	0.877, 1.92	0.872, 2.39	0.238	41.5	48.8	0.0444, 0.432	0.0994, 0.444
SC_50_TFV_	6.55	11.6	5.06, 8.04	5.08, 9.94	-	-	-	-	-
K_el_ (day^-1^)	0.228	11.8	0.175, 0.281	0.181, 0.311	0.316	37	56.2	0.0867, 0.545	0.0984, 0.587
R (%)	5.82	11.9	4.47, 7.17	4.63, 8.86	-	-	-	-	-
σ (proportional)	-	-	-	-	0.115	9.22	33.9	0.0942, 0.136	0.0936, 0.136
**dATP**									
R_0_ (fmol/10^6^cells)	155	4.08	143, 167	142, 167	0.220	11.0	46.9	0.173, 0.267	0.173, 0.27
EC_50,TFV-DP_ (fmol/10^6^cells)	1020	7.66	361, 2890	464, 14700	1.70	40.9	130	0.336, 3.06	0.465, 33.4
σ (exponential)	-	-	-	-	0.258	4.92	50.8	0.233, 0.283	0.232, 0.279
**dGTP**									
R_0_ (fmol/10^6^cells)	245	4.86	222, 268	241, 273	0.203	14.3	45.1	0.146, 0.26	0.128, 0.209
EC_50,TFV-DP_ (fmol/10^6^cells)	54.6	18.8	12.4, 240	14.3, 268	-	-	-	-	-
gamma	0.928	26.4	0.448, 1.41	0.46, 1.8	-	-	-	-	-
σ (exponential)	-	-	-	-	-	4.76	51.7	0.242, 0.292	0.234, 0.284
**B**									
**FTC plasma**									
K_a_ (day^-1^)	55.7	13.8	40.6, 70.8	41.9, 2.70E+9	-	-	-	-	-
Vc/F (L)	99.4	5.91	87.9, 111	87.2, 112	0.0319	29	17.9	0.0138, 0.05	0.0112, 0.0481
CL/F (L/day)	482	3.98	444, 520	440, 514	0.0942	45	30.7	0.0111, 0.177	0.0214, 0.186
V_p_/F (L)	166	13.5	122, 210	107, 239	0.0335	43.6	18.3	0.00488, 0.0621	2.13E-50, 1.25
Q/F (L/day)	141	8.94	116, 166	110, 191	-	-	-	-	-
Sex on V_c_/F (linear)	24.3	35.9	7.21, 41.4	6.21, 41.6	-	-	-	-	-
σ (exponential)	-	-	-	-	0.122	20.5	34.9	0.073, 0.171	0.0781, 0.175
**FTC-TP IC**									
K_f_ (day^-1^)	41.6	9.52	33.8, 49.4	35.6, 52.2	0.0358	42.2	18.9	0.0062, 0.0654	0.0101, 0.0677
SC_50_FTC_	3320	12.2	2530, 4110	2590, 4120	-	-	-	-	-
K_el_ (day^-1^)	1.6	4.89	1.45, 1.75	1.43, 1.74	0.0561	35.8	23.7	0.0167, 0.0955	0.0207, 0.101
R (%)	16.0	11.4	12.4, 19.6	13.0, 21.1	-	-	-	-	-
HIV on K_f_ (Linear)	31.3	26.9	14.8, 47.8	15.4, 50.3	-	-	-	-	-
σ (proportional)	-	-	-	-	0.0942	10.6	30.7	0.0747, 0.114	0.0751, 0.116
**dCTP**									
R_0_ (fmol/10^6^cells)	771	3.97	711, 831	716, 825	0.195	10.8	44.2	0.154, 0.236	0.153, 0.239
EC_50_FTC-TP_ (fmol/10^6^cells)	44400	2.86	24300, 80800	26900, 98700	0.68	42.1	82.5	0.118, 1.24	3.81E-8, 1.43
σ (exponential)					0.229	5.2	47.9	0.206, 0.252	0.205, 0.253
**TTP**									
R_0_ (fmol/10^6^cells)	335	9.67	271, 399	271, 396	0.383	10.8	61.9	0.302, 0.464	0.293, 0.468
EC_50_FTC-TP_ (fmol/10^6^cells)	18800	2.69	11200, 32900	11800, 32900	1.02	39	101	0.24, 1.80	0.325. 2.1
σ (exponential)	-	-	-	-	0.356	4.04	59.7	0.328, 0.384	0.327, 0.385

### Population pharmacokinetics modeling of TFV-DP/FTC-TP

Individual PK parameters from the plasma models were fixed for the intracellular TFV-DP/FTC-TP model development (sequential link). Model structures are illustrated in Fig 2 upper right portion (compartment 4–6). Differential equations for intracellular TFV-DP/FTC-TP models are listed in Eqs 1–3.

$$\text{IC central}:\;\frac{dA(4)}{dt}=\frac{K_{f}\times \frac{A(2)}{V_{c}}}{\left(1+\frac{A(5)}{{SC}_{50}}\right)}-K_{el}\times A(4)+A(6)\times K_{el}\times R$$ (1)

$$\text{saturation}:\;\frac{dA(5)}{dt}=\frac{A(2)}{V_{c}}\times K_{f}-A(5)\times \frac{CL}{V_{c}}$$ (2)

$$“\text{recycle}”:\;\frac{dA(6)}{dt}=A(4)\times K_{el}\times R-A(6)\times K_{el}\times R$$ (3)

A hybrid of first-order formation and saturation link model best described the data. In this model, a saturation compartment (compartment 5) was used, in which high plasma concentrations (*A(2)/V*_*c*_) slowed the first order rate constants of TFV-DP/FTC-TP formation (*K*_*f*_). The elimination rate constant in the saturation compartment was fixed to the plasma central compartment elimination rate constant (*CL/V*_*c*_) to avoid overparameterization, assuming that the saturation effect disappeared based on plasma TFV/FTC disappearance. An *SC*_*50*_ was introduced in the saturation model, which indicated the concentration in the virtual saturation compartment that leads to 50% inhibition of TFV-DP/FTC-TP formation. The stimulation constant for both the saturation compartment and the TFV-DP/FTC-TP compartment (IC central, compartment 4) was estimated with the same rate constant of formation (*K*_*f*_*)* to avoid overparameterization. A two-compartmental model best described TFV-DP and FTC-TP elimination. This was modeled with a “recycling” compartment (compartment 6). Due to the collinearity (r>0.9) between *K*_*46*_, *K*_*64*_ (rate of movement from compartment 4 to 6, 6 to 4)_,_ *and K*_*40*_ (elimination rate of compartment 4), a “recycling” ratio constant (%*R*, ranged from 0 to 1) was used. Given the high correlation, the intercompartmental distribution rate constants (*K*_*46*_ and *K*_*64*_) between the IC central (compartment 4) and the “recycle” compartment (compartment 6) were defined as *R×K*_*el*_, and the IC central elimination rate constant (*K*_*40*_) defined as *(1-R)×K*_*el*_. In effect, this modeling created a second elimination phase for TFV-DP and FTC-TP, consistent with a “recycling” (re-phosphorylation) or a “deep” (slow turnover) compartment.

The model used user-defined differential equations (subroutines ADVAN13 TRANS1). TFV-DP and FTC-TP shared the same model structure. The exponential random effect model on IIV and proportional random effect model on RV resulted in the lowest OFV. The IIV was estimated in *K*_*f*_ and *K*_*el*_ for IC central compartment in both TFV-DP and FTC-TP models. In the intracellular FTC-TP model, HIV infection status was found to be statistically significant in regard to the formation rate constant (*tvK*_*f*_ *= 41*.*6+31*.*3×HIV*; positive = 1, negative = 0), as HIV-infected patients had 75.2% (95%CI: 35.6%, 115%) higher average formation rate (p<0.00001; ΔOFV = -20.5; ΔIIV_Kf_ = -5.6%). No statistically significant subject variable was observed in the TFV-DP model.

The alpha half-lives (*ln2/λ*_*1*_, *where λ*_*1*_ *is the slope of the concentration decline in the initial phase*) of TFV-DP and FTC-TP were 73.4 hours (95%CI: 61.8, 87.1) and 8.8 hours (95%CI: 8.2, 9.4). The beta half-lives (associated with compartment 6, *t*_*1/2*,*z*_, *ln2/λ*_*2*,_ *where λ*_*2*_ *is the slope of the concentration decline in the terminal phase*) were 55.6 days (95%CI: 46.9, 66.0) and 76.8 hours (95%CI: 71.7, 82.2). However, these beta half-lives only represented a small fraction of concentration-time profiles, as the “recycling” ratios for the description of the second elimination phase were only 5.82% and 16.0% (see Table 1A and 1B). The goodness of fit plots showed good descriptions of the data (Figures A, D and E in S1 File). The VPCs are illustrated in Fig 3A and 3B upper right panels. Model parameters and bootstrapping outcomes are listed in Table 1A and 1B middle portions. Other model evaluation results are shown in Table A in S1 File.

### Population pharmacodynamics modeling of dATP/dGTP and dCTP/TTP

To develop dNTP models, individual PK parameters of TFV-DP/FTC-TP models were first fixed (sequential link). In the previous study of this data, dNTPs were shown to decrease during TDF/FTC treatment [30]. Thus, intracellular TFV-DP/FTC-TP models were linked to the reduction of the zero-order production rate constant (*K*^*0*^_*in*_) of the corresponding intracellular endogenous dNTPs. The indirect response *E*_*max*_ model best described the data. An *EC*_*50*_ was introduced in the model, which indicated concentrations of TFV-DP/FTC-TP that led to 50% inhibition of the corresponding dNTP production, and the typical values were estimated as *tvEC*_*50*_ *= EXP(THETA)* to improve precision and to ensure positive estimations. Model structures are shown in Fig 2 lower right portion, as response compartment (compartment 7). The equation was (Eq 4):

$$\frac{dA(7)}{dt}=K_{in}^{0}\times (1-\frac{E_{max}\times A(4)}{A(4)+{EC}_{50}})-A(7)\times K_{out}$$ (4)

To avoid overparameterization, the elimination rate constant (*K*_*out*_), as well as maximum drug effect (*E*_*max*_) were fixed to 1. *K*^*0*^_*in*_ and the baseline dNTP (*R*_*0*_) were interconvertible (*K*^*0*^_*in*_ *= R*_*0*_*×K*_*out*_). To estimate the initial individual status of response compartment (compartment 7), in other words, baseline level of dNTP, the model below was chosen (Eq 5).

$$R0i=(\hat{R0}∙\frac{\sigma^{2}}{\omega^{2}+\sigma^{2}}+R0i,o∙\frac{\omega^{2}}{\omega^{2}+\sigma^{2}})∙e^{\eta i,RV∙\frac{\omega^{2}}{\omega^{2}+\sigma^{2}}}$$ (5)

Where *R0i* is the model estimated individual baseline level of dNTP. (R0)^ is the model estimated population mean level of dNTP. *R0i*,*o* is the observed individual dNTP level at baseline. *ω* is the standard deviation (sd) of model estimated IIV of dNTP level at baseline. *σ* is the sd of model estimated residual error of dNTP level throughout the observation period (0–60 days). *ηi*,*RV* represents the random effect of residual error (a normal distribution with mean = 0 and sd = *σ*). The effect on dGTP waned over time, and this was described using a sigmoidal *E’*_*max*_ *~ time* model. However, due to the small sample size and high data variability, this term was simplified to *1/(1+time*^*γ*^*)* (Equation D in S1 File, where γ is a power exponent that modifies the effect of time mathematically).

The best fit was obtained using the user defined model (subroutines ADVAN13 TRANS1). For dATP, dCTP, and TTP models, the IIV was estimable on *R*_*0*_ and *EC*_*50*_. For the dGTP model, the IIV was only estimable for *R*_*0*_. The exponential random effect model on IIV and RV achieved the lowest OFV. No subject covariate was found to be statistically significant. The goodness of fit plots showed a good description of the data (Figures A and F-I in S1 File). The VPCs are illustrated in Fig 3A and 3B lower panels. Model parameters and the bootstrapping 95% CIs are listed in Table 1A and 1B lower portions. The model estimated (median (5% and 95% percentile)) decreases were 11% (0.45%, 53%) in dATP; 14% (2.6%, 35%) in dCTP and 24% (4.5%, 62%) in TTP. The dGTP reached a low point around day 2.5 with a reduction of 13% (6.9%, 21%), then returned to the baseline value; other components in the dNTP pool returned to baseline during the washout period. Other model evaluation results are shown in Table A in S1 File.

### Simulations and model applications

#### Calculation of operational multi-dosing half-lives

**Fig 4:**
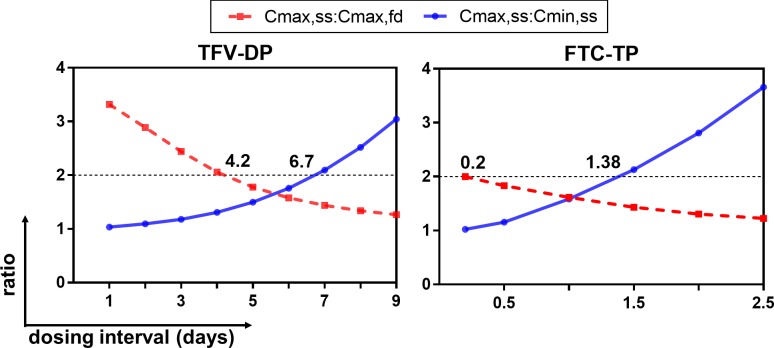
Intracellular operational multiple dosing half-lives. Cmax,fd: maximum concentration at first -dose. Cmax,ss: maximum concentration at steady-state. Cmin,ss: minimum concentration at steady-state. TFV-DP: tenofovir diphosphate. FTC-TP: emtricitabine triphosphate. Y-axis: ratios. X-axis: dosing interval. Dashed lines in red represent the curve of Cmax,ss:Cmax,fd. Solid lines in blue represent the curve of Cmax,ss:Cmin,ss. X values at which dashed lines or solid lines intercept at y = 2 represent operational multiple dosing half-lives.

Given nonlinear PK relationships between plasma TFV/FTC and intracellular TFV-DP/FTC-TP, an operational half-life (*t*_*1/2*,*op*_) was determined [32]. Simulations were performed to generate ratios (*Cmax*,*ss*:*Cmax*,*fd* and *Cmax*,*ss*:*Cmin*,*ss*) from different dosing intervals (*tau*). The curves generated from these ratios are illustrated in Fig 4. The nonlinearity of TFV-DP and FTC-TP accumulation resulted in the interception of the two curves at ratio≠2. The intercepts at x-axis where *Cmax*,*ss*:*Cmax*,*fd* = 2 and *Cmax*,*ss*:*Cmin*,*ss* = 2 were defined as the multiple dosing half-lives (*t*_*1/2*,*op*_). Based on *Cmax*,*ss*:*Cmax*,*fd* = 2, the *t*_*1/2*,*op*_ of TFV-DP and FTC-TP were 4.2 days and 4.8 hours. Based on *Cmax*,*ss*:*Cmin*,*ss* = 2, the *t*_*1/2*,*op*_ of TFV-DP and FTC-TP were 6.7 days and 33 hours.

#### Simulations of IPERGAY trial design

**Fig 5:**
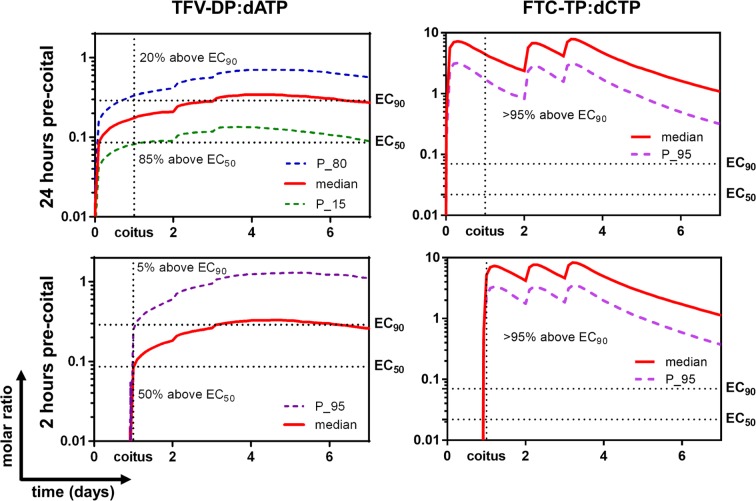
Protection with pre- and post-coital PrEP dosing. Left panels illustrate TFV-DP:dATP molar ratios. Right panels illustrate FTC-TP:dCTP molar ratios. All simulations included another two doses at 24 hours and 48 hours after coitus. Upper panels illustrate 24 hours dosing pre-coitus. Lower panels illustrate 2 hours dosing pre-coitus. P_n: n^th^ percentile. TFV-DP: tenofovir diphosphate. FTC-TP: emtricitabine triphosphate. dATP: deoxyadenosine triphosphate. dCTP: deoxycytidine triphosphate.

Given the importance of analog:dNTP on NA efficacy, and findings of reduced dNTPs over time, simulations of medians and selected percentiles of analog:dNTP ratios for the IPERGAY trial design are illustrated in Fig 5.

When the dose was taken 24 hours pre-coitus, the model predicted that 85% of the virtual population had a TFV-DP:dATP molar ratio above EC_50_ (0.086) at the time of coitus and this persisted for 7 days. The percentage dropped to 50% for 2 hours pre-coital dosing. When evaluating using *EC*_*90*_, 20% of simulated individuals were above EC_90_ when dosing was 24 hours pre-coitus, but this dropped to 5% when the dose was taken 2 hours before coitus, illustrating the slow accumulation of TFV-DP.

At the time of coitus, virtually all individuals (>95%) had FTC-TP:dCTP above *EC*_*90*_ (0.07) and *EC*_*50*_ (0.022), and this persisted for at least seven days, regardless of the timing of the pre-coital dosing.

## Discussion

The PK links between plasma TFV/FTC and intracellular TFV-DP/FTC-TP are complicated due to nonlinearities. The accumulation phases are driven by endocytosis, diffusion, and active transport of plasma TFV/FTC, as well as multiple kinases and phosphorylases that orchestrate the intracellular phosphorylation and dephosphorylation [2, 5]. Duwal et al [6], Baheti et al [7], and Burns et al [24] successfully described steady-state kinetics of TFV-DP/FTC-TP using a saturable formation model, an indirect response model, and a first-order formation model. However, all these models failed to minimize in our study, which included observations from first-dose to steady-state. The reason may be that plasma TFV/FTC fluctuates in roughly similar (little accumulation) and wide ranges, whereas intracellular TFV-DP/FTC-TP slowly accumulates. When plotting “exposure” (plasma TFV/FTC) vs “response” (intracellular TFV-DP/FTC-TP) from first-dose to steady-state, we observed “handshape” relationships (examples are shown in Figure J in S1 File) instead of “S-shape” relationships (suggesting direct effects) or hysteresis loops (suggesting indirect effects), similar to observations of Porchet et al for nicotine tolerance [41]. These “handshape” relationships suggested saturation of phosphate formation for high plasma concentrations, as previously described by Madrasi et al [5]. This effect hints at a constantly changing saturable formation rate of intracellular TFV-DP/FTC-TP by plasma TFV/FTC. We addressed this with a novel TDF/FTC PK model link method—a hybrid of first-order formation and saturation—to describe the formation of intracellular TFV-DP/FTC-TP. However, these models might be limited by the coadministration of TDF/FTC in our study, which limited the ability to assess potential interactions *in vivo*, as some *in vitro* studies suggest a metabolic interaction of intracellular TFV-DP and FTC-TP [48]. Studies with the individual drugs are needed to address potential intracellular interactions.

In addition, a possible “recycling” or “deep” compartment for TFV-DP/FTC-TP distribution was identified, manifesting as biphasic elimination. The complex elimination processes are driven by cell trafficking from peripheral tissue with peripheral blood, as well as multiple enzymes that phosphorylate/dephosphorylate nucleoside analogs [49–52]. Triphosphates that are eliminated (dephosphorylated) may be re-phosphorylated. The biphasic elimination was detected during model development, as a one-compartmental model underpredicted the day 15–30 data in the washout period (day 45–60 of the overall study). As a result, a “recycle” compartment (IC peripheral, compartment 6) was introduced in our model. A three-compartmental model did not converge, due to limited sampling and overparameterization. A two-compartmental model (utilizing compartment 6) best described the data, assuming that part (%R, range from 0 to 1) of the eliminated intracellular TFV-DP/FTC-TP “recycled” (“re-phosphorylated”), or reappeared from a “deep” compartment back into the IC central compartment (compartment 4). However, this compartment was described via a mathematical approach, and the biological interpretation is limited by the study duration (30 doses in HIV-negative group), sparse washout period sampling (5, 15, and 30 days in washout in HIV-negative group only), and the possible influence of the treatment of samples that were below the limit of quantitation (BLQ) as missing, which is a loss of information reducing power and potentially biasing estimates for this portion of the curve [37,38]. We chose not to impute BLQ values (eg between 0 and the LLOQ) as this may also lead to bias [38]. Further study of this “deep” compartment is needed.

During model link development, simultaneous and sequential link methods were both assessed. Simultaneous link models did not minimize after >48 hours of computation (intel® Xeon® Processor E5-2637 v3 at 3.50 Ghz). Therefore, we chose the sequential link method to develop the model link, as these approaches show similar robustness [40].

These models detected two covariates that were statistically significant. In the plasma FTC model, the male participants had a higher volume of distribution of the central compartment (compartment 2) compared with female participants, which might be explained by the higher lean weight in males into which FTC distributes. In the intracellular FTC-TP model, HIV-positive individuals had a higher FTC-TP *K*_*f*_ but not *K*_*el*_ compared with HIV-negative individuals. Seifert et al reported that FTC-TP area under the concentration-time curve (AUC) was higher at the first-dose in HIV-infected patients (first-dose concentration is affected by *K*_*f*_), but this effect disappeared at steady-state (presumably *K*_*el*_ is rate limiting at steady-state concentrations) [22]. Our study was limited by small sample size (n = 40) and sampling time, which did not allow us to estimate the IIV for some parameters (e.g. *K*_*a*_ and *SC*_*50*_) in the model and to detect statistically significant effects of subject covariates, particularly the HIV-infection status. Other studies suggest lower intracellular NA triphosphates in HIV-negative individuals, so further work is needed to better define such effects [53].

For plasma TFV/FTC model development, a classical two-compartmental model was used, similar to previous studies [6, 7, 11, 14, 15, 24, 25]. The TFV PK parameters were within the range of literature [7, 24, 25]. Compared with Valade et al [14, 15], FTC PK parameters were all higher (*CL/F* (L/day): 482 vs 362; *Vc/F* (L): 99.4 vs 42.3; *Vp/F* (L): 166 vs 55.4) except Q/F was similar (141 vs 138 (L/day)). *K*_*a*_ was reported to be difficult to estimate [6, 14, 15, 24, 25]. However, in our study, even with sampling starting at 1 and 2 hours (TFV t_max_:0.5–3.0 hours, FTC t_max_: 1.5–3.0 hours [2, 9, 54–56]), *K*_*a*_ estimation was possible, although *t*_*lag*_ was not [7, 11]. In bootstrapping analysis, extreme values in 95% CI were observed for a few parameters (Table 1A and 1B), including the upper bound of *K*_*a_FTC*_, and the lower bounds of IIV for *V*_*c*_*/F*_*TFV*_, V_p_/F_FTC_ and *EC*_*50_*FTC-TP,dCTP_. These results suggest possible limitations in the sampling times and sample size of our study. To assess the effect of parameter instabilities in bootstrapping analysis, sensitivity analyses were performed by fixing *K*_*a_FTC*_ = 55.7/day and not estimating the IIV for *V*_*c*_*/F*_*TFV*_, V_p_/*F*_*FTC*_ and *EC*_*50_*FTC-TP,dCTP_. Similar model parameter estimations were obtained (%difference<27.6) and the general conclusions of these models remained.

We previously found reduced dNTPs in this study cohort but had not linked drug concentrations to these effects [30]. In this analysis, PKPD models linking the TFV-DP–dATP/dGTP and the FTC-TP–dCTP/TTP were developed using an indirect response model. Given the limited sample size and high variability in dNTPs, several PD parameters (*E*_*max*_ *and K*_*out*_) were fixed to 1 to avoid overparameterization. Because the PD model heavily depended on the baseline response (initial status of compartment 7), to effectively estimate the baseline response, different baseline models were assessed as described by Dansirikul et al [45]. This approach incorporated both the population means and observed values into the estimation, which best described the data. Future studies should consider repeated measurements prior to treatment initiation to improve the estimation of baseline. In the TFV-DP–dGTP link model, a transient effect that reduced over time was observed [30]. This may represent purine nucleoside phosphorylase (PNP) inhibition by intracellular TFV phosphates which would theoretically raise dGTP [28]. However, a larger sample size is required to probe these responses of dGTP in the future.

The half-lives of TFV and FTC were within the range of previous studies [10, 12, 18, 57–59] (TFV: 11.6–31 h; FTC: 3.0–40.5 h). The alpha half-life of TFV-DP (73 hours) was within the range of literature (53.3–125 hours[6, 7, 24]). The beta half-life (55.6 days) may be an overestimation as it was limited by the sparse sampling in washout phase and the approach of handling BLQ as missing values. However, it only accounted for a small portion (*R*_*TFV-DP*_ = 5.8%) of the concentration change in the washout. Non-compartmental analysis (NCA) reported a half-life of 106 hours (TFV-DP) and 54 hours (FTC-TP) in these same HIV-negative subjects in the 30-day washout (n = 17) [60]. Others reported similar half-lives in prolonged observations in washout phase. For example, Hawkins et al, in their trial with a 28-day TFV-DP washout, reported a half-life of 150 h. Dickinson et al reported 116 h (TFV-DP) and 37 h (FTC-TP) half-lives in their 7-day washout observation[18]. Jackson et al reported 164 h (TFV-DP) and 39 h (FTC-TP) in their 10-day washout observation [12]. In a two-compartmental model, the apparent half-lives are the mixture of alpha and beta half-lives. Given this and the non-linearities, the simulation based operational multiple dosing half-lives (*t*_*1/2*,*op*_) might be informative.

Models enabled us to better investigate these complexities by applying the *t*_*1/2*,*op*_ calculation proposed by Sahin et al [32]. They recommended generating both curves of Cmax,ss:Cmin,ss and Cmax,ss:Cmax,fd from different dosing intervals (*tau*), and showed that for linear systems, the two curves will intercept at ratio = 2 (such as plasma diazepam [32]). In our study, the interception did not occur at ratio = 2 due to the nonlinearities, resulting in two *t*_*1/2*,*op*_. Nonlinearities in accumulation favors a higher first-dose concentration (Cmax,fd), and a lower steady-state concentration (Cmax,ss), resulting in a lower position of the Cmax,ss:Cmax,fd curve. Results of *t*_*1/2*,*op*_ calculated from Cmax,ss:Cmin,ss = 2 were comparable to current literature. The *t*_*1/2*,*op*_ (161 hours) of intracellular TFV-DP supports the prolonged viral suppression for up to a week after the treatment discontinuation, reported by Deeks et al [61], and is also similar to the 150 hours reported by Hawkins et al [21] and 180 hours reported by Pruvost et al [62]. The *t*_*1/2*,*op*_ for FTC-TP (33 hours) is also comparable to other literature values (37–39 hours) [12, 18, 59].

These models also enabled simulations of the analog:dNTP time courses for IPERGAY dosing [33]. Models estimated that two tablets pre-coitus plus two tablets post-coitus had 5–20% of >90% protective effect and 50–85% of >50% protective effect from TDF. The relatively slow accumulation of TFV-DP on day 1 may be a disadvantage of TDF for on demand PrEP, but the FTC-TP:dCTP ratios were well above *EC*_*90*_ after the first-dose. This suggests that FTC contributes significantly to the protective on demand PrEP effect of TDF/FTC and is consistent with 86% efficiency from IPERGAY. In these simulations, drug concentrations dominated the molar ratio (analog:dNTP) change, while the relatively small changes in dNTPs did not contribute much to the ratio change. This simulation is limited by the assumption of a simple/direct relationship between analog:dNTP and efficacy, and that the intracellular data were generated from PBMC only. TFV-DP/FTC-TP levels may differ among cell types, such as in tissue mononuclear cells in rectal and female genital tissues that are the major target for PrEP [31, 63]. The dNTP pool also varies among different cell types [31]. Thus, our simulation in PBMC might not represent the cells in target tissues. Nevertheless, PBMC includes CD4 T-cells, which is one of the major target cells for HIV and antiretroviral agents. Also, model development was based on oral dosing, thus simulations may not extrapolate to topical dosing scenarios, in which tissues, concentration gradients, and model structure are different, and other biological factors may be important to consider, such as local pH and the microbiome.

In conclusion, we developed PKPD models linking plasma and intracellular TFV/FTC with the dNTP pool. Observations from first-dose to steady-state were necessary for model development to describe complex accumulation processes. A hybrid of first-order formation and saturation was used to describe nonlinearities of intracellular TFV-DP/FTC-TP formation. An indirect response model was used to describe the interaction between TFV-DP/FTC-TP and dNTPs. These models can be helpful to understand the time profiles of analog:dNTP ratio changes in different clinical trial designs and to calculate the operational multiple dosing intracellular half-lives. Future study is required to investigate the possible mechanisms of these findings and to test other covariates.

## Supporting Information

S1 File Equations A-D: Supplement equations. **Table A: Other model evaluation results.** Obs: observed values. **Figure A: Dependent variables (observed values) and predicted values vs time plots.** IC: intracellular. PRED: predicted values. DV: dependent variables. **Figure B: Basic goodness of fit plots of TFV plasma model.** Red line represents average values. Black line represents theoretical values. Data from the same individual are shown in blue circles and are connected by lines. |iWRES|: absolute values of individual weighted residuals. **Figure C: Basic goodness of fit plots of FTC plasma model.** Red line represents average values. Black line represents theoretical values. Data from the same individual are shown in blue circles and are connected by lines. |iWRES|: absolute values of individual weighted residuals. **Figure D: Basic goodness of fit plots of intracellular TFV-DP model.** Red line represents average values. Black line represents theoretical values. Data from the same individual are shown in blue circles and are connected by lines. |iWRES|: absolute values of individual weighted residuals. **Figure E: Basic goodness of fit plots of intracellular FTC-TP model.** Red line represents average values. Black line represents theoretical values. Data from the same individual are shown in blue circles and are connected by lines. |iWRES|: absolute values of individual weighted residuals. **Figure F: Basic goodness of fit plots of intracellular dATP model.** Red line represents average values. Black line represents theoretical values. Data from the same individual are shown in blue circles and are connected by lines. |iWRES|: absolute values of individual weighted residuals. **Figure G: Basic goodness of fit plots of intracellular dCTP model.** Red line represents average values. Black line represents theoretical values. Data from the same individual are shown in blue circles and are connected by lines. |iWRES|: absolute values of individual weighted residuals. **Figure H: Basic goodness of fit plots of intracellular dGTP model.** Red line represents average values. Black line represents theoretical values. Data from the same individual are shown in blue circles and are connected by lines. |iWRES|: absolute values of individual weighted residuals. **Figure I: Basic goodness of fit plots of intracellular TTP model.** Red line represents average values. Black line represents theoretical values. Data from the same individual are shown in blue circles and are connected by lines. |iWRES|: absolute values of individual weighted residuals. **Figure J: An example of the plasma TFV/FTC vs the intracellular TFV-DP/FTC-TP “handshape” plot.** Arrows indicate the progression of time after treatment initiation. **NONMEM Code.**
(PDF)

S2 File Study Protocol. (PDF)

S3 File TREND Statement Checklist. (PDF)
