# Supplementary material for: Model Linking Plasma and Intracellular Tenofovir/Emtricitabine with Deoxynucleoside Triphosphates
Source: PLoS One. 2016 Nov 10;11(11):e0165505. doi: 10.1371/journal.pone.0165505 (PMC5104339; doi:10.1371/journal.pone.0165505)
Supplement: S1 File — Table A: Other model evaluation results. Obs: observed values. Figure A: Dependent variables (observed values) and predicted values vs time plots. IC: intracellular. PRED: predicted values. DV: dependent variables. Figure B: Basic goodness of fit plots of TFV plasma model. Red line represents average values. Black line represents theoretical values. Data from the same individual are shown in blue circles and are connected by lines. |iWRES|: absolute values of individual weighted residuals. Figure C: Basic goodness of fit plots of FTC plasma model. Red line represents average values. Black line represents theoretical values. Data from the same individual are shown in blue circles and are connected by lines. |iWRES|: absolute values of individual weighted residuals. Figure D: Basic goodness of fit plots of intracellular TFV-DP model. Red line represents average values. Black line represents theoretical values. Data from the same individual are shown in blue circles and are connected by lines. |iWRES|: absolute values of individual weighted residuals. Figure E: Basic goodness of fit plots of intracellular FTC-TP model. Red line represents average values. Black line represents theoretical values. Data from the same individual are shown in blue circles and are connected by lines. |iWRES|: absolute values of individual weighted residuals. Figure F: Basic goodness of fit plots of intracellular dATP model. Red line represents average values. Black line represents theoretical values. Data from the same individual are shown in blue circles and are connected by lines. |iWRES|: absolute values of individual weighted residuals. Figure G: Basic goodness of fit plots of intracellular dCTP model. Red line represents average values. Black line represents theoretical values. Data from the same individual are shown in blue circles and are connected by lines. |iWRES|: absolute values of individual weighted residuals. Figure H: Basic goodness of fit plots of intracellular dGTP model. Re [file pone.0165505.s001.pdf]

Supplements

Supplement equations:

gut:
$$\frac{dA(1)}{dt} = -K_a \times A(1) \tag{A}$$

central:
$$\frac{dA(2)}{dt} = K_a \times A(1) - \frac{CL}{Vc} \times A(2) - \frac{Q}{Vc} \times A(2) + \frac{Q}{Vp} \times A(3) \tag{B}$$

peripheral:
$$\frac{dA(3)}{dt} = \frac{Q}{Vc} \times A(2) - \frac{Q}{Vp} \times A(3) \tag{C}$$

dGTP:
$$\frac{dA(7)}{dt} = K_{in}^{0} \times \left(1 - \frac{1}{1 + Time^{\gamma}} \times \frac{A(4)}{A(4) + EC_{50}}\right) - A(7) \times K_{out} \tag{D}$$

	Model Output Condition Number	Bootstrapping Success Rate	VPC Obs outside 90% CI
TFV plasma	25.3	99.2%	7.59%
FTC plasma	79.6	94.3%	7.28%
TFV-DP IC	24.9	99.3%	1.81%
FTC-TP IC	27.8	99.5%	5.00%
dATP	6.0	95.7%	9.89%
dCTP	5.5	99.7%	10.4%
dGTP	4.4	99.2%	12.0%
TTP	7.5	99.9%	11.6%

Table A. Other model evaluation results. Obs: observed values.

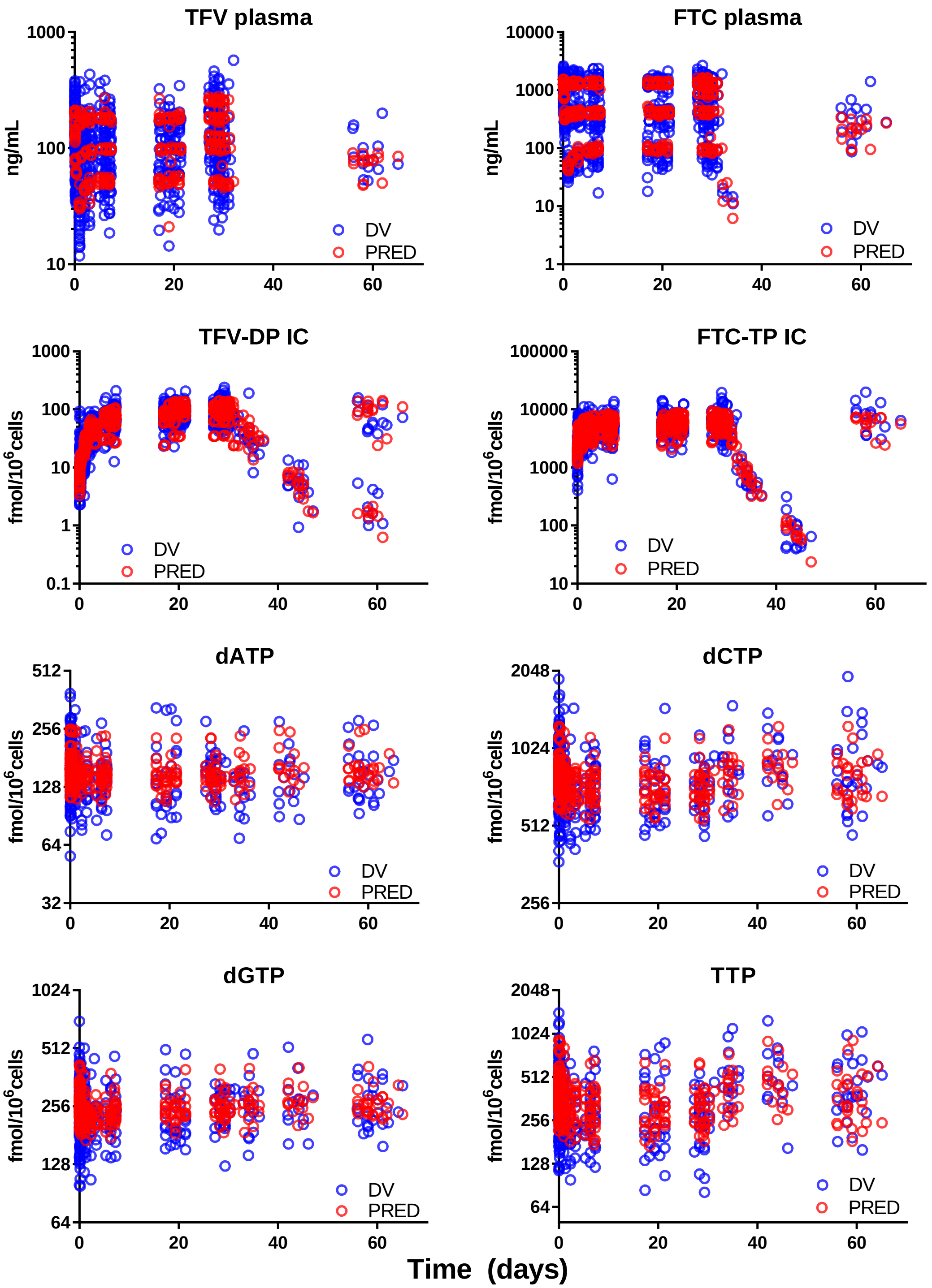

Figure A. Dependent variables (observed values) and predicted values vs time plots. IC: intracellular. PRED: predicted values. DV: dependent variables.

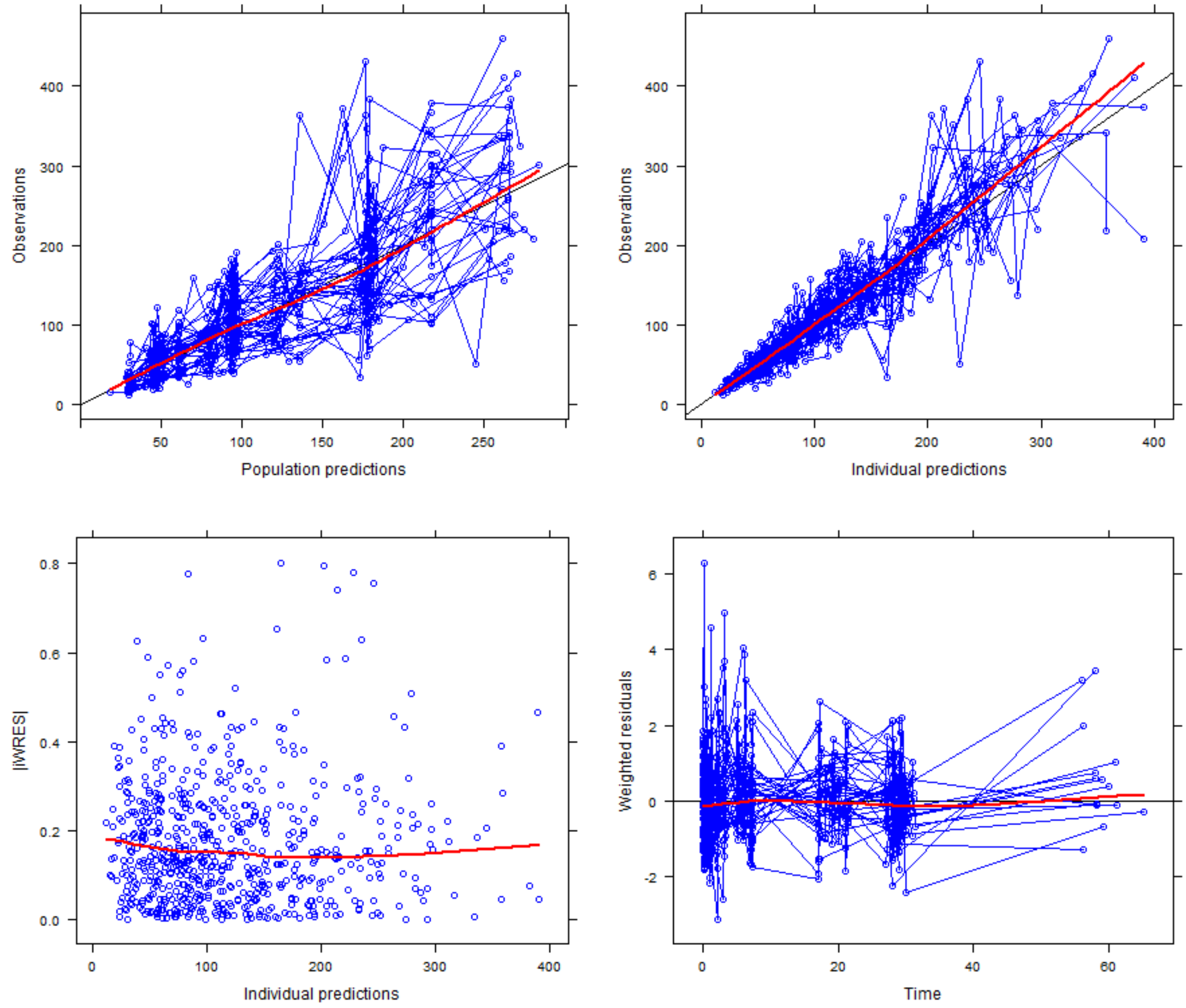

Figure B. Basic goodness of fit plots of TFV plasma model. Red line represents average values. Black line represents theoretical values. Data from the same individual are showed in blue circles and are connected by lines. |iWRES|: absolute values of individual weighted residuals.

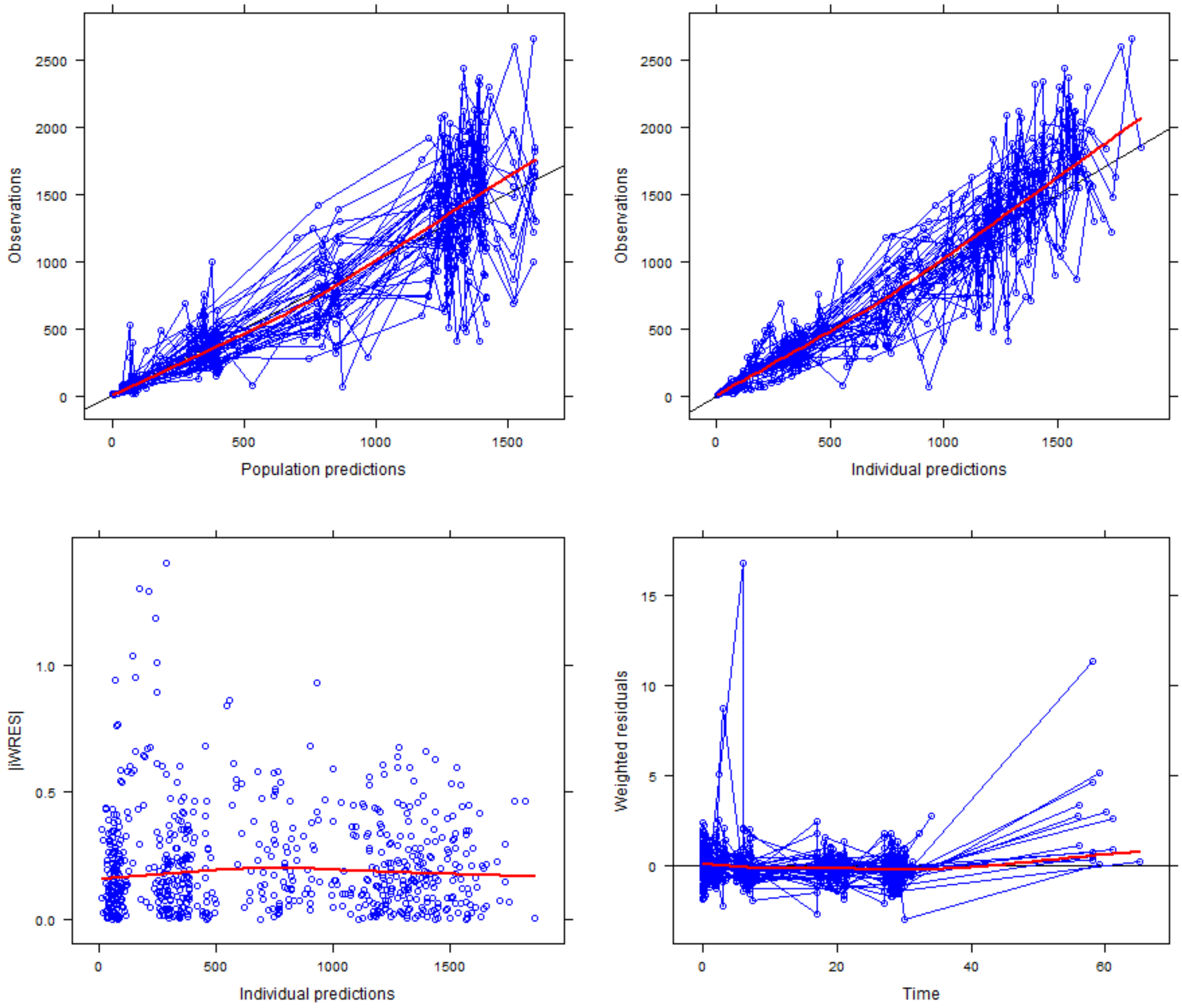

Figure C. Basic goodness of fit plots of FTC plasma model. Red line represents average values. Black line represents theoretical values. Data from the same individual are showed in blue circles and are connected by lines. |iWRES|: absolute values of individual weighted residuals.

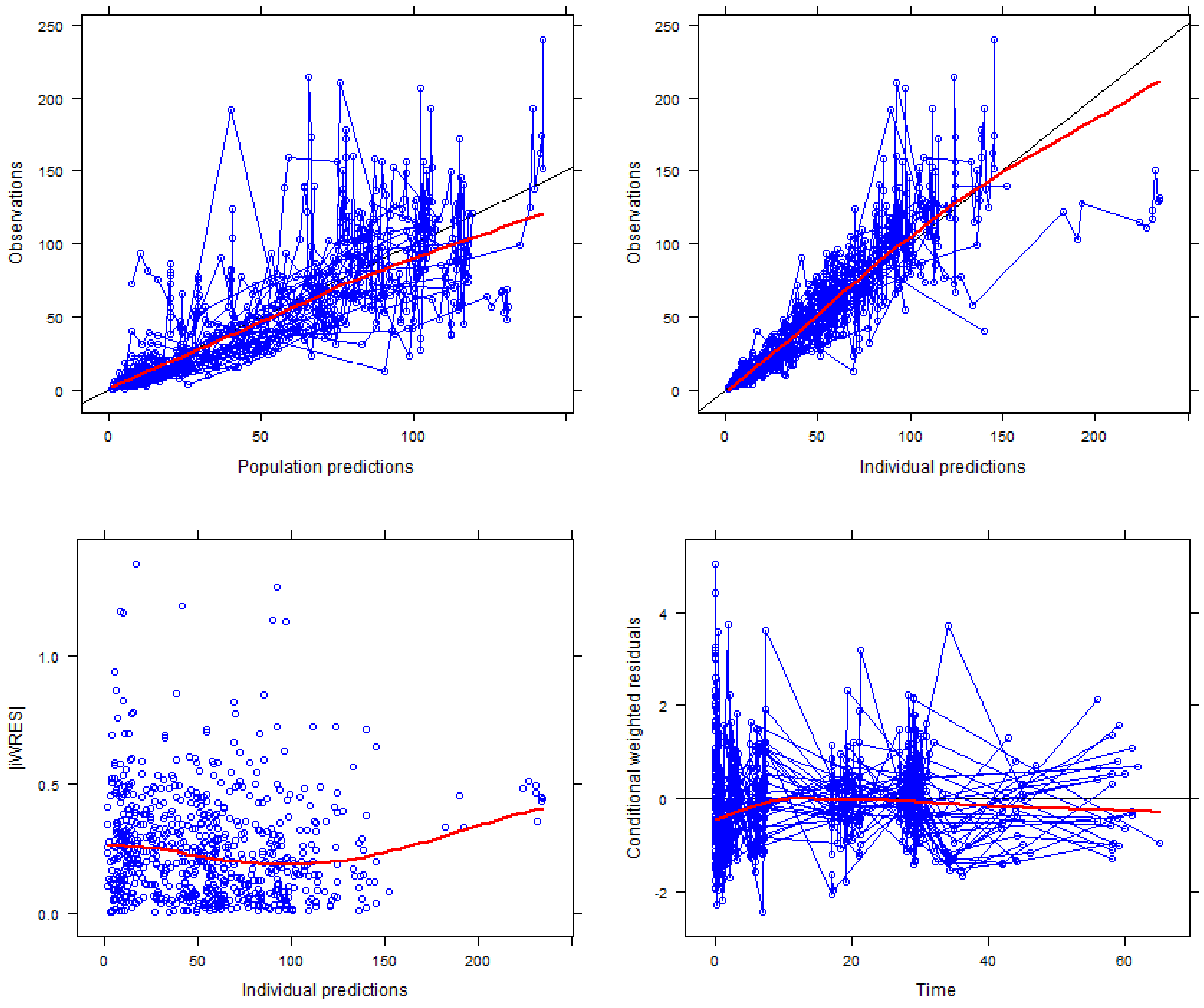

Figure D. Basic goodness of fit plots of intracellular TFV-DP model. Red line represents average values. Black line represents theoretical values. Data from the same individual are showed in blue circles and are connected by lines. |iWRES|: absolute values of individual weighted residuals.

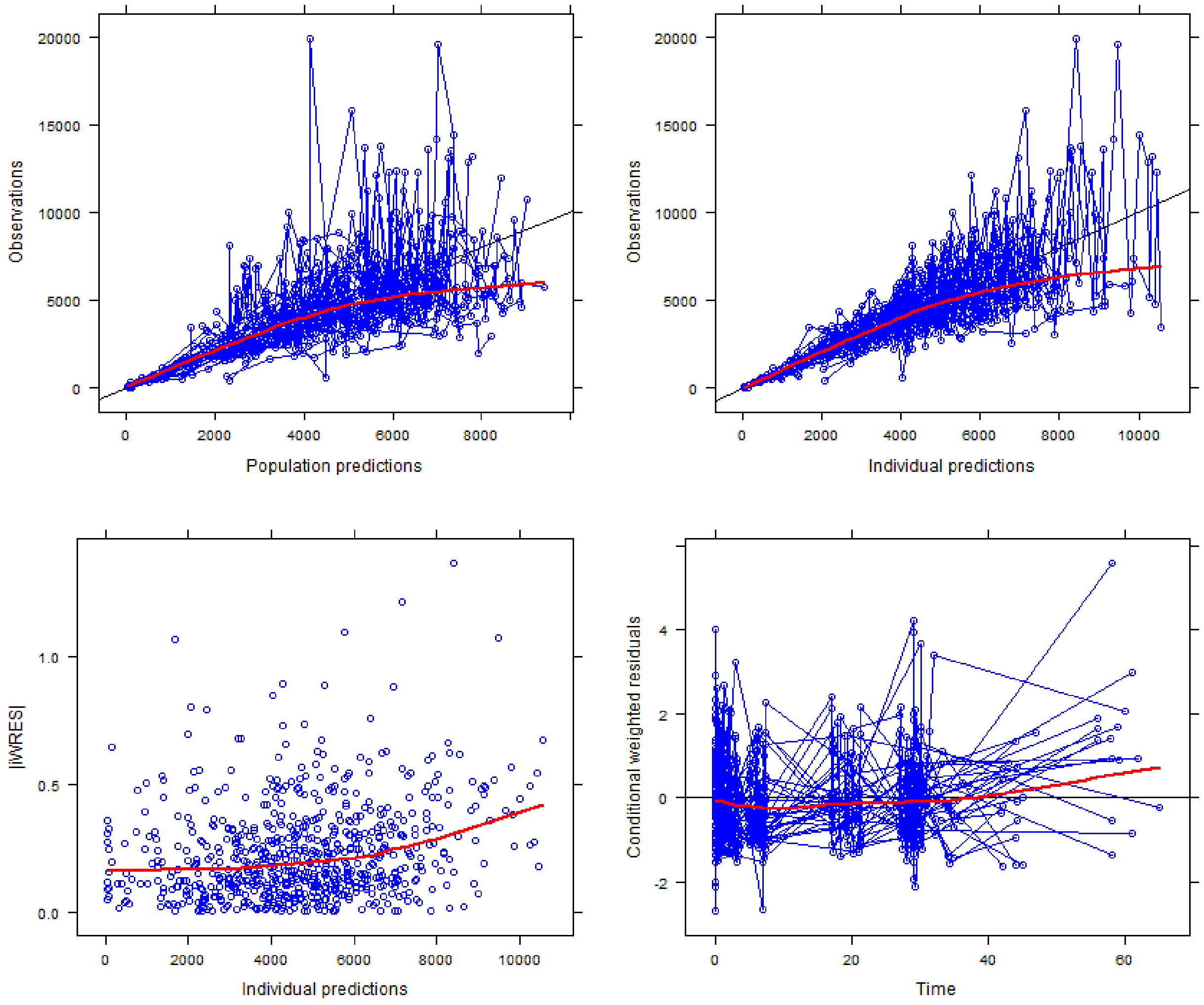

Figure E. Basic goodness of fit plots of intracellular FTC-TP model. Red line represents average values. Black line represents theoretical values. Data from the same individual are showed in blue circles and are connected by lines. |iWRES|: absolute values of individual weighted residuals.

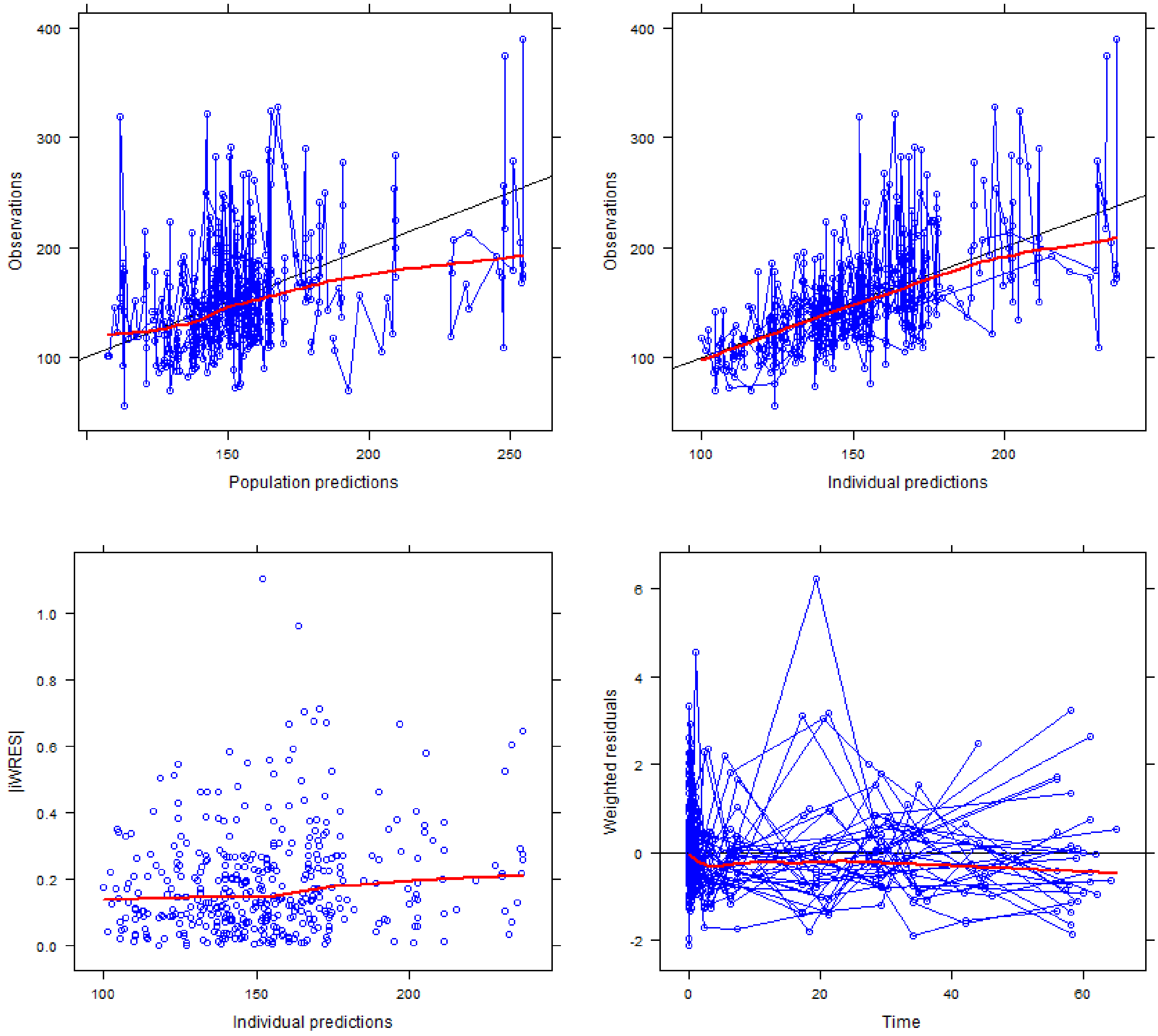

Figure F. Basic goodness of fit plots of intracellular dATP model. Red line represents average values. Black line represents theoretical values. Data from the same individual are showed in blue circles and are connected by lines. |iWRES|: absolute values of individual weighted residuals.

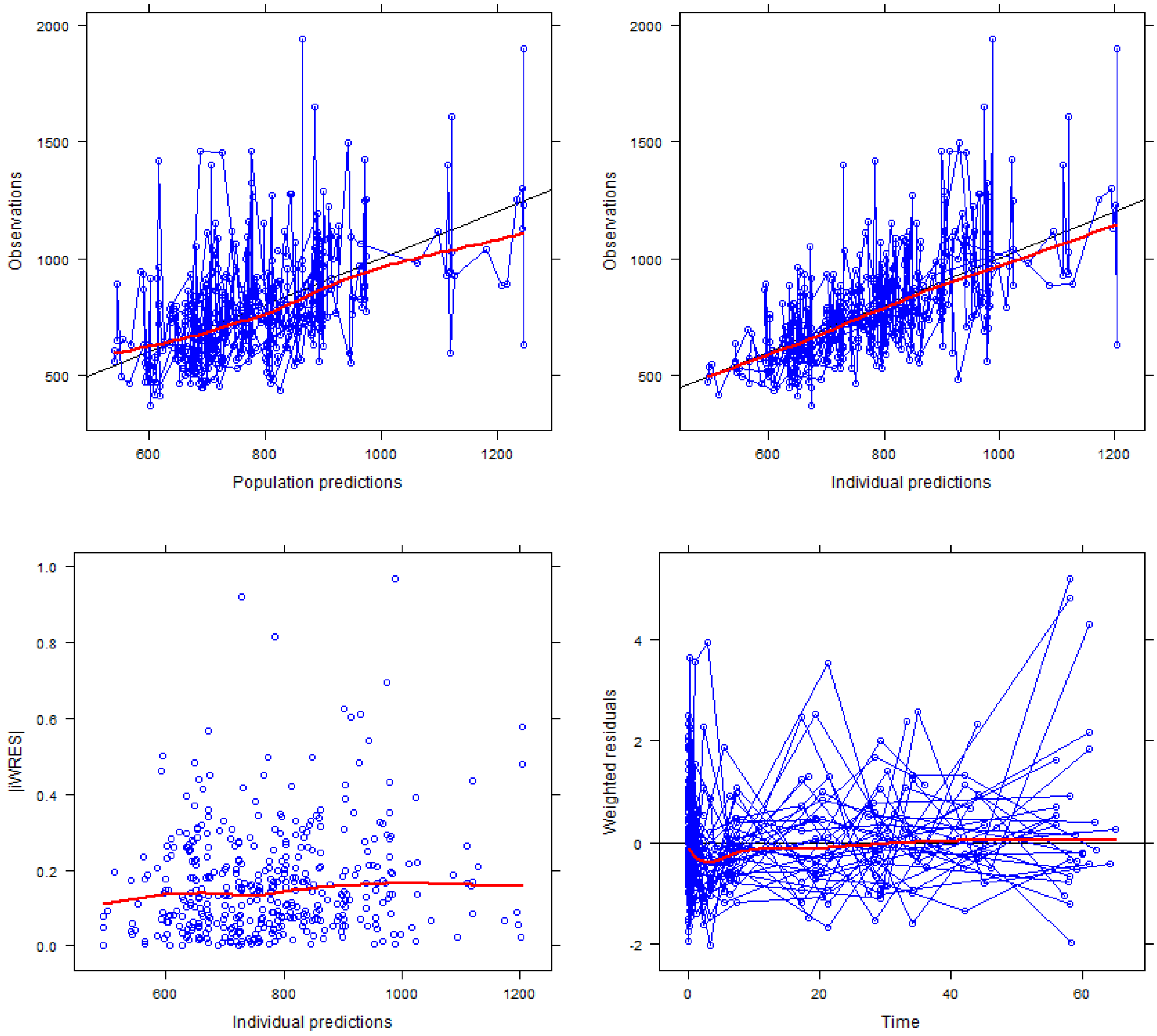

Figure G. Basic goodness of fit plots of intracellular dCTP model. Red line represents average values. Black line represents theoretical values. Data from the same individual are showed in blue circles and are connected by lines. |iWRES|: absolute values of individual weighted residuals.

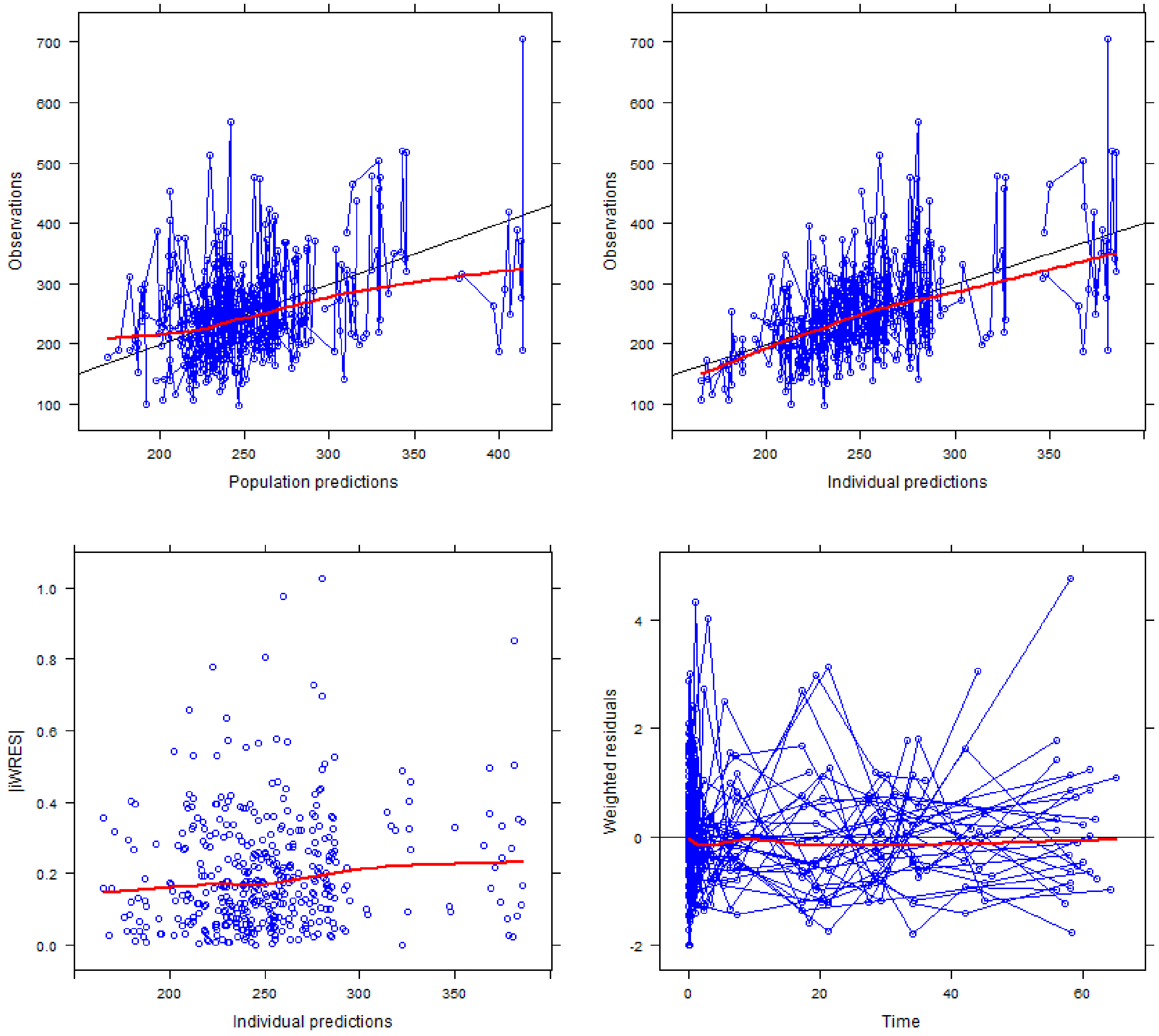

Figure H. Basic goodness of fit plots of intracellular dGTP model. Red line represents average values. Black line represents theoretical values. Data from the same individual are showed in blue circles and are connected by lines. |iWRES|: absolute values of individual weighted residuals.

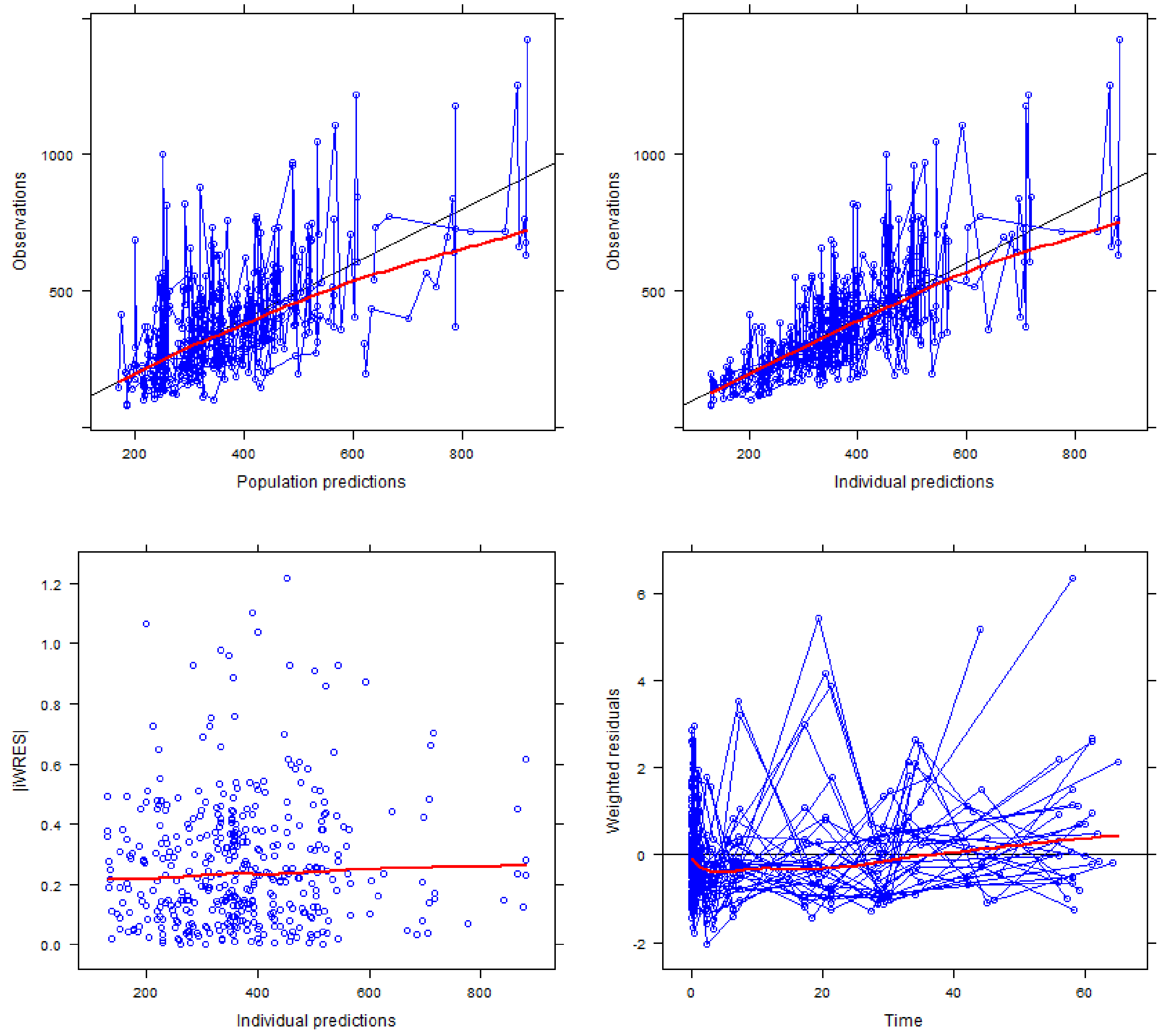

Figure I. Basic goodness of fit plots of intracellular TTP model. Red line represents average values. Black line represents theoretical values. Data from the same individual are showed in blue circles and are connected by lines. |iWRES|: absolute values of individual weighted residuals.

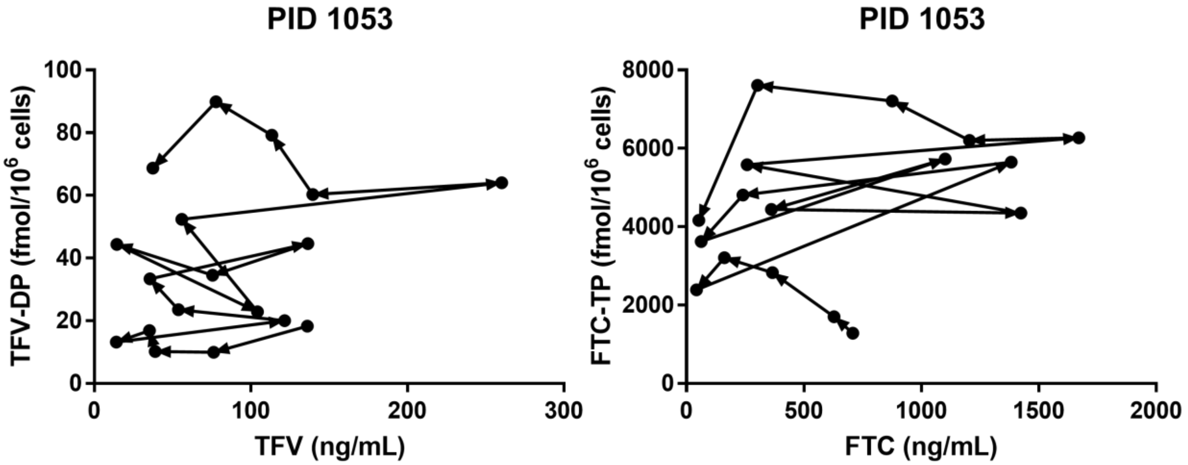

Figure J. An example of the plasma TFV/FTC vs the intracellular TFV-DP/FTC-TP "handshape" plot. Arrows indicate the progression of time after treatment initiation.

NONMEM code:

The NONMEM code for the modeling of concentration-time data are selectively shown below.

Plasma model:

```
$SUBROUTINES ADVAN4 TRANS4

$PK

   TVCL=THETA(1)
   CL=TVCL*EXP(ETA(1))
   TVV2=THETA(2)
   V2=TVV2*EXP(ETA(2))
   TVQ=THETA(3)
   Q=TVQ*EXP(ETA(3))
   TVV3=THETA(4)
   V3=TVV3*EXP(ETA(4))
   TVKA=THETA(5)
   KA=TVKA;
   S2=V2
```

Intracellular PK link model:

```
$SUBROUTINES ADVAN13 TRANS1 TOL=6

$MODEL

   NCOMP=6
   COMP = (DEPOT, DEFDOS)
   COMP = (CENTRAL)
   COMP = (RESPONSE, DEFOBS)
   COMP = (PHERIPH)
   COMP = (SATURATION)
   COMP = (RECYCLE)

$PK

   CL2  = ICL
   V2   = IV2
   KA2  = IKA
   Q    = IQ
   V4   = IV3
   SIN  = THETA(1)
   SOUT = CL2/V2
   KF   = THETA(1)*EXP(ETA(1))
   SC50 = THETA(2)
   KEL  = THETA(3)*EXP(ETA(2))
   R    = THETA(4)
   S2   = V2
   K20  = CL2/V2
   K24  = Q/V2
   K42  = Q/V4

$DES
```

```
DADT(1) = -KA2*A(1)
DADT(2) = KA2*A(1) - K20*A(2) + K42*A(4) - K24*A(2)
C2 = A(2)/V2
DADT(5) = SIN*C2-A(5)*SOUT
CS=A(5)
KR=KEL*R/100
DADT(3) = KF*C2/(1+CS/SC50)-KEL*A(3)+KR*A(6)
DADT(4) = -K42*A(4) + K24*A(2)
DADT(6) = KR*A(3) - KR*A(6)
```

Intracellular PKPD link model:

```
$SUBROUTINES ADVAN13 TRANS1 TOL=6

$MODEL

   NCOMP=7
   COMP = (DEPOT, DEFDOS)
   COMP = (CENTRAL)
   COMP = (RESPONSE)
   COMP = (PHERIPH)
   COMP = (SATURATION)
   COMP = (RECYCLE)
   COMP = (DNTP, DEFOBS)

$PK

   CL2  = ICL
   V2   = IV2
   KA2  = IKA
   Q    = IQ
   V4   = IV3
   SIN  = ISIN
   SOUT = CL2/V2
   KIN  = IKIN
   SC50 = ISC50
   KOUT = IKOUT
   R    = IR
   S2   = V2
   K20  = CL2/V2
   K24  = Q/V2
   K42  = Q/V4

   BPOP   = THETA(1)

   IIV    = THETA(2)
   RV     = THETA(3)
   BIDV   = BPOP*EXP(ETA(2)*IIV)
   IIVW   = IIV**2/(IIV**2+RV**2)
   RVW    = RV**2/(IIV**2+RV**2)
   BRV    = EXP(ETA(1)*RV*IIVW)
   IBASE  = (BPOP*RVW + OBASE*IIVW)*BRV
   KOUT   = 1
   KIN    = IBASE*THETA(5)
   A_0(7) = KIN/KOUT
```

```
   EC50   = EXP(THETA(4))*EXP(ETA(3))

$DES

   DADT(1) = -KA2*A(1)
   DADT(2) = KA2*A(1) - K20*A(2) + K42*A(4) - K24*A(2)
   C2 = A(2)/V2
   DADT(5) = SIN*C2-A(5)*SOUT
   CS=A(5)
   KR=KEL*R/100
   DADT(3) = KF*C2/(1+CS/SC50)-KEL*A(3)+KR*A(6)
   DADT(4) = -K42*A(4) + K24*A(2)
   DADT(6) = KR*A(3) - KR*A(6)
   DADT(7) = KIN * ( 1-  A(3) / ( EC50 +  A(3) ) ) - KOUT*A(7)

$ERROR

 IF (TIME.EQ.0) THEN
 IPRE=IBASE
 ELSE
 IPRE=A(7)
 ENDIF

 Y = IPRE*EXP(EPS(1)*RV)

$OMEGA
  1 FIXED ;[P] omega(1,1)
  1 FIXED ;[P] omega(2,2)
  ……      ;[P] omega(3,3)

$SIGMA
  1 FIXED ;[P] sigma(1,1)
```
