# Supplementary material for: Model Linking Plasma and Intracellular Tenofovir/Emtricitabine with Deoxynucleoside Triphosphates
Source: PLoS One. 2016 Nov 10;11(11):e0165505. doi: 10.1371/journal.pone.0165505 (PMC5104339; doi:10.1371/journal.pone.0165505)
Supplement: S2 File — (PDF) [file pone.0165505.s002.pdf]

PROTOCOL:

Cellular Pharmacology of Tenofovir and Emtricitabine for HIV Prophylaxis
"Cell Prep"

LOCAL IDENTIFIERS:
Colorado Multiple Institutional Review Board: 08-0459
Clinical Translational Research Center: 1812

APPROVED
JAN 29 2013
COMIRB

NIAID FUNDING SUPPORT:
DAIDS, NIAID, NIH
U01 AI84735

DAIDS-ES #10817

PHARMACEUTICAL SUPPORT:
Gilead Sciences

IND SPONSOR:
DAIDS, NIAID, NIH
IND #71,859

PRINCIPAL INVESTIGATOR
Peter L. Anderson, Pharm.D.

Version 2.0
03 January 2013

SIGNATURE PAGE:

The signature below constitutes the approval of this protocol and the attachments, and provides the necessary assurances that this trial will be conducted according to all the stipulations of the protocol, including all statements regarding confidentiality, and according to local legal and regulatory requirements and applicable U.S. Federal regulations and International Conference on Harmonisation guidelines.

____
Peter L. Anderson, Pharm.D.
Associate Professor of Pharmaceutical Sciences
Principal Investigator

____
Date

LIST OF ACRONYMS

AE	Adverse Event
ALCOA	Attributable, Legible, Contemporaneous, Original, and Accurate
AUC	Area Under the Concentration-time Curve
B Cell	Bone Marrow-derived lymphocyte Cell
BCRP	Breast Cancer Resistance Protein
CAVP	Colorado Antiviral Pharmacology Laboratory
CBC	Complete Blood Count
CD	Cluster Designation
CL	Clearance
CLIA	Clinical Laboratory Improvement Amendments
CMP	Comprehensive Metabolic Panel
CNS	Central Nervous System
COMIRB	Colorado Multiple Institutional Review Board
CRFs	Case Report Forms
CTRC	Clinical Translational Research Center
DAIDS	Division of Acquired Immunodeficiency Syndrome
ddI	Didanosine
DNA	Deoxyribonucleic acid
DP	Diphosphate
DSMB	Data Safety Monitoring Board
EFV	Efavirenz
FDA	Food and Drug Administration
FTC	Emtricitabine
FTC-MP	Emtricitabine-monophosphate
FTC-DP	Emtricitabine-diphosphate
FTC-TP	Emtricitabine-triphosphate
GCP	Good Clinical Practices
GRF	Glomerular Filtration Rate
HBV	Hepatitis B Virus
HIPAA	Health Insurance Portability and Accountability Act
HIV	Human Immunodeficiency Virus
HLA-DR	Human Leukocyte Antigen Type DR
HRRC	Hospital Research Review Committee
IFN-α	Human Type I Interferon Alpha
iPrEx	"Chemoprophylaxis for HIV infection in men" study
IRB	Institutional Review Board
IUD	Intrauterine Device
LFTs	Liver Function Tests
LOB	Leprino Office Building
MCs	Mononuclear Cells
MDRD	Modification of Diet in Renal Disease
MP	Monophosphate
MRP	Multidrug Resistance-Associated Protein
MSM	Men Who have Sex With Men
NIAID	National Institute of Allergy and Infectious Diseases
NIH	National Institutes of Health
NONMEM	Nonlinear Mixed-Effect Modeling
NRTIs	Nucleoside analog Reverse Transcriptase Inhibitors
NRTI-TP	Nucleoside analog Reverse Transcriptase Inhibitor-Triphosphate

NSAIDs	Non-steroidal Anti-inflammatory Drugs
NVP	Nevirapine
OHRP	Office for Human Research Protections
PBMCs	Peripheral Blood Mononuclear Cells
PHA	Phytohemagglutinin
PK	Pharmacokinetics
PNP	Purine Nucleoside Phosphorylase
QA/QC	Quality Assurance/Quality Control
RSA	Research Subject Advocate
SD	Standard Deviation
SOP	Standard Operating Procedure
sTNFrII	soluble Tumor Necrosis Factor receptor II
T Cell	Thymus-matured lymphocyte Cell
TB	Tuberculosis
TDF	Tenofovir Disoproxil Fumarate
TDF/FTC/EFV	Tenofovir Disoproxil Fumarate/Emtricitabine/ Efavirenz
TDF/FTC	Truvada® or Tenofovir Disoproxil Fumarate/Emtricitabine
TFV	Tenofovir
TFV-DP	Tenofovir-diphosphate
TFV-MP	Tenofovir-monophosphate
TNF-α	Tumor Necrosis Factor Alpha
TP	Triphosphate
UA	Urinalysis

TABLE OF CONTENTS

Protocol Summary.......8

1. KEY ROLES 11
 1.1 Personnel.......11
 1.2 Clinical Laboratories, Medical Departments, Institutions, Pharmacy 15

2. INTRODUCTION 17
 2.1 Background Information.......17
  2.1.1 Pharmacologic effects for TDF and FTC depend upon their cellular pharmacology ... 17
  2.1.2 Cellular pharmacology cannot be directly extrapolated from in vitro to in vivo setting 17
  2.1.3 Important reasons to directly study the cellular pharmacology NRTIs in vivo 18
  2.1.4 TDF and FTC in a new in vivo setting: HIV prophylaxis 18
  2.1.5 A summary of the high significance and rationale for this specific research 19
 2.2 Hypothesis and Specific Aims 20
 2.3 Potential Risks and Benefits 20
  2.3.1 Study medication risks, HIV-positive persons.......21
  2.3.2 Study medication risks, HIV-negative persons.......21
   2.3.2.1 Approaches to minimize drug risks 22
   2.3.2.2 Summary assessment of drug risks....... 23
  2.3.3 Rectal mucosal biopsy risks....... 23
  2.3.4 Cervical brush sampling 23
  2.3.5 Semen sample 24
  2.3.6 Blood collections 24

3. OBJECTIVES.......24
 3.1 Primary Objectives 24
 3.2 Secondary Objectives 25

4. STUDY DESIGN 26

5. STUDY POPULATION 28
 5.1 Selection of the Study Population.......28
 5.2 Inclusion/Exclusion Criteria 28
  5.2.1 Inclusion criteria for HIV-uninfected cohort.......28
  5.2.2 Exclusion criteria for HIV-uninfected cohort.......28
  5.2.3 Inclusion criteria for HIV-infected cohort 29
  5.2.4 Exclusion criteria for HIV-infected cohort.......30

6. STUDY PRODUCT INTERVENTION(S) 30
 6.1 Regimen.......30
 6.2 Study Product Formulation and Preparation.......31
 6.3 Study Product Supply and Accountability.......31
 6.4 Assessment of Participant Adherence with Study Product/Intervention 31
 6.5 Concomitant Medications and Procedures.......32
 6.6 Permitted Medications and Procedures.......32
 6.7 Prohibited Medications and Procedures.......32
 6.8 Precautionary Medications and Procedures.......32
 6.9 Required Medications and Procedures.......32

6.10 Rescue Medications and Procedures 33

7. STUDY PROCEDURES/EVALUATIONS 33
7.1 Clinical Evaluations and Procedures 33
7.1.1 Medical History and Physical Exam 36
7.1.2 Mouthwash Sampling for DNA 36
7.1.3 Rectal mucosal biopsy 36
7.1.4 Cervical brush sampling 36
7.1.5 Semen sample 37
7.1.6 Blood collections 37
7.1.7 Sexually Transmitted Infection and Vaginitis Testing 37
7.1.8 Urine Testing 37
7.1.9 Medication Calendar, Pill Count, Adverse Events Questionnaire 37
7.2 Laboratory Evaluations 38
7.2.1 Laboratory Evaluations and Specimen Collection 38
7.2.2 Specimen Preparation, Handling and Shipping 38
7.3 Biohazard Containment 39
7.4 Sequencing of Procedures/Evaluations: Timing and Definitions 39

8. ASSESSMENT OF SAFETY 39
8.1 Adverse Event Definition 39
8.2 Adverse Events Procedures and Requirements for Expedited Reporting 40
8.3 Expedited Adverse Events 41
8.4 Local Regulatory Requirements 42

9. CLINICAL MANAGEMENT 42
9.1 Toxicity Management 42
9.2 Other Disease Events 43
9.3 Pregnancy 43
9.4 Breastfeeding (if applicable) 44
9.5 Criteria for Study Discontinuation 44
9.6 Criteria for Permanent Treatment Discontinuation for an Individual Participant 44
9.7 Criteria for Premature Study Discontinuation for an Individual Participant 45

10. STATISTICAL CONSIDERATIONS 45
10.1 Overview and design issues 45
10.2 Study endpoints 48
10.3 Sample Size Considerations 48
10.4 Enrollment/Stratification/Randomization/Blinding Procedures 49
10.5 Participant Enrollment Follow-Up 49
10.6 Data and Safety Monitoring 50
10.7 Planned Interim Analyses and Stopping Guidelines 50
10.8 Safety Review 50
10.9 Analysis Plan 50

11. DATA HANDLING AND RECORDKEEPING 50
11.1 Data Management Responsibilities 50
11.2 Source Documents and Access to Source Data/Documents 51
11.3 Quality Control and Quality Assurance 51

12. CLINICAL SITE MONITORING 51

12.1 External Auditing 51
12.2 Site Visit Summary Meeting 52
12.3 The Site Monitoring Report 52
12.4 Review and Follow up of Monitoring Report Findings 52
13. HUMAN SUBJECTS PROTECTION 52
13.1 Institutional Review Board/Ethics Committee 52
13.2 Protocol Registration 53
13.3 Informed Consent Process 53
13.4 Participant Confidentiality 53
13.5 Study Discontinuation 54
14. PUBLICATION POLICY 54
15. REFERENCES 55
16. APPENDICES 58
Appendix A: Schedule of Events 58
Appendix B: Safety officer charter 58
Appendix C: Informed Consents 58

PROTOCOL SUMMARY (DAIDS-ES #10817):

Full Title: Cellular pharmacology of tenofovir and emtricitabine for HIV prophylaxis

Short Title: "Cell Prep"

Clinical Phase: I-II

IND Sponsor: DAIDS, NIAID, NIH (IND #71,859)

Principal Investigator: Peter L. Anderson, Pharm.D.

Sample Size: 40 who complete the study. Colorado Multiple Institutional Review Board (COMIRB) approval will be sought for 60 subjects to allow for screen failures.

Study population: Two equal sized groups of adult volunteers, 20 HIV-infected and 20 HIV-uninfected participants who complete the study.

Participating sites: The study will be conducted at a single site, the University of Colorado Denver Clinical Translational Research Center (CTRC).

Study Design: The overarching hypothesis for this project is that the optimal use of tenofovir and emtricitabine for HIV prevention and treatment is achieved through rational dosing strategies founded on their cellular pharmacology profile in patients. This study is a phase I-II open label, prospective, observational intensive pharmacokinetic analysis of intracellular tenofovir and emtricitabine in 20 HIV-negative and 20 HIV-positive human adult volunteers. The cellular pharmacology of tenofovir-diphosphate (TFV –DP) and emtricitabine-triphosphate (FTC-TP) will be intensively characterized and compared according to HIV disease status. Purine concentrations to assess purine nucleoside phosphorylase (PNP) inhibition will be determined in urine, plasma, and cells before therapy and during steady-state Truvada® (TDF/FTC). The two serostatus cohorts will be enrolled to target 50% women and 50% African Americans when possible because secondary analyses will assess gender and race effects on TFV/FTC cellular pharmacology.

Stored samples will also be analyzed retrospectively from an ongoing multi-center, randomized, double-blind, placebo-controlled, clinical trial of tenofovir/emtricitabine for the prevention of HIV in MSM called "Chemoprophylaxis for HIV prevention in men", or iPrEx. This study is open for enrollment in Peru, Ecuador, Brazil, South Africa, the United States and Thailand. Stored samples from this study will be analyzed at the University of Colorado as part of the present study to assess a prophylactic threshold for intracellular tenofovir and emtricitabine.

Study Duration: The study consists of approximately 9 pharmacokinetic visits over 60 days for each participant. There are no additional follow up visits.

Recruitment and enrollment will continue for three years, the total study duration is four years.

Study Regimen: HIV negative volunteers receive 200mg emtricitabine and 300mg of tenofovir disoproxil fumarate (as Truvada®) once daily for 30 days. This medication will be supplied by the study. HIV-positive volunteers receive 200mg emtricitabine, 300mg of tenofovir disoproxil fumarate, and 600mg efavirenz by prescription from their physicians. The study does not provide medications to the HIV-positive volunteers nor does it affect their usual clinical care for HIV.

Primary Objectives: The study has four main objectives:

1. To characterize and compare the profiles of intracellular TFV-diphosphate (TFV-DP) and FTC-triphosphate (FTC-TP) in HIV-negative versus HIV-positive adults.

2. To evaluate whether the pharmacologic spectrum of TFV includes inhibition of purine-nucleoside-phosphorylase in vivo.

3. To identify a prophylactic threshold for intracellular TFV -DP and FTC-TP in peripheral blood mononuclear cells (PBMCs) from subjects from the iPrEx study.

4. To develop a comprehensive pharmacokinetic model for TFV -DP and FTC-TP according to cell-type and tissue compartment with which to predict the optimal dose and onset and duration of prophylactic action.

Primary Endpoints: The primary endpoints are:

1. A comparison of the first dose TFV-DP and FTC-TP AUC in HIV-negative versus HIV-positive adults.

2. A comparison of average steady-state plasma deoxyguanosine concentrations before therapy and after 30 days of TDF/FTC therapy.

3. A comparison of TFV-DP and FTC-TP in HIV-seroconverters versus matched non-seroconverters from the iPrEx study.

4. A pharmacokinetic model that describes the intracellular pharmacokinetics of TFV-DP and FTC-TP in PBMCs so that various dosing strategies can be tested on the model to identify the optimal dosing that most rapidly achieves and sustains the desired prophylactic threshold for HIV prevention.

Secondary Outcomes:

1. A definition of the terminal elimination phase of TFV-DP and FTC-TP in HIV-negative adults.

2. A characterization of TFV-DP and FTC-TP according to the following cell types: PBMCs, CD4-purified PBMCs (as well as erythrocytes-to include dried blood spot analyses, CD8 cells, B-cells, and monocytes), genital mononuclear cells, and rectal mucosal mononuclear cells.

3. A comparison of TFV-DP and FTC-TP between men and women.

4. A comparison of TFV-DP and FTC-TP between African-Americans and non-African-Americans.

5. A characterization of intracellular TFV, TFV-MP, FTC-MP, and FTC-DP.

6. An evaluation of polymorphisms in MRP2 (e.g. -24C>T and 1249G>A), MRP4 (e.g. 1612C>T, 3463G>A, 3724G>A, and 4131T>G), BCRP (e.g. 421C>A and 34G>A) and other potentially important enzymes for the study drugs for relationships with pharmacokinetics and pharmacodynamics.

7. An evaluation of markers of cell activation (HLA-DR and CD38 expression) and the relationship to TFV-DP and FTC-TP concentrations.

8. The day 30 AUC and overall AUC (AUC over day 1 to day 30) TFV-DP and FTC-TP will be compared in HIV-negative versus HIV-positive subjects.

9. HLA-DR / CD38 on T cells will be correlated with changed intracellular and extracellular purine levels in the HIV-negative volunteers to address potential immune-modulation associated with PNP inhibition.

10. The ratios of TFV-DP and FTC-TP to corresponding endogenous deoxyribose nucleotides will be characterized.

11. The effects of TFV-DP and FTC-TP (and plasma EFV) on plasma HIV-RNA and CD4 counts will be determined in the HIV-positive cohort.

12. The TFV-DP and FTC-TP will be compared according to iPrEx study sites.

13. Relationships between adherence measures collected in iPrEx with TFV-DP and FTC-TP will be evaluated.

1. KEY ROLES

1.1 Personnel

Principal Investigator:
Peter L. Anderson, Pharm.D.
Associate Professor of Pharmaceutical Sciences
University of Colorado Denver
School of Pharmacy
Pharmacy and Pharmaceutical Sciences Building, C238-V20
12850 E. Montview Blvd. Room V20-4101
Aurora, CO 80045
303-724-6128
peter.anderson@ucdenver.edu

NIAID Medical Officer:
David Burns, M.D.
Medical Officer
Prevention Sciences Program/Division of AIDS/NIAID/NIH
Room 5121 MSC 7628
6700 B Rockledge Dr.
Bethesda, MD 20892 USA
301-435-8896
Fax: 301-496-8530
burnsda@niaid.nih.gov

NIAID Program Officers:
Grace Chow, Biologist
Program Officer
Prevention Sciences Program/Division of AIDS/NIAID/NIH
6700B Rockledge Dr, Rm. 5119
Bethesda, MD 20892
301-435-3747
Fax: 301-496-8530
gchow@niaid.nih.gov

Hao Zhang, M.D. Pharmacologist
Program Officer, DDCSB
Therapeutics Research Program/Division of AIDS/NIAID/NIH
6700B Rockledge, Rm. 5153
Bethesda, MD 20892
301-451-2191
Fax: 301-435-9282
hazhang@niaid.nih.gov

Gilead Representative (Study drug):
Naz Barlow
Senior Clinical Research Associate
Gilead Sciences, Inc.

333 Lakeside Drive
Foster City, CA 94404
650-522-1238
Fax: 650-522-5595
naz.barlow@gilead.com

Study Physician:

Amie Meditz, M.D.
Instructor of Medicine
University of Colorado Denver
School of Medicine
Research 2, B168 - P15
12700 E. 19th Ave. Room P15- 11010
Aurora, CO 80045
303-724-4934
amie.meditz@ucdenver.edu

Study Physician Consultant:

Liz Connick, M.D.
Associate Professor of Medicine
University of Colorado Denver
School of Medicine
Research 2, B168 - P15
12700 E. 19th Ave. Room P15- 11020
Aurora, CO 80045
303-724-4930
liz.connick@ucdenver.edu

Jesus Rivera, M.D.
Associate Professor
University of Colorado Denver
School of Medicine, Division of Gastroenterology and Hepatology
Research 2, B146 - P15
12700 E. 19th Ave. Room P15- 10026
Aurora, CO 80045
303-724-7248
jesus.rivera-nieves@ucdenver.edu

Dr. Gregory L. Austin, MD, MPH
Assistant Professor
University of Colorado Denver
School of Medicine, Division of Gastroenterology and Hepatology
Academic Office One, B158-L15
E. 17th Ave. Room L15- 7609
Aurora, CO 80045
303-724-1858
gregory.austin@ucdenver.edu

Safety Officer:

Cara Wilson, M.D.
Associate Professor of Medicine

University of Colorado Denver
School of Medicine
Research 2, B168 - P15
12700 E. 19th Ave. Room P15- 11011
Aurora, CO 80045
303-724-4601
cara.wilson@ucdenver.edu

Consultants:

Robert Grant, M.D., M.P.H., M.S.
Associate Professor of Medicine
Betty Jean and Hiro Ogawa Endowed Investigator
Gladstone Institute of Virology and Immunology
University of California, San Francisco
School of Medicine
P.O. Box 1230
San Francisco, California 94143
415-734-4810
robert.grant@ucsf.edu

Study Coordinator:

Julie Predhomme, R.N., M.S., A.N.P., A.C.R.N.
Senior Professional Research Assistant
University of Colorado Denver
School of Pharmacy
Pharmacy and Pharmaceutical Sciences Building, C238-V20
12850 E. Montview Blvd. Room V20-4105
Aurora, CO 80045
303-724-1087
julie.predhomme@ucdenver.edu

Co-investigators:

Jennifer Kiser, Pharm.D.
Assistant Professor of Pharmaceutical Sciences
University of Colorado Denver
School of Pharmacy
Pharmacy and Pharmaceutical Sciences Building, C238-V20
12850 E. Montview Blvd. Room V20-4102
Aurora, CO 80045
303-724-6131
jennifer.kiser@ucdenver.edu

Edward Gardner, M.D.
Assistant Professor of Medicine
University of Colorado Denver
Denver Health Medical Center
Denver Public Health
605 Bannock Street
Denver, CO 80204
303-602-8740
edward.m.gardner@dhha.org

Kyle Hammond, Pharm.D.
Post-doctoral fellow
University of Colorado Denver
School of Pharmacy
Pharmacy and Pharmaceutical Sciences Building, C238-V20
12850 E. Montview Blvd. Room V20-4104
Aurora, CO 80045
303-724-5564
kyle.hammond@ucdenver.edu

Jose Castillo-Mancilla, M.D.
Assistant Professor
University of Colorado Denver
School of Medicine
Research 2, B168 - P15
12700 E. 19th Ave.
Aurora, CO 80045
303-724-4601
jose.castillo-mancilla@ucdenver.edu

Statisticians:
Amanda Allshouse, M.S.
Research Instructor of Public Health
University of Colorado Denver
School of Public Health
13001 E.17th Place
Box C245 Rm. W3119
Aurora, CO 80045
303-724-4350
amanda.allshouse@ucdenver.edu

Samantha MaWhinney, Sc.D.
Associate Professor of Public Health
University of Colorado Denver
School of Public Health
13001 E.17th Place
Box B119 Rm. W3127
Aurora, CO 80045
303-724-4368
samantha.mawhinney@ucdenver.edu

1.2 Clinical Laboratories, Medical Departments, Institutions, Pharmacy

Clinical Monitoring Lab:

University of Colorado Hospital
Clinical Laboratory
Mail Stop A022
12401 East 17th Avenue
Aurora, CO 80045
Contact Person: Allan Roem, M.T.
720-848-7060
allan.roem@uch.edu

Sample Processing Lab:

University of Colorado Hospital
Clinical Translational Research Center (CTRC) Core Laboratory
Leprino Office Building (LOB) Room 326
12401 E. 17th Avenue, Mailstop B-141
Aurora, CO 80045
Contact Person: Kayla Carstens, M.T.
720-848-6877
kayla.carstens@ucdenver.edu

Specialty Labs:

University of Colorado Denver – Anschutz Medical Campus
Division of Allergy and Clinical Immunology
Clinimmune Labs
Center for AIDS Research Immunology Core
Research Complex II, Room 10007
12700 E 19th Avenue
Aurora, CO 80045
Contact Person: Brent Palmer, Ph.D.
303-724-7203
brent.palmer@ucdenver.edu

Colorado Antiviral Pharmacology Laboratory
University of Colorado Denver
School of Pharmacy
Pharmacy and Pharmaceutical Sciences Building, C238-V20
12850 E. Montview Blvd. Room V20-4410
Aurora, CO 80045
Contact Person: LR Bushman, B.Chem.
303-724-6134
lane.bushman@ucdenver.edu

Aquilante Pharmacogenomics Laboratory
University of Colorado Denver
School of Pharmacy

Pharmacy and Pharmaceutical Sciences Building, C238-V20
12850 E. Montview Blvd. Room V20-4420A
Aurora, CO 80045
Contact Person: Christina Aquilante, Pharm.D.
303-724-6126
christina.aquilante@ucdenver.edu

Medical Departments:

University of Colorado Hospital
Clinical Translational Research Center (CTRC)
Leprino Office Building (LOB) Room 301
12401 E. 17th Avenue, Mailstop B-141
Aurora, CO 80045
Contact Person: Timothy Woodworth, M.S.W., L.CSW, M.B.A.
720-848-6430
timothy.woodworth@uch.edu

University of Colorado Hospital
Endoscopy Lab
Anschutz Outpatient Pavilion
1635 Aurora Court
Aurora, CO 80045
Contact Person: Jenny Maxwell, M..B.A.
720-848-2560
jenny.maxwell@uch.edu

Pharmacy:

University of Colorado Hospital
Investigational Pharmacy
Department of Pharmacy, Mail Stop F757
Inpatient Pharmacy 1st Floor, Room 1.2301
Office 1.2301.3
12605 East 16th Avenue
PO Box 6510
Contact Person: Ken Easterday, R.Ph.
720-848-4232
FAX: 720-848-1418
ken.easterday@uch.edu

2. INTRODUCTION

2.1 Background Information

The main goal of this research is to extensively characterize the intracellular pharmacology of TDF and FTC, for the first time, in HIV-infected versus HIV-negative persons. This new information will fill a current knowledge gap and will help guide dosing recommendations for pre- and post-exposure prophylaxis in HIV negative persons.

There are many clinical and pharmacological factors that, when taken together, provide a high level of significance and sound rationale for this research. These background facts are first bulleted below and then are expanded upon under these headings.

- Pharmacologic effects for TDF and FTC depend upon their cellular pharmacology.
- Cellular pharmacology cannot be directly extrapolated from in vitro to in vivo setting.
- Important reasons to directly study the cellular pharmacology of NRTIs in vivo.
- TDF and FTC in a new in vivo setting: HIV prophylaxis.
- A summary of the high significance and rationale for this specific research.

2.1.1 Pharmacologic effects for TDF and FTC depend upon their cellular pharmacology

TDF and FTC are NRTIs, which must undergo sequential phosphorylation by host cell enzymes to the NRTI-TP for pharmacologic activity.(1, 2) Once phosphorylated, NRTIs are ion-trapped in cells. Thus, NRTIs have two distinct sets of pharmacokinetics, one for the parent drug in plasma and the other for the pharmacologically active NRTI-phosphates in cells. TDF is a prodrug that is converted to tenofovir (TFV) in plasma, which contains the initial phosphate group and thus requires only two phosphorylation steps intracellularly to form the active diphosphate (TFV-DP), which is actually a triphosphate. Intracellular NRTI-TPs inhibit HIV reverse transcriptase to elicit antiviral effect.(3) Interestingly, intracellular TFV-monophosphate (TFV-MP) may also inhibit purine nucleoside phosphorylase (PNP), an enzyme that degrades purine nucleosides. In an in vitro study, TFV-MP was found to inhibit the PNP-mediated breakdown of ddI with a ki = 38nM. This was determined to be the basis of the significant ddI-tenofovir plasma interaction in patients.(4) It is not known, however, whether this PNP inhibition by TFV includes endogenous purine nucleosides. PNP inhibition in vivo reduces T-cell activation and, with extreme inhibition, causes T-cell lymphopenia. It should be noted that some PNP inhibition may reduce T-cell activation and may thereby provide another mechanism of antiviral activity for TFV. In fact, PNP inhibition was once a targeted mechanism of action for antiretroviral drugs.(5) The only data for TFV-induced PNP inhibition of natural purines comes from one short-term in vitro study (24 hours in duration, thus pre-steady-state), which did not find that TFV altered endogenous purine concentrations.(6) Unfortunately, no in vivo studies have tested the effects of tenofovir on PNP activity either pre-steady state or steady-state. The present study will address this knowledge gap.

2.1.2 Cellular pharmacology cannot be directly extrapolated from in vitro to in vivo setting

Much of our information on the cellular pharmacology of NRTIs is from *in vitro* studies. Early *in vitro* studies identified that intracellular zidovudine-TP and stavudine-TP were dramatically higher and more potent against HIV in activated (phytohemagglutinin (PHA)-treated) vs. resting cells.(7, 8) On the other hand, TFV-DP has been shown to *decrease* by 6-fold in PHA-stimulated T-blasts when incubated for 24 hours versus 6 hours.(9) Another study showed that the TFV-DP

half life is 3-times longer in resting than activated cells *in vitro*.(10) These studies suggest that TFV-DP is higher in resting cells and that, more generally, cellular activation or quiescence changes the intracellular profile of NRTIs *in vitro*. Unfortunately, we do not know how these *in vitro* data relate to the *in vivo* setting. What is known is that the cellular pharmacology of NRTIs is of such high importance that NRTI dosing is based on the intracellular profile and not the plasma profile. Zidovudine, abacavir, and didanosine serve as good examples. Their plasma half-lives are about one hour and the parent drug is completely eliminated from plasma in about 5-10 hours. However, the half-lives of the pharmacologically-active NRTI-TPs are 7 to 24 hours, which allows once or twice daily dosing even though the concentrations of the parent drug in plasma are undetectable at the end of the dose intervals.(11) It must be noted, however, that NRTI-TP half-lives are consistently several-fold different in patient's PBMCs compared with *in vitro* cell experiments.(2) Therefore, in vitro data cannot be used as the sole basis for understanding NRTI cellular pharmacology in patients. The present study will focus on the cellular pharmacology of TFV and FTC in patients.

2.1.3 Important reasons to directly study the cellular pharmacology NRTIs in vivo

It is essential to directly study NRTI cellular pharmacology in vivo. For example, a theme of our research is that cellular activation associated with the presence (and/or severity) of HIV disease may influence the cellular pharmacology of TFV and FTC in humans. HIV causes generally elevated immune activation, with much higher markers of inflammation/cellular activation such as, increased IFN-α, TNF-α, soluble TNF receptor II (sTNFrII) levels, and CD38+ / HLA-DR+ expression compared with the absence of HIV infection.(12-15) Thus, the cellular pharmacology of TFV and FTC in the "activated state" of HIV infection may differ from that in the relative "resting state" of uninfected persons. An example with zidovudine helps illustrate the point. A previous study found the <u>lowest</u> total zidovudine-phosphates (mono-, di-, plus tri-) in healthy volunteers with strikingly <u>higher</u> concentrations in patients with advanced HIV.(16-18) These findings are consistent with the cellular pharmacology of zidovudine, where phosphorylation is elevated in the "activated state" and low in the "resting state". (7, 8) We hypothesize that TFV-DP, as a "resting-cell" dependent drug, may behave in an opposite fashion compared with zidovudine; TFV-DP concentrations may be higher in HIV-negative persons relative to HIV infected patients. Importantly, a direct comparison of the cellular pharmacology of TFV/FTC in HIV-negative vs HIV-infected persons and the effects of an "activated-" versus "resting-state" has not been evaluated. A main aim of the present study is to directly compare the cellular pharmacology of TFV and FTC in HIV-negative versus HIV-positive persons.

2.1.4 TDF and FTC in a new in vivo setting: HIV prophylaxis

Presently, several large phase-III studies including thousands of HIV-negative volunteers are underway evaluating TDF/FTC for pre-exposure HIV prophylaxis.(19) TDF/FTC are also under study (or used clinically) for episodic pre-exposure prophylaxis in HIV-discordant couples, as post-exposure prophylaxis for high-risk HIV exposures, and as single dose prophylaxis to prevent mother-to-child transmission.(20-22) Finally, TDF (brand name Viread) is used on the streets as a "triple V" concoction (Viread, Viagra, and Valium) for "episodic protection" during weekend sex parties.(23) The rationale for using these agents in this way is based upon several theoretical principles:(24) 1. Relative safety and favorable tolerability on a gross scale (few grade III-IV adverse events in HIV-infected patients).(25); 2. Promising prophylactic activity with acceptable safety in multiple animal models.(24, 26-33) 3. TFV-DP and FTC-TP purportedly accumulate in resting cells, which are early targets in HIV infection.(9); 4. The half-lives of the two intracellular

moieties are presumably very long (40 to 150 hours) which would provide pharmacologic coverage in times of poor adherence.(34, 35)

Importantly, these theoretically favorable cellular pharmacology properties have not been directly measured in HIV-negative persons. Long intracellular half-lives are viewed favorably because, at steady-state, they provide "pharmacokinetic forgiveness" for missed doses. However, long-half-lives also determine the "time to reach steady state" (i.e. the rate of accumulation to steady-state). In fact, the longer the half-life, the slower the accumulation to steady-state. The half-life for TFV-DP in *HIV infected patients* is approximately 6 days (it is not known for HIV-negative persons).(35) If the accumulation of intracellular TFV-DP follows traditional pharmacokinetic rules, steady state will be reached in ~ 5 half-lives (30 days) and the accumulation in the first six days of daily TDF will only be *half* of the eventual steady-state concentration. Such a slow accumulation would be crucial information for understanding the onset of pharmacologic action especially for episodic and single dose prophylaxis in the clinical setting. The present study will define the accumulation to steady state in HIV-negative adults.

2.1.5 A summary of the high significance and rationale for this specific research

The following points summarize the high human health importance for this research. The first statement indicates what will be examined in this study and the following statements give why it will be clinically significant.

1. The TFV-DP and FTC-TP accumulation to steady-state will be characterized. This will determine the onset of action, which may be several days if the accumulation rate is very slow, as predicted. It is of vital human health importance to understand the rate that TFV-DP and FTC-TP concentrations rise to steady-state, as TFV is already being used as episodic pre-and post-exposure prophylaxis in vivo.(20-22)

2. The TFV-DP and FTC-TP wash-out elimination half-life will be determined in HIV-negative volunteers. This will govern the duration of action and the potential window of drug resistance after the drugs are stopped and for missed doses. The importance of this concept is especially high for drugs with long half-lives such as, TFV-DP and FTC-TP, and is analogous to the well-known resistance issues with nevirapine (NVP) in mothers *and infants who become HIV-infected* after single NVP doses for HIV prophylaxis. (36)

3. The concentrations of TFV-DP and FTC-TP will be measured in CD4 purified cells (as well as erythrocytes –to include dried blood spot analyses, and CD8s, B cells, and monocytes), rectal mucosal mononuclear cells, and genital tract mononuclear cells, and PBMCs to determine if PBMC concentrations reflect the concentrations at the site of action. The concentrations of TFV-DP and FTC-TP have not been measured in CD4 cells or tissue mucosal cells in persons taking these drugs, therefore these new data will provide a pharmacologic basis and clinically relevant correlate for the current practice of measuring TFV-DP and FTC-TP in PBMCs.

4. The cellular pharmacology of TFV-DP and FTC-TP will be compared in HIV-negative and HIV-positive persons. We hypothesize that most cells in HIV-negative persons are in a "resting-state" and that TFV-DP concentrations will be higher compared with cells in HIV-infected persons that are in an "activated-state". Thus, the cellular pharmacology of TFV-DP and FTC-TP may be influenced by HIV-serostatus, which could suggest different dose and dosing rate needs according to indication (prophylaxis versus treatment).

5. Purine concentrations in urine and plasma will be measured as sensitive markers of PNP inhibition before and during TDF/FTC therapy. There is no information on purine changes corresponding with TFV-related PNP inhibition in persons. Possible PNP inhibition may be a beneficial pharmacologic effect by reducing T-cell activation or innocuous short-term, but serious to the immune system with long term therapy.(5) The important point is that PNP inhibition is a fundamental pharmacologic effect that must be characterized to fully understand the profile of the drug. Finally, if the cellular pharmacology of TFV-DP and FTC-TP differ according to HIV-infection status, as described above, then purine changes may be more pronounced in HIV-negative persons.

6. A prophylactic effect threshold for TFV-DP and FTC-TP concentrations will be identified. We will use a case-control study of prophylaxis-failures and matched controls (prophylaxis-successes) in a large phase III study of HIV prophylaxis in men at risk for sexual acquisition of HIV. We have established a collaboration with the principal investigator, Dr. Robert Grant, to undertake these important analyses. Identifying the effective TFV-DP and FTC-TP concentrations, when coupled with all the new cellular pharmacology information learned above, would provide a comprehensive pharmacokinetic-pharmacodynamic model that would inform the optimal dose and frequency of TDF/FTC for pre-, post-, and episodic-exposure prophylaxis with these drugs.

7. We will evaluate sex and race differences in TFV-DP and FTC-TP. Potential sex or race differences have not been adequately evaluated *in vivo* which prevents the opportunity to individualize therapy in specific patient populations. We will also extend our previous findings with MRP4 and MRP2 genetics with TFV-DP (and FTC-TP) and will open new pharmacogenetic avenues to improve our understanding of TFV and FTC cellular disposition and response.(37)

2.2 Hypothesis and Specific Aims

Hypothesis: The optimal use of NRTIs for HIV prophylaxis and treatment is achieved through an understanding of NRTI cellular pharmacology in patients.

Aim 1: To compare the intracellular profiles of TFV-diphosphate (TFV-DP) and FTC-triphosphate (FTC-TP) in HIV-negative versus HIV-positive adults.

Aim 2: To evaluate whether the pharmacologic spectrum of TFV includes inhibition of PNP in vivo.

Aim 3: To identify a prophylactic threshold for intracellular TFV-DP and FTC-TP in PBMCs in vivo.

Aim 4: To develop a comprehensive pharmacokinetic model for TFV -DP and FTC-TP according to cell-type and tissue compartment with which to predict the optimal dose and onset and duration of prophylactic action.

2.3 Potential Risks and Benefits

The risks associated with this study include the following: study medications in HIV-uninfected volunteers; rectal mucosal pinch biopsies; genital samplings (cervicovaginal and semen samples);

blood draws; and confidentiality issues. Each of these will be detailed below except for confidentiality issues, which will be addressed in section 13. The study provides a physical exam and medical history and laboratory testing as benefits to being in the study.

2.3.1 Study medication risks, HIV-positive persons

HIV-infected persons will receive continuous TDF/FTC/EFV by prescription from their clinician. These medications are considered first-line standard of care for antiretroviral naïve subjects.(38) Subjects will be eligible for our study only *after* the primary clinician has weighed the risks and benefits and has decided to use these specific medications. These subjects would receive the medications whether or not they were in the study, so the study does not impose medication-related risks. Nevertheless, the study will screen subjects for pregnancy, will counsel subjects to protect against pregnancy, and will provide safety laboratories to the referring clinician.

2.3.2 Study medication risks, HIV-negative persons

HIV-uninfected subjects will receive daily TDF/FTC through the day 30 visit and will be followed for an additional 30 days off medications. Since the HIV-uninfected have no indication for these drugs the study imposes medication-related risks as follows.

The risks of TDF in HIV-infected persons have been well-defined in multiple large studies encompassing hundreds of thousands of person-years of safety experience.(25) The safety of FTC has also been assessed extensively.(39) One unique side effect of FTC is hyperpigmentation of the soles and palms (~3% incidence overall), which is more common in non-Caucasian patients. It is typically asymptomatic and reversible. The most common side effects for both TDF/FTC are gastrointestinal complaints and headache (> or = 10%), which are generally mild and reversible.(40) Rare cases of renal insufficiency and renal failure have been reported for TDF in certain patients (<1%). The following may be risk factors for renal complications: advanced HIV; low body-weight, pre-existing renal dysfunction; and concomitant nephrotoxic drugs (e.g. aminoglycosides, amphotericin B, cidofovir) or certain antiretroviral drugs (e.g. ddI, lopinavir/ritonavir).(25, 40) Hepatitis flares have been reported in patients co-infected with hepatitis B virus (HBV) when TDF/FTC therapy has been withdrawn, as both agents are active against HBV (<1%). Bone mineral loss leading to pathologic bone fractures with TDF has been reported rarely in patients and the same side effects were evident in animal studies (<1%). Metabolic acidosis with hepatomegaly and steatosis has been reported rarely with all NRTIs (<1%). Changes in body fat (subcutaneous lipoatrophy and abdominal hypertrophy) and metabolic derangements (elevations in lipids and hyperglycemia) have been reported for many antiretroviral drugs especially after prolonged use (>10% depending on agent). TDF/FTC are less likely to cause body fat changes and metabolic derangements compared with other antiretroviral agents. Finally, as patients take these drugs for longer courses and as patients age, there may be new risks or long-term complications that arise. A summary of the side effect profile of TDF/FTC is provided below and in the product information:

The following side effects have been reported most commonly with TDF-FTC (~10%) [note these patients were also taking efavirenz, which is associated with rash, dizziness, depression, insomnia, fatigue].

- diarrhea,
- nausea,
- fatigue,
- sinusitis, or upper respiratory infection

- headache,
- dizziness,
- depression,
- insomnia,
- abnormal dreams,
- rash,
- hyperpigmentation of soles/palms,and
- abnormal lab values (amlyase, lipase, neutropenia, LFTs, creatine kinase, phosphorous).

The following are uncommon but potentially serious side effects:

- Lactic acidosis and hepatomegaly with steatosis,
- Renal insufficiency/failure,
- Severe hypophosphatemia,
- Osteomalacia – pathologic bone fractures ,
- Pancreatitis,
- Hepatitis (especially hepatitis B flares after stopping therapy),
- Immune reconstitution syndrome (HIV-infected patients with opportunistic infections such as TB),and
- Allergic reaction.

Less is known about the risks of 30 days of therapy in HIV-uninfected subjects although several thousand HIV-uninfected subjects have received continuous daily TDF/FTC for months in ongoing phase III studies or clinically for HIV prophylaxis.(19) One large safety assessment of TDF therapy in HIV-uninfected adults was conducted in Ghana, Nigeria, and Cameroon.(41) Subjects contributed 428 person-years of safety information for the analysis and no differences were found between placebo and TDF in laboratory (hepatic and renal) and clinical safety endpoints. Smaller studies of post-exposure prophylaxis with 30-day courses of TDF/FTC (or TDF/lamivudine) versus zidovudine-lamivudine indicate that twice as many patients taking TDF-containing regimens tolerate and finish the 30 days of treatment (approximately 80% versus 40%).(20)

A recent study called iPrEx, tested whether Truvada tablets taken daily were safe and if they reduced the chance of getting HIV infection in men who have sex with men. The iPrEx study enrolled 2,499 men who have sex with men at 11 sites in 6 countries: Peru, Ecuador, Brazil, the United States, Thailand and South Africa (Cape Town). There were no serious safety concerns in the group of men who took the Truvada tablets and these men had a lower risk of getting HIV (almost 44% lower) in comparison to men who took a placebo tablet.

Although the study showed that Truvada worked, it did not provide 100% protection against getting HIV. Additionally, the study was only done in men who have sex with men so it is not known if women or men who only have sex with women will be similarly protected.

2.3.2.1 Approaches to minimize drug risks

This study is taking several precautions to minimize the drug risks in the HIV-uninfected volunteers. First, we are testing volunteers for pregnancy, HIV-infection, and HBV-infection to protect subjects from drug exposure during pregnancy, suboptimal antiretroviral therapy (two-

drug versus three-drug standard of care) and HBV-associated hepatitis flares when the drugs are withdrawn after the day 30 visit. We are not accepting subjects who have serious underlying illnesses such as cancer and heart disease. We are screening subjects for hepatic and renal dysfunction, and comprehensive metabolic and hematologic panels to rule out pre-existing abnormalities. Subjects will be followed closely with clinical laboratories and interviews to assess tolerability and safety over the 30 days of drug therapy and for an additional 30 days off study medications. Subjects will also be called by phone between days 7 and 20 to assess how they are doing.

2.3.2.2 Summary assessment of drug risks

In summary, the most likely risks associated with 30 days of TDF-FTC in HIV-negative persons appear to be gastrointestinal complaints, headache, and possibly hyperpigmentation of the soles and/or palms in non-Caucasians. These side effects are most likely mild and reversible. To our knowledge, no serious toxicity issues have emerged in phase III studies in thousands of HIV negative volunteers. There is a remote risk of serious or fatal side effects including renal failure, lactic acidosis with hepatomegaly and steatosis, bone pathology, hypophosphatemia, hepatitis, pancreatitis, and there are possible side effects not listed here, or possible unforeseen long-term complications. We believe the risk of these serious side effects is remote given the rarity of the events during long-term treatment and the short 30 day course of therapy here. We have designed the inclusion and exclusion criteria to exclude subjects who might be at higher risk of some of these toxicities. We have also designed a rigorous monitoring strategy for early detection of side effects and for quick study discontinuation, if needed, to protect subjects.

2.3.3 Rectal mucosal biopsy risks

Each subject, males and females, will have one rectal mucosal biopsy while in the study. The biopsy will be assigned to one of the study days (either first dose, day 3, day 7, day 20, or day 30) and will be approximately 2-hours post dose of study medication. Each biopsy consists of approximately 20 small pinch biopsies at approximately 4-12 inches from the anal verge as previously described (42), for isolation of rectal mucosal mononuclear cells. The cells will be used for studies of intracellular drug concentrations. Briefly, biopsies are obtained with endoscopic biopsy forceps via flexible sigmoidoscopy and immediately placed into sterile solution according to SOP #021. The procedure does not require sedation or anesthesia. The main risks associated with the biopsy are discomfort from the injection of air into the rectum and cramping. Remote risks include perforation or bleeding - rarely resulting in death. To mitigate risks from the procedure, the biopsies will be performed by an experienced gastroenterologist, Jesus Rivera, M.D., Associate Professor Division of Gastroenterology. It is estimated that the biopsy heals in approximately 72 hours. Participants will be reminded of the importance of using condoms to prevent HIV, and will be advised not to have anal sex for at least 5 days after the biopsy. The procedure will be conducted in the University of Colorado Hospital endoscopy clinic according to their standard endoscopy procedures including pre-biopsy enema. Subjects taking daily anticoagulant therapy will not be eligible to enroll in the study.

2.3.4 Cervical brush sampling

Each woman subject will undergo one cervical brush collection and cervical fluid sampling to be performed by our study physician, Dr. Amie Meditz or the study coordinator, Julie Predhomme, N.P. The sampling will be assigned to one of the study days (either first dose, day 3, day 7, day 20, or day 30) and will be approximately 2-hours post dose of study medication. The sampling

will not take place on the same day as the rectal biopsy. The procedure involves a pelvic exam and a manual brush sampling to remove cells from the cervix as described in SOP #019. The cells will be used for intracellular pharmacology studies. Additionally, cervicovaginal fluid will be collected for free drug analysis using a volumetric aspiration device and a paper absorbing strip. A wet prep exam will test for trichomonas, yeast, and bacterial vaginosis. A cervical sample will be taken to test for gonorrhea and Chlamydia. Risks include minor discomfort, and minimal vaginal bleeding/spotting. Participants will be reminded of the importance of using condoms to prevent HIV, and will be advised not to have vaginal sex for at least 5 days after the cervical collection.

2.3.5 Semen sample

Each male subject will provide one semen sample for isolation of semen leukocytes as described in SOP #020. The sampling will be assigned to one of the study days (either first dose, day 3, day 7, day 20, or day 30) and will be approximately 24-hours post dose of study medication. The sampling will not take place on the same day as the rectal biopsy. Subjects will be asked to bring the sample to the study visit from home using a study-supplied sterile container, zip-locked bag, and hard-sided transport vessel. The subject will record the time the sample was collected on a study form. Cells will be harvested as described in SOP #020. The cells will be used for intracellular pharmacology analyses. Subjects will undergo a urine test for gonorrhea, Chlamydia, and trichomonas on the day of the semen sample.(43) Risks from the procedure are minimal.

2.3.6 Blood collections

Approximately 450 to 480 mL of blood will be collected from each subject over the 60 day study (about 450 mL for HIV-positive volunteers and 480 mL for HIV-negative volunteers). These volumes are well below the maximal volume set for blood donations and clinical research of 550 mL in 56 days. Hematology will be followed closely. Subjects will not be allowed to have donated blood within 56 days of starting the study. Persons under 110 pounds will not be eligible. The blood will be collected from an arm vein by trained professionals in the CTRC according to CTRC procedures. Risks include pain, bruising, syncope, and infection.

3. OBJECTIVES

3.1 Primary Objectives

1. A comprehensive characterization and comparison of the cellular pharmacokinetics (the accumulation up to and including steady state) of TFV-DP and FTC-TP in HIV-negative versus HIV-positive adults:

2. A comparison of urine and plasma purines, and intracellular endogenous purine nucleotides before therapy versus after 30 days of TDF/FTC therapy [powered for plasma deoxyguanosine].

3. A prophylactic threshold for TFV-DP and FTC-TP in PBMCs will be assessed in stored PBMCs from the "Chemoprophylaxis for HIV infection in men (iPrEx)" protocol. iPrEx is an ongoing Phase III randomized, double-blind, placebo-controlled study of the safety and efficacy of chemoprophylactic TDF/FTC administered orally once daily to men at high risk for acquiring HIV-1. iPrEx plans to enroll 3000 HIV-1 seronegative high-risk MSM who will be randomized to receive either TDF/FTC daily (N=1500) or TDF/FTC placebo daily (N=1500) in addition to standard counseling, condoms, and STI management. As part of the protocol of iPrEx, PBMCs

are currently stored every 24 weeks from baseline to the end of the follow up (at least 48 weeks). Additionally PBMCs are stored if a subject acquires HIV while on study. From iPrEx, we will identify all the cases of HIV sero-conversion in the TDF/FTC arm and will analyze stored PBMCs from these subjects from study date most proximal to the sero-conversion event (estimated to be 24 cases). Should additional PBMCs be available from other nearby visits, these will be evaluated for inclusion in the analyses, on a case-by-case basis. We will then match these cases with controls in the TDF/FTC arm who did not sero-convert on study in 1:4 ratio (cases:controls). A stored PBMC sample from the same study time point will be analyzed for the controls. Controls will be matched with cases for study site, age (within 5 years), and HIV risk factors (such as presence of concomitant sexually transmitted disease). The blinding in the iPrEx study will be protected; samples will be analyzed after the study is unblinded, or an SOP will be used by iPrEx investigators to protect the blind if analyses occur before the blind. Such an SOP has been developed and approved by DAIDS.

4. A pharmacokinetic model will be developed that describes the intracellular pharmacokinetics of TFV-DP and FTC-TP in PBMCs so that various dosing strategies can be tested on the model to identify the optimal dosing that most rapidly achieves and sustains the desired prophylactic threshold for HIV prevention.

3.2 Secondary Objectives

1. A definition of the terminal elimination phase of TFV-DP and FTC-TP in HIV-negative adults.

2. A characterization of TFV-DP and FTC-TP according to the following cell types: PBMCs, CD4-purified PBMCs (as well as erythrocytes- to include dried blood spot analyses, CD8 cells, B-cells, and monocytes), genital mononuclear cells, and rectal mucosal mononuclear cells.

3. A comparison of TFV-DP and FTC-TP between men and women.

4. A comparison of TFV-DP and FTC-TP between African-Americans and non-African-Americans.

5. A characterization of intracellular TFV, TFV-MP, FTC-MP, and FTC-DP

6. An evaluation of polymorphisms in MRP2 (e.g. -24C>T and 1249G>A), MRP4 (e.g. 1612C>T, 3463G>A, 3724G>A, and 4131T>G), BCRP (e.g. 421C>A and 34G>A) and other potentially important enzymes for the study drugs for relationships with pharmacokinetics and pharmacodynamics.

7. An evaluation of markers of cell activation (HLA-DR and CD38 expression) and the relationship to TFV-DP and FTC-TP concentrations.

8. The day 30 AUC and overall AUC (AUC over day 1 to day 30) TFV-DP and FTC-TP will be compared in HIV-negative versus HIV-positive subjects.

9. HLA-DR / CD38 on T cells will be correlated with changed intracellular and extracellular purine levels in the HIV-negative volunteers to address potential immune-modulation associated with PNP inhibition.

10. The ratios of TFV-DP and FTC-TP to corresponding endogenous deoxyribose nucleotides will be characterized.

11. The effects of TFV-DP and FTC-TP (and plasma EFV) on plasma HIV-RNA and CD4 counts will be determined in the HIV-positive cohort.

12. The TFV-DP and FTC-TP will be compared according to iPrEx study sites.

13. Relationships between adherence measures collected in iPrEx with TFV-DP and FTC-TP will be evaluated.

4. STUDY DESIGN

Objectives will be addressed with a prospective, observational pharmacokinetic study in HIV-infected and HIV-uninfected adult volunteers. This part of the study will be conducted at the University of Colorado Denver, Anschutz Medical Campus, Clinical Translational Research Center, DAIDS assigned site # 31642. Two equal sized groups of volunteers, 20 HIV-infected versus 20 HIV-uninfected adults, will be sought. The study will request COMIRB approval for a total of 60 subjects to successfully identify 40 subjects who qualify for and complete the study. During the study, the cellular pharmacology of TFV -DP and FTC-TP will be intensively characterized and compared according to HIV disease status and purine concentrations will be measured in urine, plasma, and cells before therapy and at TDF/FTC steady-state to assess PNP inhibition. The two serostatus cohorts will be enrolled to target 50% women and 50% African American when possible because secondary analyses will assess gender and race effects on TFV/FTC cellular pharmacology.

HIV-uninfected subjects will receive daily TDF 300mg / FTC 200mg (as Truvada®) through the day 30 visit, but will be followed for an additional 30 days off drug to determine the terminal elimination half-lives for intracellular TFV-DP and FTC-TP. The HIV-infected group will be prescribed TDF 300mg / FTC 200mg / efavirenz (EFV) 600mg under the direction of their clinician and will remain on their medications throughout the 60 day study period according to the direction of their clinician. The clinicians will be asked to write separate prescriptions for EFV and TDF/FTC (as Truvada®) for the first 30 days, so the pharmacology studies for TDF/FTC (Truvada®) can be conducted during the day and the EFV can be taken at bedtime as is recommended for EFV. Subjects would be free to switch to the TDF/FTC/EFV co-formulation after day 30; this would require a new prescription from their clinician. The rationale for EFV as the third antiretroviral drug for the HIV-infected subjects is that TDF/FTC/EFV is a first-line standard of care and the most commonly prescribed drug regimen in this community. Additionally, EFV is not expected to interfere with transporters and enzymes that influence TFV-DP and FTC-TP disposition. HIV uninfected subjects will not receive EFV as the potential scientific benefits do not outweigh the risks associated with EFV (teratogenicity, rash, CNS side effects). This study will not influence medical care decisions for the HIV-infected patients; all treatment decisions will be under the direction of the primary clinician, but the study will provide clinical and laboratory safety assessments to the primary clinician regularly. The study schema including study visits is shown in the figure on the next page.

	HIV-negative		HIV-infected,	
	men	women	men	women
African-American	5	5	5	5
Non-African-American	5	5	5	5

The desired characteristics of study volunteers: Subjects will be split when possible by HIV-serostatus, sex, and race (African-American versus non-African-American) for secondary analyses.

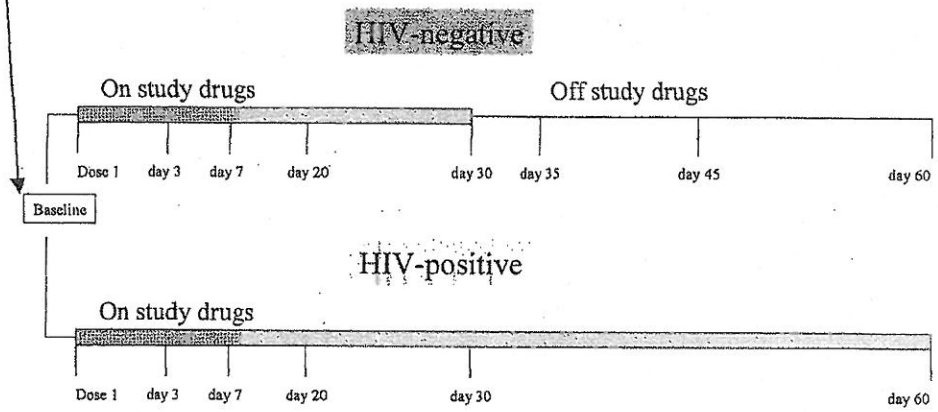

Overall study Schema: All subjects will undergo a baseline visit off drugs and will be started on TDF/FTC and followed with the indicated study visits for 60 days. A description of the study visits follows below.

The study will collect multiple types of samples during the study visits shown in the schema. The samples include a mouthwash sample for pharmacogenetics, routine blood and urine collections for pharmacology and purine metabolism assays, and special pharmacology collections for peripheral blood CD4 T cell purification (as well as purification of erythrocytes to include dried blood spots, CD8, B cells, and monocytes), and rectal, endocervical, and seminal mononuclear cells. On the day of genital sampling, men and women subjects will be screened for sexually transmitted infections (gonorrhea, chlamydia & trichomonas). Women will also be screened for vaginitis (yeast and bacterial vaginosis). The rationale for testing for the presence of sexually transmitted infections and/or inflammation is that it is important to document such infections because they may alter drug levels in genital mononuclear cells.

The first dose (day 1) and steady-state (day 30) pharmacology samplings will include 5 collections after an observed dose (approximately 1, 2, 4, 8, 24 hours post dose). Day 3, 7, and 20 visits will include 3 collections surrounding an observed dose (approximately 0, 2, 8 hours post dose). Participants will come to these visits fasting since 10pm the night before. The special pharmacology assessments (rectal biopsies, genital sampling, and the extra blood draw for CD4 cell purification) will take place only once each per subject, although the cell purification will also be done at baseline. Subjects will be assigned to have each procedure once on one of the visits between day 1 and day 30. The HIV-uninfected volunteers will undergo brief, one time point sampling visits on days 35, 45, and 60 while off study medication. The HIV-infected subjects will also undergo a brief one time point sampling visit on day 60. Day 60 is the final visit of the study for both serostatus cohorts. Some flexibility will be allowed for scheduling study visits. For example, day 7 may be scheduled from day 6 to day 8. The range of days allowed for each study visit is shown in the schedule of events in Appendix A and in section 7. Should it become impossible to schedule a visit within the given study day range, the visit will be scheduled as soon as possible. If the visit is within 7 days of the next scheduled visit, the visit will be skipped and the next scheduled visit will be scheduled.

Subjects will undergo safety assessments at multiple visits including comprehensive laboratory monitoring and structured adverse event questionnaires. Subjects will also be given a calendar to record unsolicited adverse events. All laboratory assessments including screening labs and safety assessments will be conducted in CLIA certified University of Colorado Hospital laboratories. Appendix A provides the schedule of events.

As mentioned in section 3 above, Aim 3 will be assessed with stored PBMCs from the "Chemoprophylaxis for HIV infection in men (iPrEx)" protocol. Stored PBMCs from subjects in the TDF/FTC arm who acquire HIV will be matched with controls in the TDF/FTC arm who did not acquire HIV in a 1:4 ratio (cases:controls). A stored PBMC sample from the same study time point will be analyzed for the controls. Controls will be matched with cases for study site, age (within 5 years), and HIV risk factors (such as presence of concomitant sexually transmitted disease). All stored samples from the iPrEx study will be de-identified for analysis in the Colorado Antiviral Pharmacology Laboratory.

5. STUDY POPULATION

5.1 Selection of the Study Population

This study will not enroll children, pregnant women, prisoners, or other vulnerable populations. Children will not be recruited because TDF/FTC is not recommended in children < 18 due to a lack of safety data. Recruitment will take place via COMIRB-approved email and advertisement postings on the University of Colorado Denver, Anschutz Medical Center Campus (and other campuses) and Denver Health. Additional COMIRB-approved strategies, such as newspaper or internet advertisements, will be used if needed. Subjects who contact us with interest in the study will be assessed for inclusion with a checklist and, based on the checklist answers, may be invited for a consenting and screening visit. Participants who screen fail may be re-screened at a later time, at the discretion of the investigators, and if approved by the safety officer. Only subjects who meet each of the following inclusion criteria and who do not meet any exclusion criterion will be allowed to enroll. Subjects must begin drug (day 1) within 21 days of the screening visit or screening labs will need to be repeated.

5.2 Inclusion/Exclusion Criteria

5.2.1 Inclusion criteria for HIV-uninfected cohort

1. Adults aged 18 to 55 years.

2. Ability to provide informed consent.

3. Ability to comply with the study procedures.

5.2.2 Exclusion criteria for HIV-uninfected cohort

1. Positive screening test for HIV infection.

2. Positive screening test for HBV infection.

3. Positive screening test for pregnancy.

4. Current breastfeeding.

5. A plan to become pregnant in the next 3 months.

6. If sexually active and fertile (no tubule ligation, hysterectomy) refusal to use two forms of birth control (e.g. condom and hormonal birth control) during the 60 day study.

7. Estimated GFR < 60 mL/min/1.73 m^2 by the MDRD method.

8. Albuminuria (> 30 mg urine albumin per g of urine creatinine)

9. Blood donation within 56 days of the screening visit.

10. Any grade I or higher abnormality in hemoglobin, platelets, serum phosphorous, and lipase on the screening visit; grade I abnormalities in other labs will be evaluated on a case by case basis (using DAIDS criteria).(44)

11. Any > grade I abnormality in screening laboratory tests (using DAIDS grading criteria).(44)

12. Medical history of chronic uncontrolled hypertension equal to or above 140/90.

13. Any investigational medication in the previous 30 days.

14. Daily anticoagulant therapy (daily aspirin or non-steroidal anti-inflammatory drugs (NSAIDs) will be allowed if discontinued for one week prior to the rectal biopsy).

15. Any nephrotoxic concomitant medication (examples include, aminoglycosides, cyclosporine, cidofovir, foscarnet, amphotericin B).

16. Active recreational drug or alcohol abuse.

17. Any concomitant medication (or herbal product) that, in the opinion of the investigators, would interfere with the study outcomes (acceptable medications include acetaminophen, occasional ibuprofen/NSAID, vitamins, birth control pills).

18. A history of pathologic bone fractures.

19. Any chronic or acute medical condition that, in the opinion of the investigator, would interfere with study conditions such as cancer, heart disease, diabetes.

20. Body weight under 110 pounds.

5.2.3 Inclusion criteria for HIV-infected cohort

1. HIV-infected adults aged 18 to 55 years (HIV documented in medical record or by the primary clinician).

2. Clinician / patient plan to initiate TDF/FTC/EFV therapy and agree to separate TDF/FTC and EFV prescriptions for the initial 30 days of the study.

3. Ability to provide informed consent.

4. Ability to comply with the study procedures.

5.2.4 Exclusion criteria for HIV-infected cohort

1. Antiretroviral therapy in the preceding 6 months.

2. Positive screening pregnancy test.

3. Current breastfeeding.

4. Plan to become pregnant in the next 3 months.

5. If sexually active and fertile (no tubule ligation, hysterectomy) refusal to use two forms of birth control (e.g. condom and hormonal birth control) during the 60 day study.

6. Estimated GFR < 60 mL/min/1.73 m^2 by the MDRD method.

7. Albuminuria (> 30 mg urine albumin per g of urine creatinine)

8. Greater than a grade II abnormality in hemoglobin or platelets. Greater than a grade II abnormality in other clinical chemistry or hematology tests that, in the opinion of the investigators (principal investigator, study coordinator, and study physician) and primary clinician, would preclude participation in the study. DAIDS grading criteria will be used. (44)

9. Any investigational medication in the previous 30 days.

10. Daily anticoagulant therapy (daily aspirin or NSAIDs will be allowed if discontinued for one week prior to the rectal biopsy).

11. Any nephrotoxic concomitant medication (examples include, aminoglycosides, cyclosporine, cidofovir, foscarnet, amphotericin B).

12. Any concomitant medication (or herbal product) that, in the opinion of the investigators, would interfere with the study outcomes (acceptable medications include acetaminophen, occasional ibuprofen/NSAID, vitamins, birth control pills).

13. Any chronic or acute medical condition that, in the opinion of the investigator, could lead to emergent health complications, or could interfere with the participant's ability to follow study procedures.

14. Body weight under 110 pounds.

6. STUDY PRODUCT INTERVENTION(S)

6.1 Regimen

HIV-uninfected subjects will receive daily TDF 300mg / FTC 200mg (as Truvada®) through the day 30 visit, but will be followed for an additional 30 days off drug to determine the terminal elimination half-lives for intracellular TFV-DP and FTC-TP. The HIV-infected group will be

prescribed TDF 300mg / FTC 200mg / efavirenz (EFV) 600mg under the direction of their clinician and will remain on their medications throughout the 60 day study period as directed by their clinician.

The clinicians will be asked to write separate prescriptions for EFV and TDF/FTC (as Truvada®) for the first 30 days, so the pharmacology studies for TDF/FTC (Truvada®) can be conducted during the day and the EFV can be taken at bedtime as is recommended for EFV (subjects would be free to switch to the TDF/FTC/EFV co-formulation after day 30).

6.2 Study Product Formulation and Preparation

The study product is Truvada®, manufactured by Gilead Sciences. Each Truvada® tablet contains a co-formulation of TDF 300mg / FTC 200mg. All Truvada® dosing in this study is the standard approved dose of one tablet once daily with or without food. HIV-uninfected subjects will be supplied with Truvada® as part of the study through a prescription from the study physician. HIV-infected subjects will be prescribed Truvada® by their own clinician through their usual care and will obtain their medication from their own pharmacy. Study drug will not be provided to the HIV-infected subjects. The clinicians will be asked to write separate prescriptions for EFV and TDF/FTC (as Truvada®) for the first 30 days as mentioned above. Subjects would be free to switch to the TDF/FTC/EFV co-formulation after day 30 through their own pharmacy and prescription from their clinician. The co-formulation will not be provided by the study.

6.3 Study Product Supply and Accountability

Study drug will be dispensed for the HIV-negative volunteers through the investigational pharmacy according to the research pharmacy policies and procedures of the University of Colorado Hospital. Ken Easterday, R.Ph. will be the pharmacist in charge for the study. Gilead Sciences will provide the study drug for HIV-negative volunteers. Mr. Easterday will receive and inventory the study medications, will dispense and track prescriptions, and will provide directions for returning unused medications for destruction. He will track the accountability of the study medications according to established procedures developed by the investigational pharmacy. As mentioned above, HIV-positive patients will be responsible for their own antiretroviral medications from their own pharmacy. The study will not supply these medications and will not track them other than counting them for adherence, as described below.

6.4 Assessment of Participant Adherence with Study Product/Intervention

Subjects will be given COMIRB-approved medication calendars to complete during the course of the study. The calendar will be used to track study visit and procedure activities/appointments, to record each daily dose of study medications, and for any unsolicited adverse effects encountered during the course of the study. The subjects will be asked to bring this calendar and their study medications to each study visit, so the calendar can be photocopied for the subject's binder. Additionally, all subjects will be asked to bring their Truvada® medication to study visits in the original container including medication provided by the study – i.e. HIV-negative volunteers, and medication provided by an outside pharmacy – i.e. HIV-positive volunteers. The study medication will be counted by study personnel at each visit.

6.5 Concomitant Medications and Procedures

Concomitant medications will be evaluated on a case-by-case basis for interactions with the study medications. Certain medications are permitted and certain medications are prohibited. Medications not listed in these sections will be evaluated for potential interactions with the study medications or procedures and a decision to enroll the subject will be made accordingly. Additionally, should a new concomitant medication be started during the study, the same evaluation will take place.

6.6 Permitted Medications and Procedures

Certain medications are allowed: acetaminophen, occasional ibuprofen (not daily ibuprofen unless it can be discontinued 7 days before the rectal biopsy), vitamins, and birth control contraception.

6.7 Prohibited Medications and Procedures

Prohibited concomitant medications include the nephrotoxic agents: aminoglycosides, cyclosporine, amphotericin B, foscarnet, and cidofovir, and anticlotting medications: warfarin, heparin, and ticlodipine. Products with same or similar active ingredients as the study medications (Truvada®) are prohibited including ATRIPLA®, EMTRIVA®, VIREAD®; or drugs containing lamivudine, which is a close analog of FTC. Drugs that are contraindicated or not recommended for use with Atripla® include voriconazole, ergot derivatives (dihydroergotamine, ergonovine, ergotamine, methylergonovine), midazolam, triazolam, bepridil, cisapride, pimozide and St. John's Wort. Furthermore, whenever a concomitant medication or study agent is initiated or a dose changed, investigators will review the concomitant medication's and study agents' most recent package inserts, or updated information from DAIDS to obtain the most current information on drug interactions, contraindications, and precautions.(40, 45)

6.8 Precautionary Medications and Procedures

Any medication not listed under the exclusion criteria will be evaluated on a case-by-case basis for interactions with the study medications. Should there be potential for interaction, based on the opinion of the investigators, the subject will not be invited to participate. Additionally, should a new concomitant medication be started during the study, the same evaluation will take place.

6.9 Required Medications and Procedures

HIV-infected persons must receive continuous TDF/FTC/EFV by prescription from their clinician through their own pharmacy. These medications are considered first-line standard of care for antiretroviral naïve subjects.(38) Subjects will be eligible for the study only *after* the primary clinician has weighed the risks and benefits and has decided to use these specific medications.

HIV-uninfected subjects will receive daily TDF/FTC through the day 30 visit and will be followed for an additional 30 days off medications. A 32-day supply will be provided to the subject by qualified study personnel at the day 1 visit.

6.10 Rescue Medications and Procedures

There are no study-mandated rescue medications or procedures. Should subjects need medical care for any reason the physician in charge will be allowed to give the medical care that they deem is necessary for the medical situation. The subject's further participation in the study would be evaluated according to section 9 below.

7. STUDY PROCEDURES/EVALUATIONS

7.1 Clinical Evaluations and Procedures

The study consists of 10 visits for HIV-negative volunteers and 8 visits for HIV-positive volunteers. HIV-negative volunteers undergo visits at day 35 and day 45 whereas HIV-positive volunteers do not. All study visits and a summary of the study evaluations are provided below.

Visit #1: Screening visit (must be ≤ 21 days of day 1 visit)

- Informed consent
- Urine sample for UA (with albumin/creatinine)
- Blood draw for CBC, CMP, lipase, phosphorous, pregnancy, HIV and HBV (HIV-negative group only), HIV-RNA (HIV-positive group only)
- Medical history and physical exam
- Medication history

Visit #2: Baseline visit (must be ≤ 7 days of day 1 visit)

- Following an overnight fast.
- Blood for cell sorting (purines), CD38/HLA-DR (cell activation), PBMC's (purines), stored PBMCs, plasma (purines), serum pregnancy test (if warranted)
- Medication history
- Mouthwash sample for pharmacogenetics
- Urine sample for purines.

Visit # 3: Day 1 visit (first dose):

- Following an overnight fast.
- Medication history
- Medication diary
- Dispensing of medication to HIV-negative cohort and pill count for both cohorts
- Adverse effect questionnaire
- Blood for PBMCs (pharmacology) and plasma at approximately 1, 2, 4, 8 and 24 hours post dose (pharmacology)
- 4 HIV-negative and 4 HIV-positive subjects = rectal biopsy sampling
- 2 HIV-negative women and 2 HIV-positive women = cervical sampling
- 2 HIV-negative men and 2 HIV-positive men = semen sampling
- 4 HIV-negative and 4 HIV-positive subjects = blood for cell sorting

- Pregnancy test, if clinically warranted

Visit # 4: Day 3 visit (days 2 to 4):

- Following an overnight fast.
- Medication history
- Medication diary
- Pill count
- Adverse effect questionnaire
- Blood for PBMCs and plasma at approximately pre-dose, 2, and 8 hours post-dose (pharmacology), CMP, phosphorous, lipase, CBC, CD38/HLA-DR (cell activation) Pregnancy test, if clinically warranted
- Urine for UA
- 4 HIV-negative and 4 HIV-positive subjects = rectal biopsy sampling
- 2 HIV-negative women and 2 HIV-positive women = cervical sampling
- 2 HIV-negative men and 2 HIV-positive men = semen sampling
- 4 HIV-negative and 4 HIV-positive subjects = blood for cell sorting

Visit # 5: Day 7 visit (days 6 to 8)

- Following an overnight fast.
- Medication history
- Medication diary
- Pill count
- Adverse effect questionnaire
- Blood for PBMCs and plasma at approximately pre-dose, 2, and 8 hours post dose (pharmacology), CMP, phosphorous, lipase, CBC, CD38/HLA-DR (cell activation), stored PBMCs. Pregnancy test, if clinically warranted
- Urine for UA and purines
- 4 HIV-negative and 4 HIV-positive subjects = rectal biopsy sampling
- 2 HIV-negative women and 2 HIV-positive women = cervical sampling
- 2 HIV-negative men and 2 HIV-positive men = semen sampling
- 4 HIV-negative and 4 HIV-positive subjects = blood for cell sorting

Visit # 6: Day 20 visit (days 18 to 22)

- Following an overnight fast.
- Medication history
- Medication diary
- Pill count
- Adverse effect questionnaire
- Blood for PBMCs and plasma at approximately pre-dose, 2, and 8 hours post dose (pharmacology), CMP, phosphorous, lipase, CBC, CD38/HLA-DR (cell activation) Pregnancy test, if clinically warranted
- Urine for UA
- 4 HIV-negative and 4 HIV-positive subjects = rectal biopsy sampling

- 2 HIV-negative women and 2 HIV-positive women = cervical sampling
- 2 HIV-negative men and 2 HIV-positive men = semen sampling
- 4 HIV-negative and 4 HIV-positive subjects = blood for cell sorting

Visit # 7: Day 30 visit (days 28 to 32)

- Following an overnight fast.
- Medication history
- Medication diary
- Pill count
- Adverse effect questionnaire
- Blood for PBMCs and plasma at approximately 1, 2, 4, 8, and 24 hours post dose (pharmacology); CMP, phosphorous, lipase, CBC, CD38/HLA-DR (cell activation), and stored PBMCs. Pregnancy test for HIV-positive participants or if clinically warranted for HIV-negative participants.
- Urine for UA and purines
- 4 HIV-negative and 4 HIV-positive subjects = rectal biopsy sampling
- 2 HIV-negative women and 2 HIV-positive women = cervical sampling
- 2 HIV-negative men and 2 HIV-positive men = semen sampling
- 4 HIV-negative and 4 HIV-positive subjects = blood for cell sorting

Visit # 8: Day 35 visit (days 33 to 37) for HIV-negative cohort ONLY

- Medication history
- Adverse effect questionnaire
- Blood for PBMCs and plasma (pharmacology) and stored PBMC's. Blood for CMP, phosphorous, lipase, CBC and/or pregnancy if clinically indicated.
- Urine for purines. Urine for UA if clinically indicated.

Visit # 9: Day 45 visit (days 43 to 47) for HIV-negative cohort ONLY

- Medication history
- Adverse effect questionnaire
- Blood for PBMCs and plasma (pharmacology)
- Blood for CMP, phosphorous, lipase, CBC and/or pregnancy if clinically indicated
- Urine for UA if clinically indicated

Visit # 10: Day 60 visit (days 57 to 67)

- Medication history
- Medication diary for HIV-positive cohort only
- Pill count for HIV-positive cohort only
- Adverse effect questionnaire
- Blood for PBMCs and plasma (pharmacology), CD38/HLA-DR, stored PBMCs, HIV-RNA (HIV-positive group only). Blood for CMP, phosphorous, lipase,

pregnancy, and/or CBC in HIV- positive participants, or if clinically indicated in HIV-negative participants.
- Urine for purines. Urine for UA in HIV-positive participants, or if clinically indicated in HIV-negative participants.

7.1.1 Medical History and Physical Exam

At the screening visit the study nurse practitioner will perform a history and physical for each participant. A complete medication history will be obtained including all drug allergies, immunization history, prescription, over the counter, and herbal treatments. The physical exam will include a comprehensive exam including all major organ systems. Vital signs will include height, weight, blood pressure, pulse, respiratory rate and temperature. A rated assessment (scale of 1-10) of current pain level will be done at this visit.

7.1.2 Mouthwash Sampling for DNA

Cells for DNA will be obtained with a Scope ® mouthwash collection on the baseline visit. Genomic DNA will be isolated from mouthwash expectorate using a commercially available kit (QIAmp DNA Mini Kit; Qiagen, Valencia, CA), according to standard manufacturer protocol. The DNA will be for pharmacogenetics studies. Details for the mouthwash collection are described in SOP #035.

7.1.3 Rectal mucosal biopsy

Each subject, both men and women, will undergo one rectal biopsy during the study by flexible sigmoidoscopy during the study, as described in section 2.3.3 and SOP #021. This sample will be obtained approximately 2 hours after dosing. The biopsy will be processed for mucosal mononuclear cells for intracellular drug concentrations. Subjects will be assigned the study day for the biopsy; either first dose, day 3, day 7, day 20, or day 30. An equal number of HIV-negative and HIV-positive subjects will be assigned the biopsy on each of these visits. Therefore, with five potential study days for the biopsy (first dose, day 3, day 7, day 20, or day 30) and 20 total HIV-negative and 20 total HIV-positive volunteers, the balanced assignment of subjects to each study day will result in 4 HIV-negative subjects and 4 HIV-positive subjects undergoing the biopsy on each visit.

7.1.4 Cervical brush sampling

Each woman subject will undergo one cervical brush collection and cervical fluid sampling to be performed by the study physician, Dr. Amie Meditz or the study coordinator, Julie Predhomme, N.P as described in section 2.3.4 and SOP #019. The cervical cells and cervicovaginal fluid will be used for pharmacology studies. A cervicovaginal sample will also be used for assessment of genital infections. Subjects will be assigned the study day for the sampling; either first dose, day 3, day 7, day 20, or day 30. An equal number of HIV-negative women and HIV-positive women will be assigned the sampling on each of these visits. Therefore, with five potential study days for the biopsy (first dose, day 3, day 7, day 20, or day 30) and 10 total HIV-negative women and potentially 10 total HIV-positive women volunteers, such balanced assignment of subjects to each study day will result in 2 HIV-negative women and potentially 2 HIV-positive women undergoing the biopsy on each visit.

7.1.5 Semen sample

Each male subject will provide one semen sample for isolation of semen leukocytes as described in 2.3.5 and SOP #020. The sampling will be assigned to one of the study days (either first dose, day 3, day 7, day 20, or day 30). The sample will be used for pharmacology assessments. An equal number of HIV-negative men and HIV-positive men will be assigned the sampling on each of these visits. Therefore, with five potential study days for the sampling (first dose, day 3, day 7, day 20, or day 30) and 10 total HIV-negative men and potentially10 total HIV-positive men volunteers, such balanced assignment of subjects to each study day will result in 2 HIV-negative men subjects and potentially 2 HIV-positive men subjects undergoing the sampling on each visit.

7.1.6 Blood collections

Approximately 450 to 480 mL of blood will be collected from each subject over the 60 day study for safety monitoring and pharmacology studies (about 450 mL for HIV-positive volunteers and about 480 mL for HIV-negative volunteers), as described in section 2.3.6. The blood will be collected from an arm vein by trained professionals according to CTRC policies and procedures.

7.1.7 Sexually Transmitted Infection and Vaginitis Testing

Cervical, vaginal and urine samples will be obtained to screen for sexually transmitted infections (gonorrhea, chlamydia & trichomonas) in both men and women. Women will also be screened for yeast and bacterial vaginosis. This testing will be done on women the same time we are collecting cervical cells and cervicovaginal fluid in a pelvic exam. The testing will be done on men from a urine sample obtained the same day that they bring their semen sample from home. A positive test would not exclude the subject from the study unless they required treatment with a systemic drug that is contraindicated. If treatment is required for a discovered genital infection, a member of the research team could provide the treatment and counseling, but the study would not pay for it. Alternatively, the subject could obtain treatment from their regular provider.

7.1.8 Urine Testing

Urine samples will be obtained at every study visit except Day 1 and Day 45 (unless clinically indicated) for testing purines, sexually transmitted diseases, and renal function as shown in Appendix A and in section 7 above. For the clinical samples, the participant will be asked to void a small amount of urine into a sterile specimen container per the standard CTRC policy. These samples will be transported to the hospital laboratory for analyses. The urine purine samples will be collected into a sterile container and transported to the Colorado Antiviral Pharmacology Lab for processing.

7.1.9 Medication Calendar, Pill Count, Adverse Events Questionnaire

Subjects will undergo safety assessments at multiple visits including comprehensive laboratory monitoring (described above and in Appendix A) and structured adverse event questionnaires. Subjects will also be given a calendar for study visit appointments, to record unsolicited adverse events, and record medication dosing times. The study medication for both HIV-negative and HIV-positive subjects will be counted by study personnel at each visit.

7.2 Laboratory Evaluations

7.2.1 Laboratory Evaluations and Specimen Collection

The following safety and monitoring laboratories will be collected at regular intervals during the study as described above and in Appendix A: Urine for urinalyses and sexually transmitted disease testing, complete metabolic panels with phosphorous and lipase, complete hematology panels, pregnancy tests, and HIV and HBV tests. The HIV and HBV tests will be used to screen HIV-negative volunteers for inclusion in the study. The tests are provided by the University of Colorado Hospital clinical laboratory and are standard immunoassay tests for the detection of antibodies for HIV and HBV infections. These clinical screening and monitoring tests will be processed and analyzed by the University of Colorado Hospital clinical laboratory, which is CLIA-certified, and the results will be posted to the medical record as positive or negative.

HIV-positive participants will not be screened for HIV because they will have already been diagnosed with HIV through their primary physician and will be ready to begin therapy with TDF/FTC/EFV. HBV will not be screened because the diagnosis and potential treatment of HBV is under the control of the primary clinician.

Study-related evaluations include a mouthwash sample for pharmacogenetics, routine blood and urine collections for pharmacology and purine metabolism assays, and special pharmacology collections for peripheral blood CD4 T cell purification (as well as erythrocytes- to include dried blood spot analyses, purification of CD8, B cells, and monocytes), and rectal, endocervical, and seminal mononuclear cells. Cervicovaginal and seminal fluid will also be collected.

The first dose (day 1) and steady-state (day 30) pharmacology samplings will include 5 blood collections after an observed dose (approximately 1, 2, 4, 8, 24 hours post dose). The days in between these visits will include 3 collections surrounding an observed dose (approximately 0, 2, 8 hours post dose). The special pharmacology assessments (rectal biopsies, genital sampling, and the extra blood draw for CD4 (as well as erythrocytes-to include dried blood spot analyses, and CD8/B cell/monocyte) purification will take place only once each per subject, although the extra blood draw will also occur at baseline. Subjects will be assigned to have each procedure once on one of the visits between day 1 and day 30. The rectal biopsy will need to be coordinated to be done during Dr. Rivera's endoscopy clinic. The cervical sampling visit will need to be coordinated so that it does not fall on a day of menses. The HIV-uninfected volunteers will undergo brief, one time point blood sampling for pharmacology with or without safety laboratories as described in Appendix A on days 35 (33-37), 45 (43-47), and 60 (57-67) while off study medication. The HIV-infected subjects will also undergo a brief one time point sampling visit on day 60 (57-67). Day 60 (57-67) is the final visit of the study for both serostatus cohorts.

7.2.2 Specimen Preparation, Handling and Shipping

Specimens will be collected in the CTRC Outpatient clinic or University Hospital endoscopy clinic by skilled nursing staff according to University Hospital policies and procedures. All specimen collections are described in the protocol, physician orders, and nursing flowsheets, and are detailed in SOP #026. Equipment and supplies for specimen collection are provided by the CTRC. Supplies for special samplings such as cell preparation tubes are supplied by CAVP laboratory. Initial sample processing such as harvesting plasma from blood may be provided by the CTRC core laboratory. The University of Colorado Denver – Anschutz Medical Campus houses all four laboratories contributing to this project. Samples will be transported to various

laboratories. Transport of clinical testing specimens from the CTRC to the University Hospital Clinical Laboratory and to the core laboratory will be accomplished according to CTRC and University Hospital policy and procedure. CTRC personnel will be responsible for tracking these specimens according to CTRC policies and procedures.

Samples destined for specialty labs such as the CAVP laboratory will be transported by study personnel according to standardized procedures described in SOP #027. Specimens will be transported in a hard-shelled container affixed with biohazard warning labels and contact information for the laboratory. Specimens must be properly labeled and placed in a plastic biohazard bag for transport. A copy of the CRF must accompany the specimen. Once delivered, the laboratories check in and track the samples according to the laboratory's policies and SOPs.

At this time, samples will not be shipped to outside entities, as all the samples will be processed and analyzed at the University of Colorado. Should outside shipping become necessary, the outbound shipment of biological, chemical, or radioactive materials is regulated by the International Air Transportation Association. Such training and certification of CAVP Laboratory employees is handled through the University of Colorado Denver Environmental Health and Safety Division. This training is required on a biannual basis. Specimens to be shipped by CAVP are shipped using FedEx. The protocol follows IATA Guidance 48-DGR when air transport is required. International shipment is not required to fulfill this protocol. Hazardous shipper's training is documented in the laboratory personnel record.

7.3 Biohazard Containment

Universal precautions, as recommended by the Center for Disease Control, the NIH, and the University of Colorado Denver Anschutz Medical Campus including the appropriate disposal of needles (etc) and human wastes will be exercised by Clinical Translational Research Center, University of Colorado Hospital, and study personnel to prevent the accidental transmission of HIV (or other viruses). All dangerous goods materials, including diagnostic specimens and infectious substances, will be transported according to instructions detailed in the International Air Transport Association Dangerous Goods Regulations.

7.4 Sequencing of Procedures/Evaluations: Timing and Definitions

See Appendix A: Schedule of Events Table

8. ASSESSMENT OF SAFETY

8.1 Adverse Event Definition

From the DAIDS Expedited Adverse Event Reporting policy No.:DWD-POL-SR-02.00:

Adverse Event (AE) – Any untoward medical occurrence in a patient or clinical investigation subject administered a pharmaceutical product and that does not necessarily have a causal relationship with this treatment. An AE can, therefore, be any unfavorable and unintended sign (including an abnormal laboratory finding), symptom, or disease temporally associated with the use of a medicinal (investigational) product, whether or not related to the

medicinal (investigational) product. In addition, if an untoward medical occurrence occurs as a result of study participation or study-related interventions, it is considered to be an adverse event.

Clinical adverse events will be collected with standardized questionnaires and laboratory adverse events will be collected and posted to the medical record during the course of the study. Subjects will have contact information for study personnel in the event of adverse events, and can contact personnel any time during the study. The following adverse events information will be recorded by study personnel: date of onset, assessment of severity, relationship to study medication, date of resolution/stabilization of event. The Division of AIDS Table for Grading the Severity of Adult and Pediatric Adverse Events (DAIDS AE Grading Table), Version 1.0, December, 2004 (Clarification dated August 2009) must be used and is available on the DAIDS RSC Web site: **http://rsc.tech-res.com/safetyandpharmacovigilance/**. (44)

8.2 Adverse Events Procedures and Requirements for Expedited Reporting

The adverse events (AEs) that must be reported in an expedited fashion to DAIDS Regulatory Support Center (RSC) Safety Office include all serious adverse events (SAEs) as defined by ICH guidelines regardless of relationship to the study agent(s):

- Results in death,
- Is life-threatening,
- Requires inpatient hospitalization or prolongation of existing hospitalization,
- Results in persistent or significant disability/incapacity, or
- Is a congenital anomaly/birth defect
- Important medical events that may not be immediately life-threatening or result in death or hospitalization but may jeopardize the patient or may require intervention to prevent one of the outcomes listed in the definition above must also be reported in an expedited timeframe to DAIDS. Such determination may be made through medical or scientific judgment. Examples include the following: intensive treatment in an emergency room or at home for allergic bronchospasm; blood dyscrasias or convulsions that do not result in hospitalization; development of drug dependency or drug abuse; etc.

* The following types of hospitalization do not require expedited reporting to DAIDS:

- *Any admission unrelated to an AE (e.g. for labor/delivery, cosmetic surgery, administrative or social admission for temporary placement for lack of a place to sleep)*
- *Protocol-specified admission (e.g. for procedure required by protocol, or)*
- *Admission for diagnosis or therapy of a condition that existed before receipt of study agent(s) (and has not increased in severity or frequency as judged by the clinical investigator, Note: a new AIDS-defining event in a subject already known to be HIV-infected would be considered an increase in severity of a pre-existing condition (HIV infection) and would therefore be reportable.)*

Important medical events that may not be immediately life-threatening or result in death or hospitalization but may jeopardize the patient or may require intervention to prevent one of the outcomes listed in the definition above must also be reported in an expedited timeframe to DAIDS. Such determination may be made through medical or scientific judgment.

A safety officer charter has been developed for this study, which is included with this protocol as Appendix B. The charter outlines strategies of safety monitoring by the study team to assure subject safety and study progress such as benchmark enrollment goals. The safety officer is Dr. Cara Wilson, M.D., Associate Professor of Medicine. There will be a safety officer meeting every 6 months to monitor safety and study progress. All AEs regardless of grade will be reviewed at these meetings.

8.3 Expedited Adverse Event Reporting

1. Adverse Event Reporting to DAIDS

Requirements, definitions and methods for expedited reporting of Adverse Events (AEs) are outlined in Version 2.0 of the DAIDS EAE Manual, which is available on the RSC website at http://rsc.tech-res.com/safetyandpharmacovigilance/.

The Manual of Operations (MOP) contains reference to Adverse Event reporting in section 5.4. This will now incorporate use of Version 2.0 of the Manual for Expedited Reporting of Adverse Events to DAIDS (DAIDS EAE Manual). SOP's # 014 and # 015 will also incorporate the use of Version 2.0.

The DAIDS Adverse Experience Reporting System (DAERS), an internet-based reporting system must be used for expedited AE reporting to DAIDS. In the event of system outages or technical difficulties, expedited AEs may be submitted via the DAIDS EAE Form. For questions about DAERS, please contact DAIDS-ES at DAIDS-ESSupport@niaid.nih.gov. Site queries may also be sent from within the DAERS application itself.

Where DAERS has not been implemented, sites will submit expedited AEs by documenting the information on the current DAIDS EAE Form. This form is available on the RSC website: http://rsc.tech-res.com/safetyandpharmacovigilance/. For questions about EAE reporting, please contact the RSC (DAIDSRSCSafetyOffice@tech-res.com).

2. Reporting Requirements for this Study

- The SAE Reporting Category, as defined in Version 2.0 of the DAIDS EAE Manual, will be used for this study.
- The study agents for which expedited reporting are required are:

  TDF/FTC (tenofovir disoproxil fumarate/ emtricitabine and EFV (efavirenz)

3. Grading Severity of Events

The most current Division of AIDS Table for Grading the Severity of Adult and Pediatric Adverse Events (DAIDS AE Grading Table) is used and is available on the RSC website at http://rsc.tech-res.com/safetyandpharmacovigilance/.

The Manual of Operations, (MOP) and SOP's reference the current DAIDS AE Grading Table.

4. Expedited AE Reporting Period

- The expedited AE reporting period for this study is the entire study duration for an individual subject (from study enrollment until study completion or discontinuation of the subject from study participation for any reason).

- After the protocol-defined AE reporting period, unless otherwise noted, only SUSARs as defined in Version 2.0 of the EAE Manual will be reported to DAIDS if the study staff become aware of the events on a passive basis (from publicly available information).

8.4 Local Regulatory Requirements

The local regulatory bodies include the Colorado Multiple Institutional Review Board (COMIRB), the Clinical Translational Research Center (CTRC), and the University of Colorado Hospital Research Review Committee (HRRC). COMIRB is the institutional review board for human subjects research. Unexpected problems including serious or unexpected adverse events must be reported electronically to COMIRB within 5 days. The CTRC RSA will be alerted to these unexpected problems as part of the electronic reporting procedure. The contact information for the local regulatory bodies are listed below.

COMIRB (IRB Identification Number: IRB00000848)
Mail Stop F490
Room N3214
13001 East 17th Place
P.O. Box 6508
Aurora, CO 80045
Director, Alison Lakin, Ph.D.
Phone: (303) 724-1055

CTRC
Mail Stop B141
3rd Floor, Leprino Building
12401 East 17th Avenue
Aurora, CO 80045
Director, Robert Eckel, M.D.
Phone: (720) 848-6661

HRRC
Mail stop F417
10-019, Leprino Building
12401 East 17th Avenue
Aurora, CO 80045
Administrator of Research, Mary P. Schumer, M.S., C.P.A.
Phone: (720) 848-7807

9. CLINICAL MANAGEMENT

9.1 Toxicity Management

HIV-uninfected subjects who have a grade I clinical or laboratory toxicity at screening (unless hemoglobin, platelets, lipase, or phosphorous for which grade I are exclusion criteria at screen) or any subsequent visit will be evaluated by the study team on a case by case basis. The toxicity will be evaluated for subject safety. The subject will not be enrolled in or will be removed from the study if the investigators believe the grade I toxicity jeopardizes the study's intent and/or the study is no longer in the subject's best interest. Grading will be according to the DAIDS table.(44) Higher grades of AEs will be handled as described later in this section.

For HIV-positive volunteers, all drug toxicity issues are under the management of the subject's provider. HIV-infected subjects who have a grade II or higher abnormality in hemoglobin or platelets at screening will not be eligible. If other laboratories are greater than a grade II at screening, the principal investigator, study coordinator, study physician and primary clinician will decide whether the subject can enroll on a case by case basis. The subject will not be enrolled in the study if the investigators and primary clinician believe the abnormality jeopardizes the study's intent and/or the study is not in the subject's best interest.

9.2 Other Disease Events

Certain disease states are prohibited and would result in ineligibility as described in section 5. Other relevant infections, clinical events and treatment complications not mentioned previously, will be managed on a case by case basis by the study team members including Julie Predhomme, N.P., the study physicians, and the participant's provider, if applicable.

9.3 Pregnancy

Pregnancy is an exclusionary criterion for this study. Women will be tested for pregnancy before taking part in this study, and during the study if pregnancy is suspected. HIV-positive participants will also be tested for pregnancy routinely at days 30 and 60. A woman cannot be in the study if found to be pregnant, or if planning to become pregnant in the next few months. For the HIV infected subjects taking efavirenz, we caution subjects that this drug may cause fetal harm when taken during the first 3 months of pregnancy. Serious birth defects, including those of the central nervous system, have been seen in the offspring of women taking efavirenz and in animal studies. If this drug is used during the first trimester of pregnancy, or if the patient becomes pregnant while taking this drug, the patient should be apprised of the potential harm to the fetus. There are no adequate or well-controlled studies in pregnant woman. Efavirenz should be used during pregnancy only if the potential benefit justifies the potential risk to the fetus, such as in pregnant woman without other therapeutic options. Hormonal contraception such as birth control pills, injections or implants are allowed in this study but barrier contraception should be used as well because efavirenz may make these hormonal contraceptives less effective.

We advise at least two birth control methods for both HIV-negative and HIV-positive women during the study and for at least 12 weeks after medication is stopped. Examples include:

- Double barrier method (i.e., use a male/female condom with either a diaphragm or cervical cap)
- Hormonal–based contraceptive in combination with a barrier method (i.e., male condom, diaphragm, cervical cap or female condom).
- IUD (intrauterine device) in combination with a barrier method (i.e., male condom, diaphragm, cervical cap or female condom).

- Practice heterosexual abstinence, or have a vasectomized partner who is confirmed sterile.

For male subjects, it is recommended to use a barrier method like condoms or have your partner use hormonal contraception if engaging in sexual activity that could lead to pregnancy while exposed to the agents, and for 12 weeks after to allow for the drug wash out. This will also help minimize the risk of acquiring and/or transmitting sexually transmitted diseased including HIV.

9.4 Breastfeeding (if applicable)

Breastfeeding is an exclusionary criterion for this study.

9.5 Criteria for Study Discontinuation

If any of the following occurs, the study will be stopped (no new enrollment) and reassessed for safety by the study investigators, safety officer, and DAIDS medical officer. 1. In the HIV negative cohort, if three or more persons experience a grade III adverse event or two or more persons experience a grade IV adverse event related to study medications. There will be no study discontinuation criteria for medication-related adverse events in HIV-positive volunteers, who will be receiving their medications as part of their routine clinical care. 2. Two or more persons from either the HIV-negative or HIV-positive cohort experience a grade IV complication from a study procedure (i.e. rectal biopsy, genital sampling, or blood draws).

9.6 Criteria for Permanent Treatment Discontinuation for an Individual Participant

Subjects may withdraw from the study at any time. COMIRB, DAIDS/NIAID, OHRP and the FDA may discontinue the study at any time. The study investigators or primary physician can remove a subject at any time if they deem that the study is no longer in the subject's best interest or the subject is not adhering to the protocol (i.e. has missed two consecutive study visits).

> HIV-uninfected subjects who develop a grade III or higher clinical or laboratory toxicity must discontinue the study drug and will be followed clinically until the abnormality falls below grade II. Grade II clinical or laboratory toxicities will be discussed among the study physician, safety officer, principal investigator, and nurse practitioner study coordinator within 24 hours. If the abnormal laboratory or clinical adverse event is possibly related with the study drug the study team may repeat the laboratory or assessment within 72 hours. Based on the results of the repeat assessment, the participant may be allowed to continue the study if they wish to, and the study physician, safety officer, and principal investigator all agree that it is acceptably safe. A study investigator will advise the participant of the decision made with the safety officer and principal investigator and answer questions that they may have. Participants who continue study drug in these circumstances may undergo repeat assessments consistent with careful clinical monitoring.

In HIV-infected subjects who develop a grade I-III clinical or laboratory toxicity the study team will provide the information to the primary clinician and will evaluate the toxicity on a case by case basis. The toxicity will be evaluated for subject safety and the subject will be removed from the study if the investigators or primary clinician believe the toxicity jeopardizes the study's intent and /or the study in so longer in the subject's best interest. HIV-infected subjects who

develop > grade III clinical or laboratory abnormality will be discontinued from the study and the primary clinician will be contacted to direct their care.

9.7 Criteria for Premature Study Discontinuation for an Individual Participant

Subjects who become pregnant on study will be discontinued from the study. Subjects who require an exclusionary medication during the study will be discontinued. Subjects who are deemed to be non-compliant with the study medications and/or procedures – as evidenced by undetectable drug concentrations, medication counts, medication diaries, and/or missed study visits – may be discontinued from the study at the discretion of the investigators. Once a subject is discontinued from the study, they may not re-enroll. Those subjects who do not finish at least day 30 will be replaced. It is estimated that 15% of subjects will not complete the study and will need to be replaced. It is estimated that most HIV-negative volunteers who finish 30 days on therapy will also finish the final 30 days off therapy, so that the terminal elimination half-life can still be estimated.

10. STATISTICAL CONSIDERATIONS

10.1 Overview and design issues

The primary goals of this study are as follows. The primary goal of Aim 1 is to comprehensively characterize and compare the cellular pharmacology of TFV/FTC in HIV-positive versus HIV-negative adults. Additionally, the intensive sampling early in the study will capture the accumulation of TFV-DP and FTC-TP as the concentrations rise to steady state and the sampling off drug will capture the terminal elimination phase in HIV-negative volunteers.

The primary goal of aim 2 is to test whether TFV inhibits natural PNP activity *in vivo*.

The primary goal of aim 3 is to identify a prophylactic threshold for intracellular TFV-DP and FTC-TP in PBMCs *in vivo.* Aim 3 is separate from aims 1 and 2, and the subjects who makeup aim 3 are different from those who participated in aims 1 and 2. Aim 3 will be assessed with stored PBMCs from the "Chemoprophylaxis for HIV infection in men (iPrEx)" protocol. iPrEx is an ongoing Phase III, randomized, double-blind, placebo-controlled trial of the safety and efficacy of chemoprophylactic TDF/FTC administered orally once daily to men at high risk for acquiring HIV-1. iPrEx is designed to enroll 3000 HIV seronegative high-risk men who have sex with men (MSM) who will be randomized to receive either TDF/FTC daily (n=1500) or TDF/FTC placebo daily (n=1500) in addition to standard counseling, condoms, and sexually transmitted infections management.

The primary goal for aim 4 is to develop a comprehensive pharmacokinetic model for TFV -DP and FTC-TP according to cell-type and tissue compartment with which to predict the optimal dose and onset and duration of prophylactic action.

A general overview of the study design and how the design addresses aims 1 and 2 is provided on the next page and in the schedule of events in Appendix A. The sampling for the rectal biopsy, genital tract, and extra-blood draw is best described in Appendix A and in sections 2 and 7. Of note, each subject will have one rectal biopsy, one genital sampling, and one extra blood draw for cell sorting. Four different HIV-negative and four different HIV-positive subjects will have each

of these procedures on each visit so as to balance the amount of these special pharmacology collection data at each study visit.

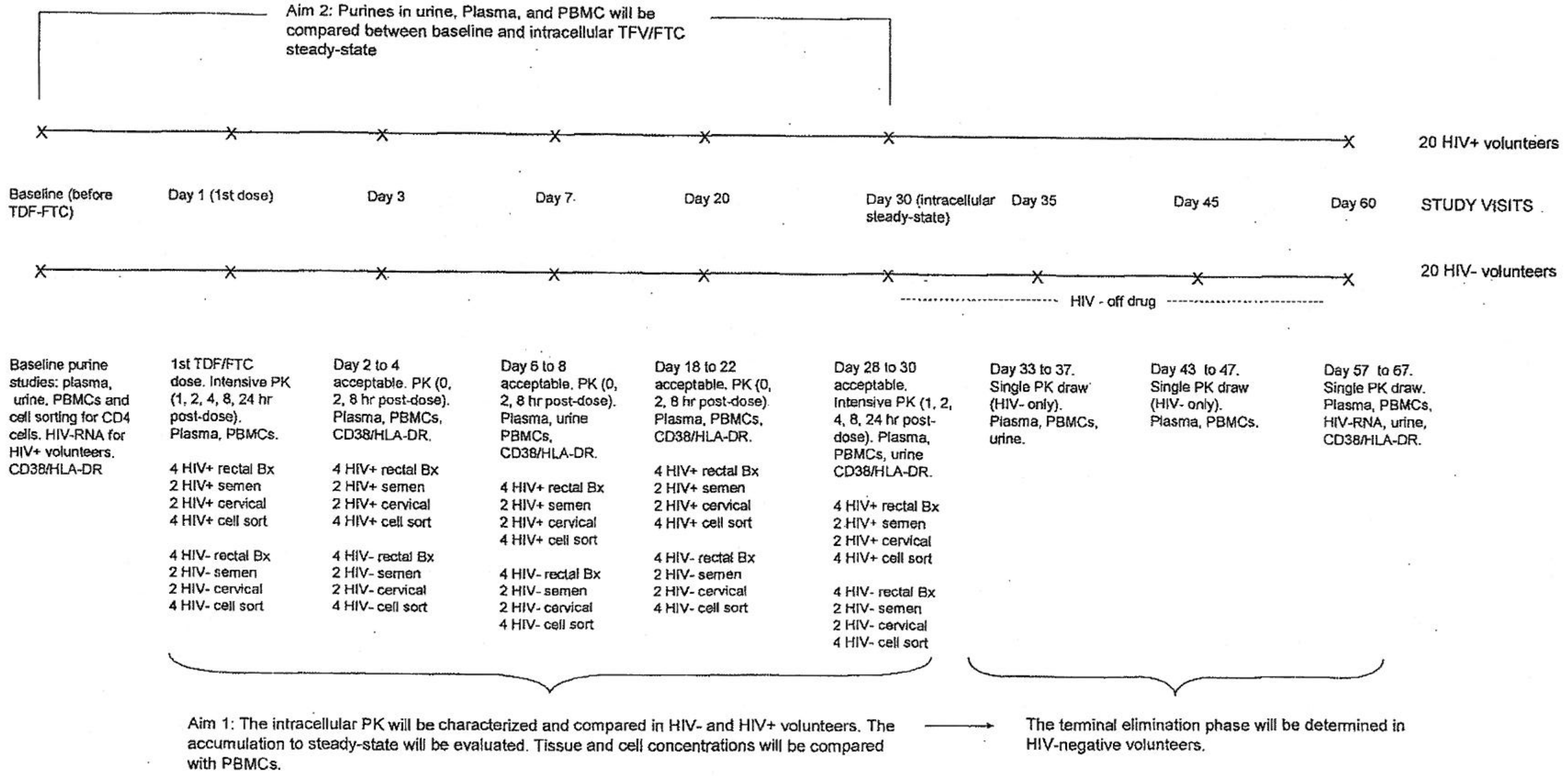
Aim 2: Purines in urine, Plasma, and PBMC will be compared between baseline and intracellular TFV/FTC steady-state
20 HIV+ volunteers
Baseline (before TDF-FTC)
Day 1 (1st dose)
Day 3
Day 7
Day 20
Day 30 (intracellular steady-state)
Day 35
Day 45
Day 60
STUDY VISITS
20 HIV- volunteers
HIV - off drug
Baseline purine studies: plasma, urine, PBMCs and cell sorting for CD4 cells. HIV-RNA for HIV+ volunteers. CD38/HLA-DR
1st TDF/FTC dose. Intensive PK (1, 2, 4, 8, 24 hr post-dose). Plasma, PBMCs.
4 HIV+ rectal Bx
2 HIV+ semen
2 HIV+ cervical
4 HIV+ cell sort
4 HIV- rectal Bx
2 HIV- semen
2 HIV- cervical
4 HIV- cell sort
Day 2 to 4 acceptable. PK (0, 2, 8 hr post-dose). Plasma, PBMCs, CD38/HLA-DR.
4 HIV+ rectal Bx
2 HIV+ semen
2 HIV+ cervical
4 HIV+ cell sort
4 HIV- rectal Bx
2 HIV- semen
2 HIV- cervical
4 HIV- cell sort
Day 6 to 8 acceptable. PK (0, 2, 8 hr post-dose). Plasma, urine PBMCs, CD38/HLA-DR.
4 HIV+ rectal Bx
2 HIV+ semen
2 HIV+ cervical
4 HIV+ cell sort
4 HIV- rectal Bx
2 HIV- semen
2 HIV- cervical
4 HIV- cell sort
Day 18 to 22 acceptable. PK (0, 2, 8 hr post-dose). Plasma, PBMCs, CD38/HLA-DR.
4 HIV+ rectal Bx
2 HIV+ semen
2 HIV+ cervical
4 HIV+ cell sort
4 HIV- rectal Bx
2 HIV- semen
2 HIV- cervical
4 HIV- cell sort
Day 28 to 30 acceptable. Intensive PK (1, 2, 4, 8, 24 hr post-dose). Plasma, PBMCs, urine CD38/HLA-DR.
4 HIV+ rectal Bx
2 HIV+ semen
2 HIV+ cervical
4 HIV+ cell sort
4 HIV- rectal Bx
2 HIV- semen
2 HIV- cervical
4 HIV- cell sort
Day 33 to 37. Single PK draw (HIV- only). Plasma, PBMCs, urine.
Day 43 to 47. Single PK draw (HIV- only). Plasma, PBMCs.
Day 57 to 67. Single PK draw. Plasma, PBMCs, HIV-RNA, urine, CD38/HLA-DR.
Aim 1: The intracellular PK will be characterized and compared in HIV- and HIV+ volunteers. The accumulation to steady-state will be evaluated. Tissue and cell concentrations will be compared with PBMCs.
The terminal elimination phase will be determined in HIV-negative volunteers.

10.2 Study endpoints

The study has four main endpoints:

1. A comparison of the first dose TFV-DP and FTC-TP AUC in HIV-negative versus HIV-positive adults.

2. A comparison of average steady-state plasma deoxyguanosine concentrations before therapy and after 30 days of TDF/FTC therapy.

3. A comparison of TFV-DP and FTC-TP in HIV-seroconverters versus matched non-seroconverters.

4. A pharmacokinetic model that describes the intracellular pharmacokinetics of TFV-DP and FTC-TP in PBMCs so that various dosing strategies can be tested on the model to identify the optimal dosing that most rapidly achieves and sustains the desired prophylactic threshold for HIV prevention.

10.3 Sample Size Considerations

The overall sample size was chosen based on aim 1 to detect a ~60% increase (63%) in TFV-DP in HIV-negative versus HIV-infected persons using a significance level of 0.05, a power of 80%, a coefficient of variation for TFV-DP of 50%, and a two-sample t-test.(37) The rationale for 60% is that in vitro studies suggest ~2.5-fold higher TFV-DP in resting cells.(10) We chose a quarter of that value because increased cell activation from HIV in patients is not of the same magnitude as increased cell activation from PHA-stimulation in vitro.(46, 47)

Aim 2. Baseline (pre-drug) and day 30 (steady-state for intracellular TFV/FTC) purine concentrations in urine, plasma, and intracellularly will be compared with paired tests. The potency of TFV-MP inhibition of ddI breakdown by PNP (~35nM) is the same as BCX-34 inhibition for natural purines (BCX-34 is a PNP inhibitor no longer in development).(48) BCX-34 changed plasma purines by 3-fold. If TFV-MP induces the same change as BCX-34 in purines in urine/plasma/or cells, we will have >99% power to detect such a difference between baseline and day 30 in each serostatus cohort of 20.

Aim 3. If we assume 24 sero-conversions in the active drug group of iPrEx, which is the actual assumption for the iPrEx study design, and we obtain samples from those 24 subjects and from 96 matched control subjects from the active drug arm, then we have 92% power to detect a difference of 50% in the intracellular concentration of TFV-DP assuming a constant coefficient of variation of 50%. With 1:4 case:control matching, the analysis will have > 87% power for 20 or more sero-conversions in the active drug group with the same assumptions.

Aim 4. We will develop a NONMEM model to simultaneously assess all the concentrations gathered in this study (including from the iPrEx study) and to test whether HIV sero-status, sex, race, cell activation, cell type, etc explains observed variability. NONMEM methodology is not amenable to traditional sample size calculations. The present study will analyze about 900 NRTI-triphosphate samples for modeling. Our previous studies have modeled about 300 NRTI-

triphosphate samples and have successfully drawn conclusions regarding covariates from these data.(49)

10.4 Enrollment/Stratification/Randomization/Blinding Procedures

This is an open-label, non-randomized, observational study. There is no blinding. Subjects will be enrolled sequentially with the goal of balanced race and sex stratification when possible as shown in the table below. Note that 40 subjects are sought to complete the study as shown in the table, but COMIRB-approval will be for 60 subjects to allow for screen-disqualifications and drop-outs.

	HIV-negative		HIV-infected,	
	men	women	men	women
African-American	5	5	5	5
Non-African-American	5	5	5	5

The enrollment into the special pharmacology procedures will occur as follows. There will be five possible sequences of special pharmacology samplings (rectal biopsy, genital sampling, and extra blood draw); A, B, C, D, and E, as defined in the following table.

	Study Visits				
Sequence	Day 1	Day 3 (2 to 4)	Day 7 (6 to 8)	Day 20 (18 to 22)	Day 30 (28 to 32)
A	Rectal	-	-	Blood	Genital
B	Genital	Rectal	-	-	Blood
C	Blood	Genital	Rectal	-	-
D	-	Blood	Genital	Rectal	-
E	-	-	Blood	Genital	Rectal

An excel spreadsheet will be created with four columns of 10 entries of A, B, C, D, and E (2 of each) in random order. One column will be for HIV-negative men, one for HIV-negative women, one for HIV-positive men, and the last for HIV-positive women. Subjects will be sequentially assigned to a sequence (A, B, C, D, or E) from this spreadsheet. Should a subject not be able to accommodate the sequence to which they were assigned (for example, because of menstruation), they will be assigned to the next unique sequence in the order. Then the following subject will be assigned to the previous sequence.

10.5 Participant Enrollment Follow-Up

Enrollment will be tracked at bi-weekly investigator meetings and every 6 months at the safety officer meetings.

10.6 Data and Safety Monitoring

The data and safety monitoring will be conducted by the investigators on an ongoing basis during the study according to standard operating procedures, at bi-weekly investigator meetings, and at safety officer meetings, as outlined in the manual of operations. Data and safety will be reported to the safety officer and DAIDS monitors.

10.7 Planned Interim Analyses and Stopping Guidelines

Because of the study is non-therapeutic and relatively small (N=40) with a short duration of the intervention (60 days), the study team will monitor for safety, but not efficacy. Stopping guidelines have not been developed for efficacy.

10.8 Safety Review

Safety will be reviewed during the safety officer meetings every six months as outlined in the safety officer charter in the appendix.

10.9 Analysis Plan

The study statistician, Dr. Sam MaWhinney, will oversee the data analyses. Aim 1 will compare the first dose TFV-DP and FTC-TP AUC in HIV-negative versus HIV-positive adults. Parametric tests will be used for these analyses. Data will be transformed to provide a normal distribution, as needed. Aim 2 will compare average steady-state plasma deoxyguanosine concentrations before therapy and after 30 days of TDF/FTC therapy. Paired parametric tests will be used for these analyses. Aim 3 will evaluate the probability of seroconversion for various drug levels within each case-control matching stratification using conditional logistic regression. In the presence of a prophylactic drug level threshold, we anticipate a nonlinear relationship between drug level and the probability of seroconversion. It is also natural to estimate receiver operating characteristics (ROC) curves from a case-control study as sensitivity and specificity are defined by seroconverter status. The ROC allows us to examine the use of drug level to classify seroconverters and controls. In addition, the ROC provides an estimate of the optimal concentration (threshold) for maximum sensitivity and specificity. For aim 4, a base NONMEM model with no covariates will be created on the basis of residual plots and the lowest objective function. The predictors will be tested on TFV-DP and FTC-TP CL (and therefore AUC); each predictor will be added to the model individually, in a nested approach, and a statistical significance level of 0.1 will be used to determine what stays in the model. When all significant predictors are determined, a backward selection approach will be used to test for co-linearity.

11. DATA HANDLING AND RECORDKEEPING

11.1 Data Management Responsibilities

Data will be managed by study personnel. Data will be accessed and centralized on the School of Pharmacy, password protected server. System maintenance is performed on a daily basis by the

University of Colorado IT Department. All relevant personnel will go through data collection training. Data will be stored both in hard copy and electronic format. Hard copies are kept in a locked cabinet to ensure privacy, confidentiality and security. Two SOPs (#028 and #029) have been developed for data collection, reporting, storage, access, entry and maintenance. A master log of all data to be entered will be created. A quality control database checklist for data entry will be used and a system is in place to check for accuracy.

11.2 Source Documents and Access to Source Data/Documents

Source documents will be kept in subject study records (binders). Documentation will be handled as detailed in SOP #008 so that study data can be reconstructed, evaluated, and validated for all clinical activities during the trial. The goals are to ensure that protocol, hospital, COMIRB, and DAIDS requirements and standards are adhered to and that all data will be verifiable from the written source document and will create an audit trail to verify that data is present, complete and accurate. Source data consists of all information in original records and certified copies of original records. The "ALCOA" method is used to achieve and maintain data quality: Attributable, Legible, Contemporaneous, Original and Accurate.

11.3 Quality Control and Quality Assurance

A program for quality assurance and quality control has been developed as a system of self monitoring to promote the integrity and quality of the study. The program is detailed in SOP #030. Other quality management approaches for this study include the regulatory review tool provided by DAIDS, which will be used for quarterly review of regulatory documents as required by DAIDS, and a good clinical practices (GCP) checklist (detailed in SOP #010). The Principal Investigator will prepare an annual evaluation and summary of the QA/QC program and its activities to be submitted to DAIDS utilizing the DAIDS-specific format. The results from the quality assurance-quality improvement program will be available to reviewers at site monitoring visits.

12. CLINICAL SITE MONITORING

This study will be monitored continuously by study personnel as outlined in sections 10 and 11. Additionally, an external site monitoring program was developed to ensure consistency, completeness, and validity of research data, safety of the participants, and compliance with the protocol and all regulatory laws and requirements.

12.1 External Auditing

This study is a cooperative agreement with DAIDS. A DAIDS monitor will be assigned to monitor and audit the study. The Principal Investigator will be the responsible study site official and the study coordinator will be available to provide documents and to answer questions for the audits. DAIDS staff will determine frequency of the visits based on the risk, size, and complexity of the study. DAIDS may change the degree and frequency of monitoring at their discretion. The goals of the monitoring are to maximize adherence in the conduct of the trial with applicable regulations, policies, standard procedures, required guidelines and study protocols and to verify data quality, completeness and accuracy.

The DAIDS site monitor will conduct on site reviews of source documents, participant records, regulatory files, facilities, laboratories and the dispensing pharmacy. The signed informed consent documents will be made available for review for compliance with GCP and the DAIDS policy requirements. Original source documents will be made available to verify all inclusion/exclusion criteria and for compliance with protocol requirements and DAIDS policies for source documentation. Individual participant's source documents will also be available for review and comparison to protocol requirements and the completed case report forms (CRFs). The number of records to be reviewed will be decided by the DAIDS monitor based on a number of factors including study risk, size, complexity, research staff experience and prior findings.

The regulatory file will be made available for review by the DAIDS site monitor. This is an essential document for the trial and will follow the DAIDS requirements. A site monitoring visit log will be maintained to track all visits made. A dated signature of the monitor for each study visit will be kept in the log.

12.2 Site Visit Summary Meeting

Prior to leaving the site visit, the DAIDS site monitor will meet with the Principal Investigator and study coordinator at an agreed upon time and place to review the findings from the visit. Requests for corrective action may be made by the DAIDS monitor at this meeting.

12.3 The Site Monitoring Report

A written detailed report of the DAIDS site monitor's reviews of records, regulatory files, and site and pharmacy operations will be completed by the DAIDS monitor within 15 working days after the visit. This report will be distributed to appropriate DAIDS staff and the Principal Investigator. Critical issues or findings will be expedited accordingly.

12.4 Review and Follow up of Monitoring Report Findings

A plan has been developed for the review and follow up of DAIDS Site Monitoring Reports. The details are provided in SOP #034. The plan includes timely follow up and correction of deficiencies identified during the visit. The Principal Investigator will be responsible for corrective action plan and will keep records of such actions.

13. HUMAN SUBJECTS PROTECTION

13.1 Institutional Review Board/Ethics Committee

The University of Colorado holds a current U.S. Federal-Wide Assurance issued by the Office for Human Research Protections (OHRP). The Colorado Multiple Institutional Review Board (COMIRB) is responsible for assuring that this protocol and the associated informed consent documents and study-related documents are reviewed prior to implementation of the protocol. All amendments to the protocol, informed consent and other study related documents are approved by COMIRB prior to implementation. An annual continuing review process is required and the study may not continue without this annual approval.

13.2 Protocol Registration

Prior to implementation of this protocol, and any subsequent full version amendments, each site must have the protocol and the protocol informed consent form approved, as appropriate, by their local institutional review board (IRB) and any other applicable regulatory entity (RE). Upon receiving final approval, sites will submit all required protocol registration documents to the DAIDS Protocol Registration Office (DAIDS PRO) at the Regulatory Support Center (RSC). The DAIDS PRO will review the submitted protocol registration packet to ensure that all of the required documents have been received.

Site-specific informed consent forms (ICFs) will be reviewed and approved by the DAIDS PRO and sites will receive an Initial Registration Notification from the DAIDS PRO that indicates successful completion of the protocol registration process. A copy of the Initial Registration Notification should be retained in the site's regulatory files.

Upon receiving final IRB and any other applicable RE approval for an amendment, sites should implement the amendment immediately. Sites are required to submit an amendment registration packet to the DAIDS PRO at the RSC. The DAIDS PRO will review the submitted protocol registration packet to ensure that all the required documents have been received. Site-specific ICFs will not be reviewed and approved by the DAIDS PRO and sites will receive an Amendment Registration Notification when the DAIDS PRO receives a complete registration packet. A copy of the Amendment Registration Notification should be retained in the site's regulatory files.

For additional information on the protocol registration process and specific documents required for initial and amendment registrations, refer to the current version of the DAIDS Protocol Registration Manual.

Protocol registration will occur before any subjects are enrolled into the study.

13.3 Informed Consent Process

In obtaining and documenting informed consent, the investigators will comply with all applicable local and/or domestic regulatory requirements and will adhere to Good Clinical Practices and to all ethical principles from the Declaration of Helsinki. Written documentation of informed consent approval will be present prior to initiation of any study related procedures. Subjects will be given a copy of the signed consent document. At each study visit, study personnel and the subject will reaffirm the subject's understanding of the research and their desire to continue. This is documented on a case report form and kept in the subject's research record.

13.4 Participant Confidentiality

This study will test all subjects for gonorrhea and chlamydia and HIV-negative volunteers for HIV and hepatitis B virus (HBV) infection. A positive HIV or HBV result on the screen would render the subject ineligible for the study and would require reporting to the Colorado Department of Health. A study physician from the protocol will counsel the subject about confirmatory tests, as needed, and will refer the subject to appropriate follow up care. A positive test for gonorrhea and /or chlamydia must also be reported to the Colorado Department of Health by law. The tests are also posted to the electronic medical record.

In order to strictly protect confidentiality, study records will not be released to insurance companies or any other unauthorized parties without the subject's written consent. COMIRB, DAIDS/NIAID, OHRP, Gilead Sciences and the FDA are entities that may review participants' records. The data management plan for this protocol will separate all of the 18 HIPAA protected health identifiers from the electronic research database, which will identify subjects with only their PID. The electronic research database will be password protected and backed-up on secure servers on the University of Colorado Denver information data networks. Recover and restoration of data is covered under the terms of the University of Colorado Denver Information Services Department. Paper study records, which include the protected health identifiers, will be kept in binders in a locked cabinet in a locked office.

This study will also evaluate relationships between genetic variability in enzymes responsible for tenofovir and emtricitabine disposition and drug response. There is a very remote risk that an insurance company or third party could access DNA information and use it to discriminate against subjects. Risks associated with DNA samples will be minimized by storing genetic material in a locked laboratory. Genetic samples will be labeled with a coded number; it will not identify subjects by name or other identifying information. Results of genetic analyses will not be included in the medical record and will be kept in a locked file cabinet that is available only to the study investigators. If a subject decides to withdraw their sample, they may contact Dr. Anderson in writing and their samples will be withdrawn and destroyed. SOP # 012 has been developed to detail the approach for maintaining subject confidentiality.
To help the research team add additional privacy protections for the research subjects, a Certificate of Confidentiality will be obtained from the Food and Drug Administration. With this Certificate, the researchers cannot be forced to disclose information that may identify subjects, even by a court subpoena, in any federal, state, or local civil, criminal, administrative, legislative, or other proceedings.

13.5 Study Discontinuation

The study may be discontinued at any time by COMIRB, DAIDS/NIAID, OHRP, the FDA, or other government agencies as part of their duties to ensure that research subjects are protected.

14. PUBLICATION POLICY

The Principal Investigator will have oversight of publications and presentations emanating from this study. The Principal Investigator will assure that authorship and authorship order is equitable with respect to the effort contributed to the work. Contributing effort that justifies authorship includes participation in the design of the study and assays used in the study; participation in carrying out the study; participation in analyzing the data; and participation in writing the manuscript or presentation. Trainees or students who contribute work will be equally eligible for authorship. First authorship will reflect the individual who contributes the most effort and writing to the manuscript. First authorship may be split if two individuals contributed to the effort equally. Senior authorship will reflect the individual who provided the scientific oversight and direction for the manuscript or presentation.

15. REFERENCES

1. Kakuda TN. Pharmacology of nucleoside and nucleotide reverse transcriptase inhibitor-induced mitochondrial toxicity. Clin Ther. 2000 Jun;22(6):685-708.
2. Anderson PL, Kakuda TN, Lichtenstein KA. The cellular pharmacology of nucleoside- and nucleotide-analogue reverse-transcriptase inhibitors and its relationship to clinical toxicities. Clin Infect Dis. 2004 Mar 1;38(5):743-53.
3. Furman PA, Fyfe JA, St Clair MH, Weinhold K, Rideout JL, Freeman GA, et al. Phosphorylation of 3'-azido-3'-deoxythymidine and selective interaction of the 5'-triphosphate with human immunodeficiency virus reverse transcriptase. Proc Natl Acad Sci U S A. 1986 Nov;83(21):8333-7.
4. Ray AS, Olson L, Fridland A. Role of purine nucleoside phosphorylase in interactions between 2',3'-dideoxyinosine and allopurinol, ganciclovir, or tenofovir. Antimicrob Agents Chemother. 2004 Apr;48(4):1089-95.
5. Bantia S, Ananth SL, Parker CD, Horn LL, Upshaw R. Mechanism of inhibition of T-acute lymphoblastic leukemia cells by PNP inhibitor--BCX-1777. Int Immunopharmacol. 2003 Jun;3(6):879-87.
6. Vela JE, Miller MD, Rhodes GR, Ray AS. Effect of nucleoside and nucleotide reverse transcriptase inhibitors of HIV on endogenous nucleotide pools. Antivir Ther. 2008;13(6):789-97.
7. Gao WY, Shirasaka T, Johns DG, Broder S, Mitsuya H. Differential phosphorylation of azidothymidine, dideoxycytidine, and dideoxyinosine in resting and activated peripheral blood mononuclear cells. J Clin Invest. 1993 May;91(5):2326-33.
8. Gao WY, Agbaria R, Driscoll JS, Mitsuya H. Divergent anti-human immunodeficiency virus activity and anabolic phosphorylation of 2',3'-dideoxynucleoside analogs in resting and activated human cells. J Biol Chem. 1994 Apr 29;269(17):12633-8.
9. Balzarini J, Van Herrewege Y, Vanham G. Metabolic activation of nucleoside and nucleotide reverse transcriptase inhibitors in dendritic and Langerhans cells. AIDS. 2002 Nov 8;16(16):2159-63.
10. Robbins BL, Srinivas RV, Kim C, Bischofberger N, Fridland A. Anti-human immunodeficiency virus activity and cellular metabolism of a potential prodrug of the acyclic nucleoside phosphonate 9-R-(2-phosphonomethoxypropyl)adenine (PMPA), Bis(isopropyloxymethylcarbonyl)PMPA. Antimicrob Agents Chemother. 1998 Mar;42(3):612-7.
11. Back DJ, Burger DM, Flexner CW, Gerber JG. The pharmacology of antiretroviral nucleoside and nucleotide reverse transcriptase inhibitors: implications for once-daily dosing. J Acquir Immune Defic Syndr. 2005 Aug 1;39 Suppl 1:S1-23, quiz S4-5.
12. Hestdal K, Aukrust P, Muller F, Lien E, Bjerkeli V, Espevik T, et al. Dysregulation of membrane-bound tumor necrosis factor-alpha and tumor necrosis factor receptors on mononuclear cells in human immunodeficiency virus type 1 infection: low percentage of p75-tumor necrosis factor receptor positive cells in patients with advanced disease and high viral load. Blood. 1997 Oct 1;90(7):2670-9.
13. Behbahani H, Landay A, Patterson BK, Jones P, Pottage J, Agnoli M, et al. Normalization of immune activation in lymphoid tissue following highly active antiretroviral therapy. J Acquir Immune Defic Syndr. 2000 Oct 1;25(2):150-6.
14. Brannstrom M, Friden BE, Jasper M, Norman RJ. Variations in peripheral blood levels of immunoreactive tumor necrosis factor alpha (TNFalpha) throughout the menstrual cycle and secretion of TNFalpha from the human corpus luteum. Eur J Obstet Gynecol Reprod Biol. 1999 Apr;83(2):213-7.

15. Godfried MH, van der Poll T, Jansen J, Romijin JA, Schattenkerk JK, Endert E, et al. Soluble receptors for tumour necrosis factor: a putative marker of disease progression in HIV infection. AIDS. 1993 Jan;7(1):33-6.
16. Barry M, Wild M, Veal G, Back D, Breckenridge A, Fox R, et al. Zidovudine phosphorylation in HIV-infected patients and seronegative volunteers. AIDS. 1994 Aug;8(8):F1-5.
17. Barry MG, Khoo SH, Veal GJ, Hoggard PG, Gibbons SE, Wilkins EG, et al. The effect of zidovudine dose on the formation of intracellular phosphorylated metabolites. AIDS. 1996 Oct;10(12):1361-7.
18. Wattanagoon Y, Na Bangchang K, Hoggard PG, Khoo SH, Gibbons SE, Phiboonbhanakit D, et al. Pharmacokinetics of zidovudine phosphorylation in human immunodeficiency virus-positive thai patients and healthy volunteers. Antimicrob Agents Chemother. 2000 Jul;44(7):1986-9.
19. CDC. CDC Trials of Pre-exposure Prophylaxis for HIV Prevention. Available at: http://www.cdc.gov/hiv/resources/factsheets/PDF/prep.pdf. Accessed: 11-12-2008.
20. Mayer KH, Mimiaga MJ, Cohen D, Grasso C, Bill R, Van Derwarker R, et al. Tenofovir DF plus lamivudine or emtricitabine for nonoccupational postexposure prophylaxis (NPEP) in a Boston Community Health Center. J Acquir Immune Defic Syndr. 2008 Apr 1;47(4):494-9.
21. Chi BH, Chintu N, Cantrell RA, Kankasa C, Kruse G, Mbewe F, et al. Addition of single-dose tenofovir and emtricitabine to intrapartum nevirapine to reduce perinatal HIV transmission. J Acquir Immune Defic Syndr. 2008 Jun 1;48(2):220-3.
22. Vernazza PL, Hollander L, Semprini AE, Anderson DJ, Duerr A. HIV-discordant couples and parenthood: how are we dealing with the risk of transmission? AIDS. 2006 Feb 28;20(4):635-6.
23. Frascino R. Ask the experts forum on The Body website. April 6, 2006. Available at: http://www.thebody.com/Forums/AIDS/Fatigue/Current/Q173403.html?h65h Last Accessed January 5, 2009.
24. Cohen MS, Gay C, Kashuba AD, Blower S, Paxton L. Narrative review: antiretroviral therapy to prevent the sexual transmission of HIV-1. Ann Intern Med. 2007 Apr 17;146(8):591-601.
25. Nelson MR, Katlama C, Montaner JS, Cooper DA, Gazzard B, Clotet B, et al. The safety of tenofovir disoproxil fumarate for the treatment of HIV infection in adults: the first 4 years. AIDS. 2007 Jun 19;21(10):1273-81.
26. Garcia-Lerma J, Cong M, Masciotra S, Youngpairoj A, Mitchell J, Hanson D, et al. Intermittent Pre-Exposure Prophylaxis (Prep) with Oral Truvada Protects Macaques against Repeated Rectal SHIV Exposures [abstract 40]. 3rd International Workshop on HIV Transmission; July 31- August 2, 2008; Mexico City, Mexico.
27. Garcia-Lerma JG, Otten RA, Qari SH, Jackson E, Cong ME, Masciotra S, et al. Prevention of rectal SHIV transmission in macaques by daily or intermittent prophylaxis with emtricitabine and tenofovir. PLoS Med. 2008 Feb;5(2):e28.
28. Cranage M, Sharpe S, Herrera C, Cope A, Dennis M, Berry N, et al. Prevention of SIV rectal transmission and priming of T cell responses in macaques after local pre-exposure application of tenofovir gel. PLoS Med. 2008 Aug 5;5(8):e157; discussion e.
29. Denton PW, Estes JD, Sun Z, Othieno FA, Wei BL, Wege AK, et al. Antiretroviral pre-exposure prophylaxis prevents vaginal transmission of HIV-1 in humanized BLT mice. PLoS Med. 2008 Jan 15;5(1):e16.
30. Van Rompay KK, Berardi CJ, Aguirre NL, Bischofberger N, Lietman PS, Pedersen NC, et al. Two doses of PMPA protect newborn macaques against oral simian immunodeficiency virus infection. AIDS. 1998 Jun 18;12(9):F79-83.

31. Van Rompay KK, Marthas ML, Lifson JD, Berardi CJ, Vasquez GM, Agatep E, et al. Administration of 9-[2-(phosphonomethoxy)propyl]adenine (PMPA) for prevention of perinatal simian immunodeficiency virus infection in rhesus macaques. AIDS Res Hum Retroviruses. 1998 Jun 10;14(9):761-73.
32. Van Rompay KK, McChesney MB, Aguirre NL, Schmidt KA, Bischofberger N, Marthas ML. Two low doses of tenofovir protect newborn macaques against oral simian immunodeficiency virus infection. J Infect Dis. 2001 Aug 15;184(4):429-38.
33. Tsai CC, Follis KE, Sabo A, Beck TW, Grant RF, Bischofberger N, et al. Prevention of SIV infection in macaques by (R)-9-(2-phosphonylmethoxypropyl)adenine. Science. 1995 Nov 17;270(5239):1197-9.
34. Wang LH, Begley J, Feng JY, Quinn J, Rousseau F. Pharmacokinetic and pharmacodynamic characteristics of emtricitabine support its once daily dosing [abstract 4546]. Program and abstracts of the XIV International AIDS Conference. International AIDS Society. Barcelona, Spain, 2002
35. Hawkins T, Veikley W, St Claire RL, 3rd, Guyer B, Clark N, Kearney BP. Intracellular pharmacokinetics of tenofovir diphosphate, carbovir triphosphate, and lamivudine triphosphate in patients receiving triple-nucleoside regimens. J Acquir Immune Defic Syndr. 2005 Aug 1;39(4):406-11.
36. Lockman S, Shapiro RL, Smeaton LM, Wester C, Thior I, Stevens L, et al. Response to antiretroviral therapy after a single, peripartum dose of nevirapine. N Engl J Med. 2007 Jan 11;356(2):135-47.
37. Kiser JJ, Aquilante CL, Anderson PL, King TM, Carten ML, Fletcher CV. Clinical and genetic determinants of intracellular tenofovir diphosphate concentrations in HIV-infected patients. J Acquir Immune Defic Syndr. 2008 Mar 1;47(3):298-303.
38. Department of health and human services/Henry J. Kaiser family foundation guidelines for the use of antiretroviral agents in HIV-infected adults and adolescents. Panel on Clinical Practices for the Treatment of HIV Infection. Living document last updated November 2008 (available at AIDSinfo.NIH.gov). Accessed 11/12/08.
39. Saag MS. Emtricitabine, a new antiretroviral agent with activity against HIV and hepatitis B virus. Clin Infect Dis. 2006 Jan 1;42(1):126-31.
40. Truvada® (tenofovir disoproxil fumarate and emtricitabine). Product Information. May 2008; Gilead Sciences, Foster City, CA.
41. Peterson L, Taylor D, Roddy R, Belai G, Phillips P, Nanda K, et al. Tenofovir disoproxil fumarate for prevention of HIV infection in women: a phase 2, double-blind, randomized, placebo-controlled trial. PLoS Clin Trials. 2007;2(5):e27.
42. Shacklett BL, Yang O, Hausner MA, Elliott J, Hultin L, Price C, et al. Optimization of methods to assess human mucosal T-cell responses to HIV infection. J Immunol Methods. 2003 Aug;279(1-2):17-31.
43. Dumond JB, Reddy YS, Troiani L, Rodriguez JF, Bridges AS, Fiscus SA, et al. Differential extracellular and intracellular concentrations of zidovudine and lamivudine in semen and plasma of HIV-1-infected men. J Acquir Immune Defic Syndr. 2008 Jun 1;48(2):156-62.
44. Division of AIDS. Division of AIDS table for grading severity of adult and pediatric adverse events. National Institutes of Allergy and Infectious Disease, Division of AIDS; December 2004 (clarification dated August 2009)
45. Atripla® (efavirenz/emtricitabine/tenofovir disoproxil fumarate). Bristol-Myers Squibb and Gilead Sciences. Sept 2008.
46. Heinschink A, Raab M, Daxecker H, Griesmacher A, Muller MM. In vitro effects of mycophenolic acid on cell cycle and activation of human lymphocytes. Clin Chim Acta. 2000 Oct;300(1-2):23-8.

47. Mahalingam M, Pozniak A, McManus TJ, Vergani D, Peakman M. Cell cycling in HIV infection: analysis of in vivo activated lymphocytes. Clin Exp Immunol. 1995 Dec;102(3):481-6.
48. Viegas TX, Omura GA, Stoltz RR, Kisicki J. Pharmacokinetics and pharmacodynamics of peldesine (BCX-34), a purine nucleoside phosphorylase inhibitor, following single and multiple oral doses in healthy volunteers. J Clin Pharmacol. 2000 Apr;40(4):410-20.
49. Anderson PL, Kakuda TN, Kawle S, Fletcher CV. Antiviral dynamics and sex differences of zidovudine and lamivudine triphosphate concentrations in HIV-infected individuals. AIDS. 2003 Oct 17;17(15):2159-68.

16. APPENDICES

Appendix A: Schedule of Events

Appendix B: Safety officer charter

Appendix C: Informed Consents

NOTED
JAN 29 2013
COMIRB

Schedule of Events:

	Screen[a]	Baseline[e]	Day 1	Day 3 (2 to 4)	Day 7 (6 to 8)	Day 20 (18 to 22)	Day 30 (28 to 32)	Day 35[c] (33 to 37)	Day 45[c] (43 to 47)	Day 60 (57 to 67)
SAFETY ASSESSMENTS										
UA	X+ alb/cr			X	X	X	X	PRN	PRN	PRN[c], X (HIV+)
CMP +, phos, & lipase	X			X	X	X	X	PRN	PRN	PRN[c], X (HIV+)
CBC Hematology (HG, HCT, WBCs, PLTs)	X			X	X	X	X	PRN	PRN	PRN[c], X (HIV+)
Pregnancy	X	PRN	PRN	PRN	PRN	PRN	PRN[c] X (HIV+)	PRN	PRN	PRN[c], X (HIV+)
HIV test (HIV- only)	X									
HBV test (HIV- only)	X									
Medical Hx	X									
Physical exam	X									
Medication Hx	X	X	X	X	X	X	X	X	X	X
Medication diary			X	X	X	X	X			X (HIV+)only
Pill count			X	X	X	X	X			X (HIV+)only
Adverse effect questionnaire			X	X	X	X	X	X	X	X
HIV-RNA (HIV+ only)	X									X
Mouthwash sample		X								
ROUTINE PHARMACOLOGY ASSESSMENTS										
PBMCs		X	X	X	X	X	X	X	X	X
Plasma		X	X	X	X	X	X	X	X	X
Stored PBMC		X			X		X	X		X
Urine purines[b]		X			X		X	X		X
CD38/ HLADR		X		X	X	X	X			X
SPECIAL PHARMACOLOGY ASSESSMENTS (see footnotes)										
Rectal			A	B	C	D	E			
Genital[f]			B	C	D	E	A			
CD4 T[d]		X	C	D	E	A	B			
APPROXIMATE BLOOD VOLUME	25	60	55	47	57	47	79	32	11	38

Approximate maximum blood volume per subject = 450 to 480 mL

PRN = only if there is a clinical indication
[a] the consenting will take place before anything else on the screen visit. The screen and day 1 visits must be 21 or fewer days apart.
[b] Urine purines will be measured in spot urine samples.
[c] for HIV negative volunteers
[d] this will be an extra blood draw per subject of 32 mL for HIV-negative and 40 mL for HIV positive for cell purification
A = 4 HIV+ subjects and 4 HIV- subjects
B = a different 4 HIV+ subjects and 4 HIV- subjects
C = another different 4 HIV+ subjects and 4 HIV- subjects
D = another different 4 HIV+ subjects and 4 HIV- subjects
E = another different 4 HIV+ subjects and 4 HIV- subjects
[e] The Baseline and Day 1 visits should be 7 or fewer days apart.
[f] Subjects will be tested for sexually transmitted diseases at this visit (gonorrhea, Chlamydia, trichomonas) and yeast and bacterial vaginosis in women. This testing is done in a urine sample for men

Safety Officer Charter

Study Title: Cellular Pharmacology of tenofovir and emtricitabine for HIV Prophylaxis

Principal Investigator: Peter L. Anderson, Pharm.D.

NOTED

Study Sponsor: DAIDS, NIAID, NIH

JAN 29 2013

COMIRB

Safety Officer:

Name: Dr. Cara Wilson, M.D.

Past research and/or experience: Dr. Wilson is Associate Professor of Medicine, jointly appointed in the divisions of Infectious Diseases and Clinical Immunology at UCD. She also has a joint appointment in the Integrated Department of Immunology at National Jewish Medical Research Center and UCD. Dr. Wilson is a R01-funded investigator with experience in translational and clinical immunology. She currently serves as the chair of the Translational Research and Drug Development (TRADD) committee of the national AIDS Clinical Trials group, a committee that oversees all aspects of phase I and II AIDS-related clinical trials including studies of new antiretroviral drugs, pharmacokinetics , and immune-based therapy trials. She is a clinical investigator in the Colorado AIDS Clinical Trials unit and has experience with AIDS-related clinical trials at the local and national levels. Dr. Wilson holds a K24 award, and serves on the NIH NIAID AIDS Research Review Committee (RCC) study section that reviews career development grants. She serves on the advisory board for the UCD K12 Clinical Oncology Research Program, and holds graduate faculty status for graduate programs in the Department of Immunology, the Medical Scientist Training Program, and the Biomedical Sciences Program at UCD.

Potential Conflicts of Interest: None

Charter Description:

Julie Predhomme, N.P. (the study coordinator), and Drs. Anderson, Meditz (the study physician), and Wilson will meet every 6 months from the enrollment of the first subject, or December 1, 2009, whichever occurs first. Additional meetings will continue at 6 month intervals until the further enrollment and follow-up are closed. Any investigator on the protocol or the Research Subject Advocate may call unscheduled meetings at any time to discuss any concerns with the study (discussed below). Five working days prior to meetings, Dr. Anderson will be responsible for creating a confidential report for Dr. Wilson to review. Dr. Wilson may ask Dr. Anderson for any additional information she deems necessary for the meeting. Report data will be frozen about one week from the meeting. Based on the report, Dr. Wilson may request that other study investigators and/or the sponsor (delegates of DAIDS) attend the meeting. Data will be identified with a coded number; not personal identifiers. The content of the report and purpose of the meetings will be twofold. First to assess the safety of the study and second to review enrollment.

Enrollment:

We will focus on the enrollment of the HIV-negative group first.

Enrollment Goal (Years 1 and 2): ≥ 2 HIV-negative subjects per month.

We will focus on the HIV-positive group after addressing HIV-negative enrollment. We may begin enrolling HIV positive subjects in the first year if we are meeting or exceeding the enrollment goal for our HIV-negative cohort.

Enrollment Goal (Years 1, 2, and 3): ≥ 1 HIV-positive subject per month.

Subjects will be considered "retained" if they finish the Day 30. The number of subjects who are not retained will be reported to Dr. Wilson along with the reason for the drop-out. The plan is to replace dropouts, as to finish the study with 20 subjects in each group balanced by race and African-American versus non-African-American race if possible. The goal is 85% retention of subjects who start the study.

Dr. Wilson will receive enrollment and retention data for each meeting report. If these enrollment and retention goals are not met in two successive meetings, Drs. Wilson and Anderson will create a plan of action. Action items may include:

- Aggressive advertising via flyer and e-mail postings on the University of Colorado Denver Anschutz Medical and Downtown Campuses.
- Advertising on the CTRC website for clinical studies.
- Advertising in local newspapers (including on-line ads) serving African-American communities such as The Aurora Sentinel, The Denver Post, and The Westword including specification that African-Americans are especially needed for the study.
- Contacting potential subjects including African-Americans enrolled in previous studies conducted by our research group. For example, there are approximately a dozen African-American men and a dozen African-American women who have given prior written approval in the IRB-approved informed consent to be contacted for future research by our study team.
- Increase or requests that our off-site co-investigator, Dr. Gardner, an attending physician at Denver Health, refer patients to this study. This includes aggressive communication with providers at Children's Hospital CHIP clinic that sees young adults ages 18-21, Kaiser Clinic, National Jewish Clinic, and the Veterans Administration.
- As we near the mid-point of year 3, if deemed necessary, we will fill the African-American and female cohorts with subjects of any gender and race and will consider completing enrollment with subjects who are not ARV naïve, but who have not been taking ARV drugs for at least 6 months. This will assure we will have a substantial mix of African-Americans and women in the study, if not the exact numbers desired.
- We may consider protocol revisions to increase enrollment such as relaxing some inclusion/exclusion criteria, allowing the biopsies to be optional, or providing additional latitude in scheduling visits (as long as the scientific intent of the study is maintained).

Safety:
The study will follow the UCD guidelines that require investigators to promptly notify COMIRB (within 5 days of the occurrence) when unexpected serious AEs occur. These are events that are not listed in the consent form, and are possibly related to the intervention, or are listed but occur more frequently or are more severe than anticipated. The following clinical adverse event information will be recorded by study personnel: date of onset, assessment of severity, relationship to study medication, date of resolution/stabilization of event. The DAIDS table for grading will be used to assess severity for clinical and laboratory AEs. Dr. Wilson (and the study physician) will be alerted immediately to any grade III or higher toxicities and DAIDS, COMIRB, and the CTRC will be alerted to any SAE including: An event that results in death (regardless of relationship to study agent); a congenital anomaly, birth defect, or fetal loss (regardless of relationship to study agent); persistent or significant disabilities or incapacities (regardless of relationship to study agent); a suspected adverse drug reaction, (i.e., definitely, probably, possibly, and probably not related) that requires or prolongs existing hospitalization, or requires intervention to prevent significant/permanent disability or death; or a life-threatening (including all Grade 4 adverse events) suspected adverse drug reaction, (i.e., definitely, probably, possibly, and probably not related to a study agent).

This study will follow the all levels of reporting to DAIDS for the Expedited Adverse Event Reporting. This level of reporting to DAIDS will continue from study enrollment until study completion or study discontinuation for any reason. Furthermore, should study staff learn of any serious, unexpected, clinical suspected adverse effect after this time, the event will also be reported.

For HIV-infected volunteers: The drug therapy for HIV-infected patients in this study (TDF+FTC+EFV) is under the direct supervision of their primary physician/provider. All general laboratory and clinical adverse event data will be routinely conveyed to the primary treating provider. All clinical and laboratory AEs will be evaluated using the DAIDS grading scale. Any value ≥ Grade II according to DAIDS grading criteria will be discussed with the provider and the decision to withdraw or keep the subject in the study will be a joint one between a research team member and the subject's provider. Adverse events > Grade III will be result in study discontinuation and referral of the subject to the provider for care.

If the primary treating provider decides to discontinue or change the medication regimen the subject will be removed from the study. If the primary provider decides that for any reason the subject should no longer continue in the study, he/she will be withdrawn from the study.

The only intervention from this study is observation of drug concentrations and one cervical sample for women and one semen sample for men. One rectal biopsy will be obtained from both men and women. Accordingly, for the HIV-infected subjects, Dr.

Wilson will focus on a summary of hematology - to monitor the effects of blood draws, and any adverse outcomes related to the rectal biopsy, semen sample and cervical sample.

For HIV-negative volunteers: These subjects will take 30 days of Truvada® (TDF+FTC). During the 30 days, the HIV-negative subjects have similar blood, cervical sampling, semen sampling and rectal biopsies as the HIV-infected subjects. Hematology data and biopsy healing will be reported to Dr. Wilson, as above. The most likely toxicity during the short study timeframe is mild nausea and/or headache from Truvada® (section 2.3.2 of the protocol provides a full list of possible AEs). To reduce nausea, subjects may take the doses with food, although the pharmacokinetics visits will be in the fasting state. Analgesics will be allowed for headaches. Subjects will be seen five times while on medication during the 30 day study and clinical events with safety laboratories will be collected at each of the visits (see study evaluations, Appendix A). Subjects will be contacted by a mode of their choice once during the study between the day 7 and day 20 visits. Subjects will be followed for 30 days after completing the medication to follow laboratory values and assess the true terminal elimination phase for the study drugs. During this 30 day phase off study drug, the subjects will be seen three times and will be assessed for adverse clinical events and safety laboratories (if warranted).

In the event of a grade III or greater clinical or laboratory toxicity, the HIV-negative subject must discontinue the study drug and will be followed clinically until the abnormality falls below a grade II. Grade II clinical or laboratory toxicities will be discussed among the study physician, safety officer, principal investigator, and nurse practitioner study coordinator within 24 hours. If the abnormal laboratory or clinical adverse event is possibly related with the study drug the study team may repeat the laboratory or assessment within 72 hours. Based on the results of the repeat assessment, the participant may be allowed to continue the study if they wish to, and the study physician, safety officer, and principal investigator all agree that it is acceptably safe. A study investigator will advise the participant of the decision made with the safety officer and principal investigator and answer questions that they may have. Participants who continue study drug in these circumstances may undergo repeat assessments consistent with careful clinical monitoring.

Any adverse event ≥ grade II will be communicated to the study physician. Any > grade II abnormality will be communicated to the study physician and Dr. Wilson. All toxicities will be managed by a study team member on the protocol with collaboration with the study physician as needed. The subject will be followed until the toxicity grade decreases to less that grade II. Evaluations and the frequency of follow up visits will be determined on an individual basis in collaboration with the study physician.

Summary of Information for Safety Officer Meetings:
A report with the following data will be prepared for meetings:
X__ Clinical and laboratory adverse events (graded by DAIDS toxicity guidelines)
X__ Recruitment strategy
X__ Recruitment and enrollment statistics
X__ Gender and ethnicity statistics
X__ Disqualified and excluded individuals

The data will be "frozen" approximately one week before the Safety Officer review. The report will be sent to Dr. Wilson approximately 5 days before the meeting.

The meeting minutes will be prepared by Dr. Anderson.

The minutes from the open sessions will be sent to: Drs. Wilson, Anderson, Meditz and Julie Predhomme A.N.P., and the DAIDS Medical Monitor.

The following data will be conveyed to the SO immediately:
Any Serious Adverse Effect (SAE). Any > grade II event in the HIV-non-infected volunteers.

Stopping criteria for safety:
The study will be put on hold (no new enrollment) for any study-related SAE until the Safety Officer, study team, and DAIDS Medical Monitor agree to continue. Grade III events in the HIV-non-infected volunteers will be conveyed immediately to Drs. Meditz and Wilson, and the plan of action will be determined on a case by case basis. Medication-related SAE in HIV-infected patients will be under the direction of the primary physician.

If any of the following occurs, the study will be stopped (no new enrollment) and reassessed for safety by the study team, safety officer, and DAIDS medical monitor. 1. In the HIV-negative cohort, if three or more persons experience a grade III adverse event or two or more persons experience a grade IV adverse event. 2. Two or more persons from either cohort experience a grade IV complication from the rectal biopsy, genital sampling, or blood draws.

Reporting requirements for DAIDS:
The expedited adverse event (EAE) reporting requirements and definitions for this study and the methods for expedited reporting of SAEs to the DAIDS Regulatory Compliance Center (RCC) Safety Office are defined in "The Manual for Expedited Reporting of Adverse Events to DAIDS" (DAIDS EAE Manual), dated May 6, 2004. This manual will be followed for the timeliness and mechanisms for all reporting. SAEs will be reported on an expedited basis at the standard, intensive, and targeted levels during the Protocol-defined EAE Reporting Period, which is the entire study duration for an individual subject (from study enrollment until study completion or discontinuation of the subject from study participation for any reason).
